# Infrequently expressed miRNAs influence survival after diagnosis with colorectal cancer

**DOI:** 10.18632/oncotarget.19863

**Published:** 2017-08-03

**Authors:** Martha L. Slattery, Andrew J. Pellatt, Frances Y. Lee, Jennifer S. Herrick, Wade S. Samowitz, John R. Stevens, Roger K. Wolff, Lila E. Mullany

**Affiliations:** ^1^ Department of Medicine, University of Utah, Salt Lake City, Utah, USA; ^2^ Tulane Medical School, New Orleans, Louisiana, USA; ^3^ Department of Medicine, University of Utah, Salt Lake City, Utah, USA; ^4^ Department of Pathology, University of Utah, Salt Lake City, Utah, USA; ^5^ Department of Mathematics and Statistics, Utah State University, Logan, Utah, USA; ^6^ Department of Medicine, University of Utah, Salt Lake City, Utah, USA; ^7^ Department of Medicine, University of Utah, Salt Lake City, Utah, USA

**Keywords:** colorectal cancer, miRNAs, survival, stage, prognosis

## Abstract

Half of miRNAs expressed in colorectal tissue are expressed < 50% of the population. Many infrequently expressed miRNAs have low levels of expression. We hypothesize that less frequently expressed miRNAs, when expressed at higher levels, influence both disease stage and survival after diagnosis with colorectal cancer (CRC); low levels of expression may be background noise.

We examine 304 infrequently expressed miRNAs in 1893 population-based cases of CRC with paired carcinoma and normal mucosa miRNA profiles. We evaluate miRNAs with disease stage and survival after adjusting for age, study center, sex, MSI status, and AJCC stage. These miRNAs were further evaluated with RNA-Seq data to identify miRNA::mRNA associations that may provide insight into the functionality of miRNAs.

Eleven miRNAs were associated with advanced disease stage among colon cancer patients (*Q* value = 0.10). Eight infrequently expressed miRNAs influenced survival if highly expressed in overall CRC. Of these, five increased likelihood of dying if they were highly expressed, i.e. miR-124-3p, miR-143-5p, miR-145-3p, miR31-5p, and miR-99b-5p, while three were associated with better survival if highly expressed, i.e. miR-362-5p, miR-374a-5p, and miR-590-5p. Thirteen miRNAs infrequently expressed in colon-specific carcinoma tissue were associated with CRC survival if highly expressed. Evaluation of miRNAs::mRNA associations showed that mRNA expression influenced by infrequently expressed miRNA contributed to networks and pathways shown to influence disease progression and prognosis.

Our large study enabled us to examine the implications of infrequently expressed miRNAs after removal of background noise. These results require replication in other studies. Confirmation of our findings in other studies could lead to important markers for prognosis.

## INTRODUCTION

MiRNAs are small, non-protein-coding RNA molecules involved in the regulation of gene expression either by post-transcriptionally suppressing mRNA translation or by causing mRNA degradation [1–6]. Since associations between miRNA expression and colorectal cancer (CRC) were initially reported, [7, 8] over 2000 miRNAs have been identified and are available for study. Of these miRNAs, roughly 1300 are expressed in colorectal tissue. Most reported studies have small sample sizes and focus on miRNAs expressed commonly in the population. However, of those miRNAs expressed in CRC tissue, roughly 48% are expressed in less than half of the population [9]. These infrequently expressed miRNAs are most likely a mixture of those that are expressed at low levels, near the level of detection (background “noise”), and miRNAs that are potentially biologically relevant when expressed at higher levels although expressed less frequently in the population. Infrequently expressed miRNAs with higher levels of expression may be important for determining specific molecular pathways or to assist in identifying markers for disease progression and survival.

We have previously assessed miRNAs as they relate to CRC and survival [10, 11]. We have shown that miRNAs appear to have a greater impact for survival after diagnosis with rectal cancer than with colon cancer [11]. Our main focus in previous analyses has been on commonly expressed miRNAs although we have examined the impact of “any” expression rather for miRNAs expressed less frequently, not taking into consideration potential background noise. While we detected some associations between survival and any expression of a few miRNAs, [11] it is possible that other infrequently expressed miRNAs are important only if expressed at higher levels. We have previously shown that infrequently expressed miRNAs are useful in distinguishing specific tumor molecular phenotypes [12].

In this study, we test the hypothesis that higher levels of expression of less frequently expressed miRNAs contribute to survival after a diagnosis of CRC. We also evaluate these infrequently expressed miRNAs with AJCC stages and site-specific CRC, i.e. colon or rectal cancer, associations. To gain insight into the functionality of these miRNAs, we utilize RNA-Seq data for mRNA expression to determine relationships between the miRNAs and their associated mRNAs. Our large sample size is essential to examine the effects of higher levels of expression of less frequently expressed miRNAs on disease progression and prognosis.

## RESULTS

The population was predominately male with equal proportions of proximal and distal colon (Table 1). The majority of cases were diagnosed at AJCC stages 1 and 2. Out of a total of 2006 miRNAs assessed, 1278 or 63.7% were expressed in colorectal tumor tissue. Of those miRNAs expressed in CRC tissue, 47.8% were expressed in less than 50% of the study population with 72% being expressed in less than 10% of the study population.

Table 1Description of study population

**Table:** 

		Overall			Colon				Rectal			
		Subject *N*		%	Subject *N*		%		Subject		%	
Sex	Male	1027		54.3	607		52.8		420		56.5	
	Female	866		45.7	543		47.2		323		43.5	
Center	Kaiser	1144		60.4	740		64.3		404		54.4	
	Utah	749		39.6	410		35.7		339		45.6	
Site	Proximal Colon	581		50.5	581		50.5		0		0.0	
	Distal Colon	569		49.5	569		49.5		0		0.0	
Vital Status	Dead	914		48.3	562		49.0		352		47.4	
	Alive	977		51.7	586		51.0		391		52.6	
AJCC Stage	Stage I	559		30.0	259		22.7		300		41.5	
	Stage II	489		26.3	350		30.7		139		19.2	
	Stage III	548		29.4	340		29.9		208		28.8	
	Stage IV	266		14.3	190		16.7		76		10.5

**Table:** 

						miRNA N^1^		%		miRNA N		%		miRNA N	%
Percent Expressing in carcinoma tissue:			No Expression			728		36.3		759		37.8		882	44.0
			Any Expression			1278		63.7		1247		62.2		1124	56.0
			0 > to 10			441		34.5		412		33.0		298	26.5
			> 10 to 20			57		4.5		55		4.4		43	3.8
			> 20 to 30			39		3.1		41		3.3		38	3.4
			> 30 to 40			39		3.1		40		3.2		33	2.9
			> 40 to 50			35		2.7		35		2.8		37	3.3
			> 50%			667		52.2		664		53.2		675	60.1
						Mean		STD		Mean		STD		Mean	STD
Age						64.2		10.2		65.4		9.5		62.3	11.0

^1^miRNA expression in carcinoma tissue.

Eleven infrequently expressed miRNAs were associated with stage when assessing colon cancer cases (Table 2). Of these 11 miRNAs, the up-regulation of 10 made a more advanced disease stage more likely than a lower disease stage. Up-regulation of one miRNA, miR-1244 was associated with a lower disease stage. The majority of these miRNAs were expressed at high levels in less than 20% of the population. For rectal cancer, there were nine miRNAs associated with disease stage prior to adjustment for multiple comparisons (Supplementary Table 2).

**Table 2:** Associations between infrequently expressed miRNAs and AJCC stage for colon cancer

		Stage 1 and 2		Stage 3 and 4					*P*-value	*Q*-values
miRNA	miRNA Expression^1^	*N*	%	*N*	%	OR^2^	(95% CI)			
hsa-miR-1244	Down-regulated	6	1.0	5	0.9	0.84	(0.25,	2.79)	0.7761	0.78
	Referent	448	73.6	431	81.3	1.00				
	Up-regulated	155	25.5	94	17.7	0.63	(0.47,	0.84)	0.0017	0.10
hsa-miR-133b	Down-regulated	231	37.9	210	39.6	1.12	(0.87,	1.46)	0.3773	0.76
	Referent	340	55.8	260	49.1	1.00				
	Up-regulated	38	6.2	60	11.3	2.00	(1.29,	3.11)	0.0021	0.10
hsa-miR-143-5p	Down-regulated	33	5.4	35	6.6	1.24	(0.75,	2.05)	0.3954	0.76
	Referent	559	91.8	457	86.2	1.00				
	Up-regulated	17	2.8	38	7.2	2.64	(1.46,	4.75)	0.0013	0.10
hsa-miR-31-5p	Down-regulated	14	2.3	9	1.7	0.81	(0.35,	1.90)	0.6262	0.78
	Referent	511	83.9	415	78.3	1.00				
	Up-regulated	84	13.8	106	20.0	1.56	(1.14,	2.15)	0.0056	0.10
hsa-miR-4290	Down-regulated	33	5.4	28	5.3	1.02	(0.60,	1.72)	0.9474	0.95
	Referent	530	87.0	437	82.5	1.00				
	Up-regulated	46	7.6	65	12.3	1.73	(1.16,	2.58)	0.0076	0.10
hsa-miR-4324	Down-regulated	72	11.8	42	7.9	0.65	(0.43,	0.97)	0.0374	0.62
	Referent	470	77.2	394	74.3	1.00				
	Up-regulated	67	11.0	94	17.7	1.60	(1.13,	2.25)	0.0078	0.10
hsa-miR-466	Down-regulated	16	2.6	21	4.0	1.64	(0.84,	3.19)	0.1476	0.69
	Referent	562	92.3	459	86.6	1.00				
	Up-regulated	31	5.1	50	9.4	2.01	(1.26,	3.20)	0.0035	0.10
hsa-miR-502-3p	Down-regulated	12	2.0	6	1.1	0.59	(0.22,	1.61)	0.3046	0.73
	Referent	569	93.4	477	90.0	1.00				
	Up-regulated	28	4.6	47	8.9	2.00	(1.23,	3.26)	0.0051	0.10
hsa-miR-645	Down-regulated	6	1.0	5	0.9	1.04	(0.31,	3.46)	0.9505	0.95
	Referent	449	73.7	344	64.9	1.00				
	Up-regulated	154	25.3	181	34.2	1.51	(1.17,	1.96)	0.0017	0.10
hsa-miR-6722-5p	Down-regulated	10	1.6	11	2.1	1.32	(0.56,	3.15)	0.5276	0.78
	Referent	592	97.2	498	94.0	1.00				
	Up-regulated	7	1.1	21	4.0	3.61	(1.52,	8.58)	0.0037	0.10
hsa-miR-934	Down-regulated	22	3.6	13	2.5	0.91	(0.45,	1.87)	0.8038	0.80
	Referent	282	46.3	183	34.5	1.00				
	Up-regulated	305	50.1	334	63.0	1.70	(1.33,	2.16)	< .0001	0.10

^1^Down-regulated was defined as <-1.77 Agilent Relative Florescent Units (ARFU); the referent group was -1.77-2.08 ARFU; upregulated was miRNA expression above 2.08 ARFU.

^2^Adjusted for age, study center, and sex.

Assessment of infrequently expressed miRNAs in overall colorectal tumors with survival showed that eight miRNAs influenced survival when up-regulated in tumors (Table 3). Of these, five increased the likelihood of dying if they were up-regulated, i.e. miR-124-3p, miR-143-5p, miR-145-3p, miR31-5p, and miR-99b-5p, while three improved survival if up-regulated, i.e. miR-miR-362-5p, miR-374a-5p, and miR-590-5p. As with AJCC stage data, these miRNAs were infrequently highly expressed, except for miR-99b-3p, whose high differential expression was observed for 34% of the population. This high differential is from lack of expression in normal tissue, but higher levels of expression in carcinoma tissue.

**Table 3:** Associations between infrequently expressed miRNAs and overall survival after diagnosis with colorectal cancer

		Censored	Died of CRC				*P*-value			
miRNA	miRNA Expression^1^	*N*	%	*N*	%	HR^2^	(95% CI)		Raw	*Q*
hsa-miR-124-3p	Down-regulated	70	5.4	54	9.5	1.25	(0.94,	1.66)	0.127	0.58
	Referent	1184	92	488	85.9	1.00				
	Up-regulated	33	2.6	26	4.6	1.93	(1.30,	2.86)	0.001	0.15
hsa-miR-143-5p	Down-regulated	73	5.7	31	5.5	0.78	(0.53,	1.12)	0.179	0.58
	Referent	1177	91.5	489	86.1	1.00				
	Up-regulated	37	2.9	48	8.5	1.70	(1.26,	2.29)	0.001	0.15
hsa-miR-145-3p	Down-regulated	29	2.3	18	3.2	1.22	(0.76,	1.97)	0.408	0.61
	Referent	1252	97.3	535	94.2	1.00				
	Up-regulated	6	0.5	15	2.6	3.44	(2.04,	5.78)	< .0001	0.15
hsa-miR-31-5p	Down-regulated	19	1.5	7	1.2	0.64	(0.30,	1.36)	0.245	0.59
	Referent	1114	86.6	455	80.1	1.00				
	Up-regulated	154	12	106	18.7	1.38	(1.11,	1.70)	0.003	0.15
hsa-miR-362-5p	Down-regulated	27	2.1	11	1.9	0.64	(0.35,	1.17)	0.148	0.58
	Referent	912	70.9	439	77.3	1.00				
	Up-regulated	348	27	118	20.8	0.70	(0.57,	0.86)	0.001	0.15
hsa-miR-374a-5p	Down-regulated	16	1.2	7	1.2	0.72	(0.34,	1.54)	0.4	0.61
	Referent	996	77.4	478	84.2	1.00				
	Up-regulated	275	21.4	83	14.6	0.63	(0.50,	0.80)	< 0.0001	0.15
hsa-miR-590-5p	Down-regulated	171	13.3	79	13.9	0.93	(0.74,	1.19)	0.578	0.62
	Referent	963	74.8	448	78.9	1.00				
	Up-regulated	153	11.9	41	7.2	0.62	(0.45,	0.86)	0.004	0.15
hsa-miR-99b-5p	Down-regulated	289	22.5	121	21.3	1.01	(0.81,	1.27)	0.935	0.94
	Referent	634	49.3	254	44.7	1.00				
	Up-regulated	364	28.3	193	34	1.33	(1.10,	1.61)	0.004	0.15

^1^Down-regulated was defined as <-1.77 Agilent Relative Florescent Units (ARFU); the referent group was -1.77-2.08 ARFU; upregulated was miRNA expression above 2.08 ARFU.

^2^Adjusted for age, study center, sex, and AJCC Stage.

Evaluation of site-specific associations with survival, showed that thirteen miRNAs infrequently expressed in colon cancer-specific tissue were associated with CRC survival if up-regulated (Table 4). Eleven of these miRNAs were associated with a greater likelihood of dying when up-regulated, while two miRNAs, miR-632 and miR-362-5p, were associated with better survival if up-regulated. For rectal cancer, 26 miRNAs were associated with survival prior to adjustment for multiple comparisons (data not shown in table). Of these, 11 had a *Q* value of 0.25 or less implying that potentially 25% of the findings were false positives. Of those miRNAs associated with site-specific survival prior to adjustment for multiple comparisons, only four miRNAs were associated with survival for both colon and rectal cancers, miR-124-3p, miR-2278, miR-4300, and miR-548aa. Of these, miR-548aa and miR-2278 were associated with increased likelihood of dying if up-regulated in colon tissue while increasing the likelihood of survival if highly up-regulated in rectal tissue. After adjustment for multiple comparisons in rectal tumors, the *Q* values were higher than 0.15 (Supplementary Table 3 shows results for rectal cancer). Results were similar with adjustment for MSI, although there was slightly less power (Supplementary Table 4 shows results adjusted for MSI).

**Table 4:** Infrequently expressed miRNAs associated with survival after diagnosis with colon cancer

		Censored		Died of CRC					*P*-value	
miRNA	miRNA Expression^1^	*N*	%	*N*	%	HR^2^	(95% CI)		Raw	*Q*
hsa-miR-1	Down-regulated	33	4.2	18	5.2	0.78	(0.48,	1.28)	0.325	0.96
	Referent	748	94.7	310	90.1	1.00				
	Up-regulated	9	1.1	16	4.7	1.97	(1.18,	3.28)	0.009	0.15
hsa-miR-124-3p	Down-regulated	55	7	39	11.3	1.15	(0.82,	1.61)	0.427	1.00
	Referent	713	90.3	287	83.4	1.00				
	Up-regulated	22	2.8	18	5.2	2.19	(1.35,	3.55)	0.001	0.15
hsa-miR-133a	Down-regulated	23	2.9	15	4.4	0.90	(0.53,	1.53)	0.688	1.00
	Referent	760	96.2	315	91.6	1.00				
	Up-regulated	7	0.9	14	4.1	1.98	(1.16,	3.40)	0.013	0.15
hsa-miR-143-5p	Down-regulated	48	6.1	20	5.8	0.69	(0.43,	1.10)	0.119	0.91
	Referent	720	91.1	291	84.6	1.00				
	Up-regulated	22	2.8	33	9.6	1.75	(1.22,	2.52)	0.003	0.15
hsa-miR-145-3p	Down-regulated	18	2.3	12	3.5	1.12	(0.62,	2.03)	0.704	1.00
	Referent	767	97.1	319	92.7	1.00				
	Up-regulated	5	0.6	13	3.8	3.21	(1.82,	5.64)	<.0001	0.15
hsa-miR-31-5p	Down-regulated	18	2.3	5	1.5	0.56	(0.23,	1.37)	0.203	0.91
	Referent	659	83.4	264	76.7	1.00				
	Up-regulated	113	14.3	75	21.8	1.43	(1.10,	1.85)	0.007	0.15
hsa-miR-3622b-3p	Down-regulated	23	2.9	9	2.6	1.05	(0.54,	2.05)	0.879	1.00
	Referent	590	74.7	240	69.8	1.00				
	Up-regulated	177	22.4	95	27.6	1.41	(1.11,	1.79)	0.005	0.15
hsa-miR-362-5p	Down-regulated	16	2	7	2	0.59	(0.28,	1.27)	0.176	0.91
	Referent	585	74.1	275	79.9	1.00				
	Up-regulated	189	23.9	62	18	0.70	(0.53,	0.92)	0.012	0.15
hsa-miR-378g	Down-regulated	156	19.7	77	22.4	1.15	(0.89,	1.49)	0.292	0.96
	Referent	586	74.2	238	69.2	1.00				
	Up-regulated	48	6.1	29	8.4	1.86	(1.26,	2.74)	0.002	0.15
hsa-miR-548aw	Down-regulated	21	2.7	11	3.2	1.01	(0.55,	1.84)	0.977	1.00
	Referent	763	96.6	326	94.8	1.00				
	Up-regulated	6	0.8	7	2	2.97	(1.40,	6.34)	0.005	0.15
hsa-miR-632	Down-regulated	142	18	52	15.1	0.74	(0.55,	0.99)	0.045	0.91
	Referent	528	66.8	248	72.1	1.00				
	Up-regulated	120	15.2	44	12.8	0.66	(0.48,	0.91)	0.012	0.15
hsa-miR-645	Down-regulated	5	0.6	6	1.7	3.14	(1.37,	7.16)	0.007	0.59
	Referent	576	72.9	213	61.9	1.00				
	Up-regulated	209	26.5	125	36.3	1.32	(1.06,	1.65)	0.014	0.15
hsa-miR-671-3p	Down-regulated	42	5.3	22	6.4	1.27	(0.82,	1.96)	0.277	0.96
	Referent	727	92	310	90.1	1.00				
	Up-regulated	21	2.7	12	3.5	2.32	(1.30,	4.16)	0.005	0.15

^1^Down-regulated was defined as <-1.77 Agilent Relative Florescent Units (ARFU); the referent group was -1.77-2.08 ARFU; upregulated was miRNA expression above 2.08 ARFU.

^2^Adjusted for age, study center, sex, and AJCC Stage.

Evaluation of the 24 miRNAs most strongly associated with either survival or disease stage (from Tables 2–4) also showed significantly (FDR < 0.05) altered differential gene mRNA expression for 14 of the miRNAs examined (Table 5). Five of these miRNAs, miR-1, miR-133a, miR-145-3p, miR-3622b-3p, and miR-6722-5p, were associated with fewer than five genes that were differentially expressed in colorectal tissue. Four of the miRNAs, miR-31-5p, miR-362-5p, miR-934, and miR-99b-5p, were associated with expression of over 100 genes in colorectal tissue. Over 100 of the genes associated with these miRNAs were associated with two to four miRNAs. This can be seen also in the overlap in functions of genes associated with these miRNAs.

**Table 5:** miRNAs associated with survival or disease stage (*Q* value < 0.15), their targeted genes and associated pathways

miRNA	Genes associated with miRNAs in colorectal tissue using RNAseq mRNA expression data^1^	IPA Top Diseases and Functions^2^
hsa-miR-1	*HAND1*	
hsa-miR-133a	*CHRDL2, GRIK5*	
hsa-miR-133b	*HSPB6, PER3, MYLK, PDZD4, LIMS2, SPEG, TACR2, TNS1, PGR, SORBS1, GRIK5, HAND1, LDB3, NECAB1, CNN1, AOC3, MYH11, MEIS2, FXYD6, MYOCD, BOC, TAGLN, JPH2, GPM6A, SPARCL1, CACNA2D1, KCNMA1, ADAMTSL3, ACTG2, LMOD1, LONRF2, SYNPO2, HSPB7, DES, CHRM2, MAB21L2, SYNM, DACT3, DMD, RP11-728F11.6*	Cancer, Organismal Injury and Abnormalities, Reproductive System Disease
hsa-miR-143-5p	*HSPB6, MYLK, TNS1, SORBS1, GRIK5, HAND1, NECAB1, CNN1, MYH11, SPARCL1, KCNMA1, ACTG2, LMOD1, SYNPO2, DES, CHRM2, SYNM, DACT3, PLN*	Cardiovascular System Development and Function, Developmental Disorder, Embryonic Development
hsa-miR-145-3p	*HAND1, CHRM2*	
hsa-miR-31-5p	*PRSS22, SOX8, ASB4, MAP3K9, ETV1, INSRR, VRK2, RAB27B, BARX2, FOXC1, DCBLD2, RIMBP2, ST3GAL6, C9orf30, PTGS2, FSCN1, SLC6A14, GP6, LYZ, GABRP, BAMBI, MIOX,****PXMP4****, STS,* ***KIAA0226L****, PLLP,* ***QPRT****, ERI1, PON3, CAV2, HOXA13, EPHB6, AGR2, CDR2L, GABRA4, ST3GAL4, NT5DC3, VNN1, PDE10A, IMPG1, FAM46A, AADAC, OTX1, MLPH, EPHA4, QSOX1, PLA2G4A, C1orf114, TNNT2, B4GALT6, ONECUT2, DNAL1, CD274, DUSP4,* ***NEURL2****, C11orf9, PIWIL1, BMP4, TNFSF9, SRD5A3, FAM40B, FEZF1, GAD1, KLK10,* ***POPDC3****, NMUR2, MORC4, TTC9, REG4,* ***VAV3****,* ***HMGCS2****, VTCN1, GRHL1, KCTD1, SLCO1B1, ANXA1, NT5E,****SLC19A3****, SCEL, GALNT5, STXBP1, TFAP2A, SHF, PHLDA1,****SLC39A5****, ZIC5, PVRL4, ABCA12, PLK2, MEGF10, TRIM7, TPBG, SYTL5, DOCK5, SLC7A11, TEX9, AP1S3, PRDM8, GPR110, FAM167A,* ***SLC26A2****, CD109, CLDN12, FGF17, PLA2G4D, TFF1, TMPRSS3, PAQR6, AQP5, MSLNL, KCNJ3, SLC16A14, SPRR3, ANTXR2,* ***FABP1****, HSPA4L, KIAA0895, TRPV6,* ***CDX2****, BTNL9,****ANPEP****, PPP1R29,****B4GALNT2****, AQP2, KLK11, CCDC165,* ***GJB1***,***DRD5****, MUC17, TM4SF4, SDR16C5, KRT15, HOXC5, ABLIM3, AGR3, RTTN, CA8,* ***EXOC3****, ARSJ, HOXC9, IGSF5, AC010336.1, B3GALT5,****FAM3B****, FOXD1, TACSTD2,* ***MAP7D2****, MUC6, IFNE,* ***ANO9****, C14orf80, LEMD1, VSIG10L, CIDEC,****LHFPL3****,* ***CTD-2228K2.5***,***C10orf99****, CTSE, DAPK1,****CYP2B6****, SLC28A3, SERPINB2, HOXC6, HOXC4, F5, GLT25D2, SAMD5, TBC1D8, IGFL2, FAM116B, ONECUT3, SERPINB5,* ***POU5F1B****, CRIP1, XKR9, ETV5,****CEBPA****, RP11-79P5.6, TRNP1, ZNF432*	Cancer, Gastrointestinal Disease, Organismal injury and Abnormalities; Cancer, Organismal injury and Abnormalities, Immunological Diseases; Connective tissue Development and Function, Connective Tissue Disorders, Organ Morphology; Humoral Immune Response
hsa-miR-3622b-3p	LHFPL4	
hsa-miR-362-5p	*TSPAN6, DPM1, LAS1L, CD99, HCCS, SLC25A5, LAMP2, GGCT, GTF2IRD1, PNPLA4, NOX1, FAM76A, MBTPS2, PRICKLE3, DNASE1L1, CXorf56, C20orf43, GLRX2, PHF20, TOMM34, MIPEP, SARS, IFT88, OTC, PSMA4, GEMIN8, CTPS2, ATP6V1H, SCML1, NOP16, ZNF280C, GYG2, ZNF275, MTMR1, SLC9A7, ZNRD1,****ACSM2B****, PHKA1, IDH3G, HYAL2, OTUD5, GPKOW, GRIPAP1, FTSJ1, REEP1, ATP1B3, AP3M2, ATP6AP1, FAM50A, FAM3A, HSD17B10, SEMA3C, GPC4, CTTNBP2, SLC25A43, UBE2A, ARAF, VDAC3, KIAA0020, RRAGB, PPIE, ATRX, WDR47, CPNE3, IPO11, TXLNG, HUWE1, TPX2, PDRG1, IGBP1, HEPH, RBM41, EFNB1, KIF4A, AC013461.1, COMT, HDAC6, UPRT, SYDE2, RANBP1, SNRPD3, SLC35E4, ADRBK2, HIRA, GCAT, MCAT, VRK1, PLTP, ABHD12, PROCR, IFT52, CSTF1, TH1L, SLCO4A1, TCFL5, C20orf11, HM13, CRNKL1, KIF3B, SNTA1, TTI1, AHCY, PSMD10, TBL1X, POLA1, MID1, NKAP, NXT2, PRPS2, MOSPD1, AMMECR1, WDR13, SUV39H1, XIAP, STAG2, ATP11C, CCDC22, NAA10, ASB9, ZC3H12B, RBBP7, SLC25A14, FMR1, SLC35A2, EMD, TAZ, MAGT1, UBL4A, CD99L2, RP2, PHF16, CDK16, HTATSF1, GABRE, MAGED2, RBM3, CXorf26, GLA, KPNA3, CCDC113, CLCN7, PYCARD, CDIPT, TTC35, ARHGEF10, RNASEH2A, MRPL4, GTPBP10, PMPCB, MET, SSBP1, LSM5, MOGAT3,****SH3GL2****, ANKRD26, GLRX3, ZNHIT3, RNF43, C17orf75, PSMD11, AARSD1, USP46, FRG1, CD81, ARPC3, NUP107, RNF8, NNT, FAM172A, NIT2, PDCD10, ABHD14B, UXS1, ABCB6, MRPL37, RRAGC, RPF1, SSX2IP, DIEXF, ZNF430, RPN2, FAM98A, SLC25A2, HSPH1, ZFP30, CCDC53, PLBD1, PRDX4, NLN, MORF4L2, FAM199X, RAP2C, CKS2, CSE1L, MOCS3, POF1B, USP9X, SNRPC, UNC5CL, KLHL31, EEF1E1, UPF3B, RNF113A, THOC2, EIF2S2, ERGIC3, ROMO1, KDM5C, AMOT, PCID2, UXT, ELK1, TIMM17B, TRMT5, SLC10A3, HNRNPH2, TIMM8A, RNF6, TTI2, HDHD1, GNL3L, PGLS, EIF2S3, DKC1, MPP1, LRP3, COX7B, RLIM, ABCB7, ZFYVE20, PDHA1, FIGNL1, HRSP12, ERAL1, DPH2, ZSWIM3, RFC3, RNF128, COX16, CCNB1, RBMX2, MST4, BIVM, CARS2, C7orf68, RINT1, KIAA1009, ESPL1, SMYD5, AGT, ABCB10, RNASEH2B, SUCLA2, RCBTB1, ACTL6A, SMC2, SLC2A8, C9orf123, RANBP6, PLAA, GGH, TGS1, RTCD1, LRPPRC, DTNB, ATIC, OLA1, TMEM117, MTMR6, ESD, ITGB3BP, MRPL24, NPHP1, ITGA9, NHP2, CYP39A1, SRCRB4D, NCAPG2, CASK, SPIN2A, ZNF673, NDUFB11, ZMYM3, TAF1, NONO, CCDC120, EBP, SNX12, DIAPH2, DOCK11, FAM58A, NSDHL, CETN2, RPL10, EIF3H, WDR31, COMMD7, DSN1, MPP7, GRIN2B, SAP18, SRFBP1, C9orf150, ACSS1, APOOL, ATP6V1C1, VBP1, RPGR, RPL30, FAM122B, HKDC1, PHF6, UTP14A, AIFM1, NSMCE2, ZFYVE9, TMEM164, TAB3, C12orf43, BRE, CUL4B, AUTS2, SHROOM4, HIST1H4H, TSR2, SPATA2L, VMA21, G6PD, AZGP1, PSMC2, YDJC, HENMT1, DHX57, KBTBD8, CADPS, MAD2L1, UTP15, FOXQ1, ZNF12, ORC5, PDP1, WBSCR27, MID1IP1, PIGA, ATP7A, BRWD3, WRN, FAAH2, ENOX2, STOX1, FUNDC2, PRDX2, C7orf11, TCTN2, COMMD8, SLC25A6, APEX2, CCDC126, MMGT1, GJB1, APLF, REPS2, SYAP1, TMEM192, NANP, JAGN1, C1GALT1C1, NAIF1, CLCN5, LRRC8D, PAH, RPS21, TP53RK, PHF8, LRRN3, MRP63, ZNF449, EIF1AX, TNK1, ZHX3, LIG4, C3orf33, DLEU1, ANO6, ZBTB33, TMEM187, ZBTB41, PPP1R42, KLHL11, SEPHS2, DCTPP1, TMEM150B, MED14, PPA1, ZNF443, SSR4, PITPNB, MRPL14, PJA1, FANCB, YIPF6, C5orf30, ARL6IP4, ATP6AP2, C8orf33, XKRX, MTCP1NB, ZNRF3, SFXN4, ASCL2, KCTD16, PRKX, SMTN, FAM120C, IRAK1, MAP7D2, SS18L1, MED12, FAM123B, OSBP2, ZNF74, C12orf48, BRCC3, MYBL1, CXorf38, BCAP31, LAMP1, TMLHE, WWOX, ZNF75D, NKRF, CYP4F3, SPIN4, SPIN2B, ERCC6L, UBQLN2, COMMD6, CHM, ZDHHC9, C7orf29, ZNF567, C6orf222, ZNF140, TRAPPC2, ZNF585A, LAGE3, ZNF138, GTF2E2, ZNF81, ZNF790, RPS4X, ZNF84, NUP62CL, ZXDA, HIST1H4I, ZXDB, ZNF280B, ZNF627, TTC37, SLC9A6, BHLHB9, RPL39, HIST1H3H, RP11-451M19.3, FAM127B, RPA4, SPIN3, NEU1, C6orf48, AC021218.2, LRRC10B, DNAJC19, GTF2H4, CPNE1, AC073346.2, MTCP1, AC009403.2, TIGD1, ZNF630, ZNF717, C19orf79, MCTS1, TMEM238, RDH14, C7orf36, RPL36A, AMACR, C22orf39, KIAA0415, CEBPA, H2AFJ, LY75-CD302, AC068533.7, RP11-598P20.5*	Cell Cycle, Cell Morphology, Cellular Assembly and Organization; Developmental disorder, Hereditary Disorder, Neurological Disease; Hereditary Disorder, Organismal Injury and Abnormalities, Neurological Disease; Amino Acid Metabolism, Cell Signaling, Cellular Function and Maintenance
hsa-miR-374a-5p	*DPM1, LAS1L, PNPLA4, ZFP64, C20orf43, GYG2, CDON, REEP1, CTTNBP2, BCORL1, PDRG1, PES1, ABHD12, R3HDML, NDRG3, PABPC1L, PSMA7, KIF3B, MAP1LC3A, MOSPD1, ZC3H12B, FMR1, PHF16, GABRE, KRT23, PTK7, TGIF2, LYPLA1, PARD6B, MOCS3, EREG, ZC4H2, TTI2, ZNF337, DKC1, LRP3, GGT7, RAI2, HRSP12, CTNNBL1, ZSWIM3, BEX2, APCDD1, WIF1, PHF6, SHROOM4, KALRN, BBS5, FOXQ1, LARP6, GNG4, NANP, SCAND1, TP53RK, C2orf54, LRRN3, NCKAP5, LDLRAD3, YIPF6, FAM123B, ZNF74, TMPRSS6, C11orf95, CHM, ZDHHC9, GSPT2, PCMTD2, CPNE1, C22orf29, EIF6*	Cell Cycle, Cancer, Cell Death and Survival; Organismal Survival, Cancer, Organismal Injury and Abnormalities
hsa-miR-4324	***ANLN, CYBRD1, LIMS2, CHRDL1, MGP, CASQ2, FXYD6, BOC, CMYA5, RNF150, PODN, C7orf10, ZIM2***	
hsa-miR-645	*HECW1, MRC2, SLC11A1, TIMP2, COL11A1, BCAT1, FAP, ADAMTS2, MMP2, TREM2, MMP11, GGT5, SYNDIG1, CLEC11A, COMP, SFRP4, AEBP1, COL1A1, PRRX1, PLXDC2, COL10A1, COL5A1, POSTN, ADAMTS7, SULF1, ITGA11, COL6A2, ADAM12, ADAMTS12, SPOCK1, MMP14, MRAS, KIF26B, COL6A3, COL1A2, FNDC1, CTHRC1, CERCAM, RAB31, COL3A1, ANTXR1, HOPX, OLR1, SPHK1, GAS1, BGN, NTM, THBS2, PPAPDC1A, COL5A2, C1QTNF5, ZNF469, MFRP, SCARF2*	Connective Tissue Disorders, Organismal Injury and Abnormalities, Cellular Assembly and Organization
hsa-miR-6722-5p	***RASL12, MAD2L1BP, B3GNT1, SLC25A20***	
hsa-miR-934	*HECW1, MRC2, SLC11A1, TIMP2, VCAN, TNC, COL11A1, BCAT1, LZTS1, TRO, MOV10L1, GLI2, NOTCH3, NUAK1, FAP, EPYC, NOX4, ADAMTS2, MMP2, ZFHX4, MMP11, GGT5, TIMP3, NKAIN4, ISM1, SYNDIG1, MXRA5, WISP1, SYDE1, MEIS3, COMP, WNT2, PCOLCE, SERPINE1, SFRP4, AEBP1, DNM1, COL1A1, COL12A1, MDFI, SPARC, PDGFRB, COL7A1, PRRX1, ST6GALNAC5, MFAP2, RGS4, SPP1, LTBP2, PLXDC2, INHBA, TWIST1, PLAU, COL10A1, GLIS2, A4GALT, KRT17, ISLR, PXDN, COL5A1, PODNL1, POSTN, LOXL2, ADAMTS7, ANGPTL2, SULF1, ITGA11, BRDT, EMILIN1, ADAMTS14, LUM, HAPLN3, MFGE8, KIFC3, CDH11, RGS16, CTSK,* ***SELENBP1****, COL8A1, PDGFC, ADAM12, SERPING1, ODZ4, ADAMTS12, KCNE4,* ***SH2D6****, SPOCK1, GUCY1A2, THY1, DKK2, MMP14, DGKI, SPON2, PLXDC1, PDPN, OLFML2B, KIF26B, COL6A3, GPX8,* ***BMPER****, COL1A2, FNDC1, CTHRC1, KIAA1462, HTRA1, GPR176, FBN1, GREM1, CERCAM, IGF2, NXN, RAB31, COL3A1, COL22A1, ANTXR1, CDH2, HTRA3, PRKCDBP, HOPX, COL24A1, COL8A2, LRRC15, OLR1,* ***AGR3****, PHLDA3, CD248, SPHK1, SOX11, AC100788.1, SSC5D, C3orf80, GAS1, F2R, BGN, NTM, CDR1, THBS2,* ***ZDHHC11****, CHSY3, FCGR3A, PPAPDC1A, COL5A2, COL15A1, ATP10A, ODZ3, FAM19A5, PLXNA4, C10orf55, C1QTNF5, ZNF469, MFRP,* ***UGT1A5****, SCARF2*	
hsa-miR-99b-5p	*CFH, TFPI, PLXND1, HSPB6, THSD7A, COPZ2, GAS7, MRC2, DCN, IGF1, WWTR1, PHLDB1, SNAI2, GPR124, SAMD4A, NRXN3, FHL1, NLRP2, EHD2, HSF2, NR1H3, VIM, TIMP2, FLT4, VCAN, FAM65A, STAU2, TNC, EPHA3, PREX2, LMO3, PER3, ELN, CHRDL2, ATP2B4, GUCY1B3, CA11, CDON, CALCRL, MYLK, EML1, TRAM1, NAV3, STON1-GTF2A1L, ABCC9, RORA, TGFBR3, GNB5, PFN2, POLB, ARHGAP10, CYBRD1, LIMS2, SPEG, EVC, NDE1, MRVI1, FERMT2, FRY, GLI2, NOTCH3, SLC24A1, ZNF532, TACR2, FGFR1, FBLN1, TNRC6C, RUNX1T1, FKBP7, TNS1, MOXD1, CHRNA3, PCDHGA2, KAT6A, BCORL1, AKR1B1, ERO1LB, MMP2, NID2, CERS4, CCDC80, COL9A3, DPYSL2, SORBS1, NRP1, HCN2, PALM, SUSD2, LGALS1, TTC28, SEPT3, TIMP3, SYNGR1, PAPLN, NFATC4, PLTP, MYL9, MYOM1, CHRDL1, SMARCA1, TIMP1, KATNAL1, C13orf33, ZNF423, CRISPLD2, ZNF174, CORO2B, RASL12, IGDCC4, CTSH, BRF2, PGCP, TRPS1, NUMBL, CLIP3, PTPRS, CARD8, CADM4, CAV2, CAV1, C7orf58, PCOLCE, RARRES2, GLI3, AEBP1, DNM1, MPDZ, ACTA2, MAP3K8, CCL2, CNTNAP1, COL1A1, HDAC5, PMP22, RAB34, MAPK10, TBC1D9, TBC1D19, KLHL5, FOLR1, ARHGEF17, LEPREL2, TSPAN11, ACSS3, TENC1, GLI1, MGP, STX2, GPR133, HCFC2, COL12A1, DSE, QKI, PTK7, LAMA4, C7, SPARC, HAND1, ARSB, THBS4, NR3C1, DPYSL3, PDGFRB, CPEB4, DBN1, GNAI2, GNB4, ZBTB47, SLC4A3, ADAM23, KCNIP3, ACVR1, PDE1A, CLIP4, LOXL3, EFEMP1, GLS, IGFBP5, IL1R1, ATF2, WLS, RGS2, OLFML3, NID1, AKT3, ST6GALNAC5, LPHN2, MFAP2, NRP2, CTGF, PKD2, CNRIP1, NKX2-3, PPP1R3C, PYROXD2, MYCT1, PLXDC2, ENOX1, GLT8D2, SCPEP1, TSHZ3, KCNJ8, PSPC1, PDZRN3, CPXM2, LDB3, CALD1, BICC1, EGR2, RASSF8, NECAB1, ITIH5, OBSL1, SLC12A4, PTGIS, TRERF1, RUNX2, C3, C20orf46, GLIS2, AHDC1, RHOJ, ARMCX1, SYNGR3, HIP1, SGCE, FLNC, SNRPN, CGNL1, ISLR, PALLD, CDKN1C, CNN1, KIF1A, PXDN, COL5A1, LAMA5, AKAP12, SYNE1, EDA2R, PDLIM4, GFPT2, AOC3, MAP1B, SERPINF1, DLG4, VPS13B, MTSS1L, SYT11, MYH10, DCLK1, POSTN, MYH11, KRBA1, LYVE1, MEIS2, TSPAN2, IL6ST, NAV1, RERG, DZIP1, C5orf13, MDFIC, BAI3, LCA5, ITGA7, NPL, FAM129A, LAMC1, PLXNC1, GPNMB, SCN7A, IL10, CCL21, DNAJB5, SULF1, FXYD6, THBS1, STRA6, ZNF280D, ARHGAP29, EMILIN1, DUSP5, CALHM2, ENTPD1, SENP7, CILP, MMRN1, PDE5A, KIAA1644, LUM, WDFY2, FRMD6, SLC38A6, FBLN5, IGF1R, BBS4, MFGE8, TGFB1I1, CDH11, MYOCD, IGFBP4, COL6A1, COL6A2, EMP3, WTIP, HSPG2, CYR61, DPT, RGL1, CTSK, DYRK3, ATP8B2, LYST, WNT9A, ETNK2, RHOB, MEIS1, GPR17, FAM171B, PHLDB2, BOC, TRPC1, SLIT2, SCD5, ANK2, SFRP2, OSMR, SSBP2, KCNMB1, PPP1R18, VIP, SDK1, AGBL3, ATP6V1B2, ERLIN2, RGS20, VLDLR, ZEB1, GAS2, SERPING1, ADAM33, TAGLN, JPH2, C10orf68, ARID5B, LATS2, ITPR1, BTBD11, DIP2C, AKAP6, WWC2, LIX1L, KCNE4, HSPB8, SETBP1, SPOCK1, JMY, PLEKHH2, SPARCL1, PELO, LGI2, RBMS1, ASAP1, CACNA2D1, THY1, TBCEL, ABCA6, PGM5, PDLIM3, NCAM2, ADAMTS1, APCDD1, FZD7, GRIP1, KIF5A, C8orf38, RBPMS, MMP14, AFAP1L1, TSPAN18, PALM2-AKAP2, MEGF11, MRAS, COLEC12, SLAMF8, CACHD1, CSRP1, C1R, STARD9, SPON2, ZNF208, GPSM1, PLXDC1, ITGA5, AMDHD2, PPAP2B, SDC3, NEXN, VCAM1, DDR2, SNED1, ACTG2, C1QTNF7, COL6A3, C3orf64, FSTL1, LMOD1, IGFBP7, FBLN2, PRICKLE2, CAMK2N2, EMCN, HAND2, GUCY1A3, FAM198B, CMYA5, COL1A2, DLC1, MED30, GEM, LETM2, SVEP1, PTCHD1, NLGN4Y, HECTD2, CFL2, ZCCHC24, PHYHIPL, VSTM4, KIAA1462, SALL2, TTC7B, PDZRN4, AMOTL1, HTRA1, JAM3, FBN1, LARP6, CLMP, DCHS1, TUB, CDYL2, PRTG, TMX3, MFAP4, AC024270.1, RIMKLB, NGFRAP1, NNMT, C16orf45, RBPMS2, VPS39, GREM1, MAP1A, PBX3, CERCAM, GPRC5B, TBC1D2B, ZNF180, CORO6, TUBA1A, ZNF528, AXL, PPP1R14A, NXN, IGFBP6, TMEM99, RAB3IL1, LTBP3, PCMTD1, RAB31, SDPR, COL3A1, SEMA4C, LCMT2, CHST14, MN1, ADRB2, SDC2, CLIC4, CCDC8, ZEB2, ANTXR1, LDB2, PCDH7, TPST1, NLGN2, RNF150, DCLK2, DENND5B, LONRF2, COMMD5, SGCD, PYGO1, GRIK1, C1orf190, KCND3, MCC, DSEL, COL24A1, PRNP, LAMB2, CCL11, ID4, NEGR1, ARNT2, SYNPO2, MANEA, EFEMP2, ANUBL1, SLFN11, CYP7B1, NBEA, MRGPRF, BNC2, RHOD, PTPRM, CSPG4, HEG1, CTSF, MSRB3, GLIS1, PODN, FZD4, DES, ARL10, BAIAP2, A2M, ARL4D, TUBB6, TRIL, FZD8, HIC1, PTRF, SUMO4, FAM20C, ZNF354C, AC100788.1, DTX3, MAF, CSRNP3, THBD, C17orf57, VWA1, SSC5D, C3orf80, LYNX1, GREM2, CHRM2, TMEM136, ZNF467, MAB21L2, PLAG1, CHST15, RGMA, SYNM, AP1S2, C1S, BGN, MXRA7, NXPH3, CSF1R, IGIP, C21orf90, GJC1, CADM1, SLC8A1, GPC6, TMEM119, CCBE1, FHL3, MEX3B, FBXL7, CBX6, OLFML1, KIRREL, SCFD2, ALDH1A3, PRKD1, PROS1, SLC24A3, FLRT2, AHNAK2, PBX1, ZFP36L1, MORF4L1, RASA3, WWOX, NUDT17, GNG2, PDE2A, ZDHHC17, MITF, DYNC2H1, BCAM, C11orf96, MAGEH1, TRPV2, LDLRAD2, COL14A1, C1orf204, S100A3, ARL4C, PRELP, APOD, FAM180A, C8orf76, FAT4, ZNF565, SRGAP2P2, ZNF34, ZNF781, LAMA2, TCF4, ZNF512B, PDLIM7, FLNA, ANXA6, KANK2, PARVA, ZNF667, SIRPA, ADH4, TPM2, PLN, FAN1, LDB1, PLXNB3, ZNF521, RP3-412A9.11, CES1, GPRASP1, DMD, C1orf95, COL5A2, COL15A1, PCDHGA1, NYNRIN, VGLL3, FAM127C, LCAT, HEXA, SNURF, C6orf174, PLXNA4, NPTXR, ARHGAP23, PCDHGC3, AQP1, PCDHGC5, ARHGEF25, TMEFF1, PLEKHO2, AKAP2, PCDHGC4, STON1, SCARF2, ECSCR, C9orf30-TMEFF1, PCDHGA12, RP11-122A3.2, PCDHGB6, PCDHGA5, PCDHGA7, PCDHGA6, PCDHGA8, PCDHGA11, PCDHGB2, PCDHGB4, PCDHGB7, PCDHGB1, PCDHGA3, RP11-728F11.6, RP3-403A15.5, AC005013.1*	Developmental Disorder, Hereditary Disorder, Immunological Disease; Cellular Development, Connective Tissue Development and Function, Skeletal and Muscular System Development and Function; Cardiovascular System Development and Function, Organismal Development, Tissue Development; Connective Tissue Disorders, Organismal Injury and Abnormalities, Developmental Disorder; Embryonic Development, Organ Development, Organ Morphology; Cellular Assembly and Organization, Cellular Function and Maintenance, Cancer; Cell-To-Cell Signaling and Interaction, Cellular Assembly and Organization, Cellular Function and Maintenance; Developmental Disorder, Organismal Injury and Abnormalities, Embryonic Development; Cardiovascular System Development and Function, Organ Morphology, Skeletal and Muscular System Development and Function; Organismal Development, Cancer Organismal Injury and Abnormalities; Cancer, Organismal Injury and Abnormalities, Reproductive System Disease; Cellular Development, Cellular Growth and Proliferation, Digestive System Development and Function; Cellular Development, Connective Tissue Development and Function, Embryonic Development; Hereditary Disorder, Organismal Injury and Abnormalities, Embryonic Development

^1^Genes significantly downregulated are in bold.

^2^Determined by IPA Networks with Scores > 20.

**Figure 1:**
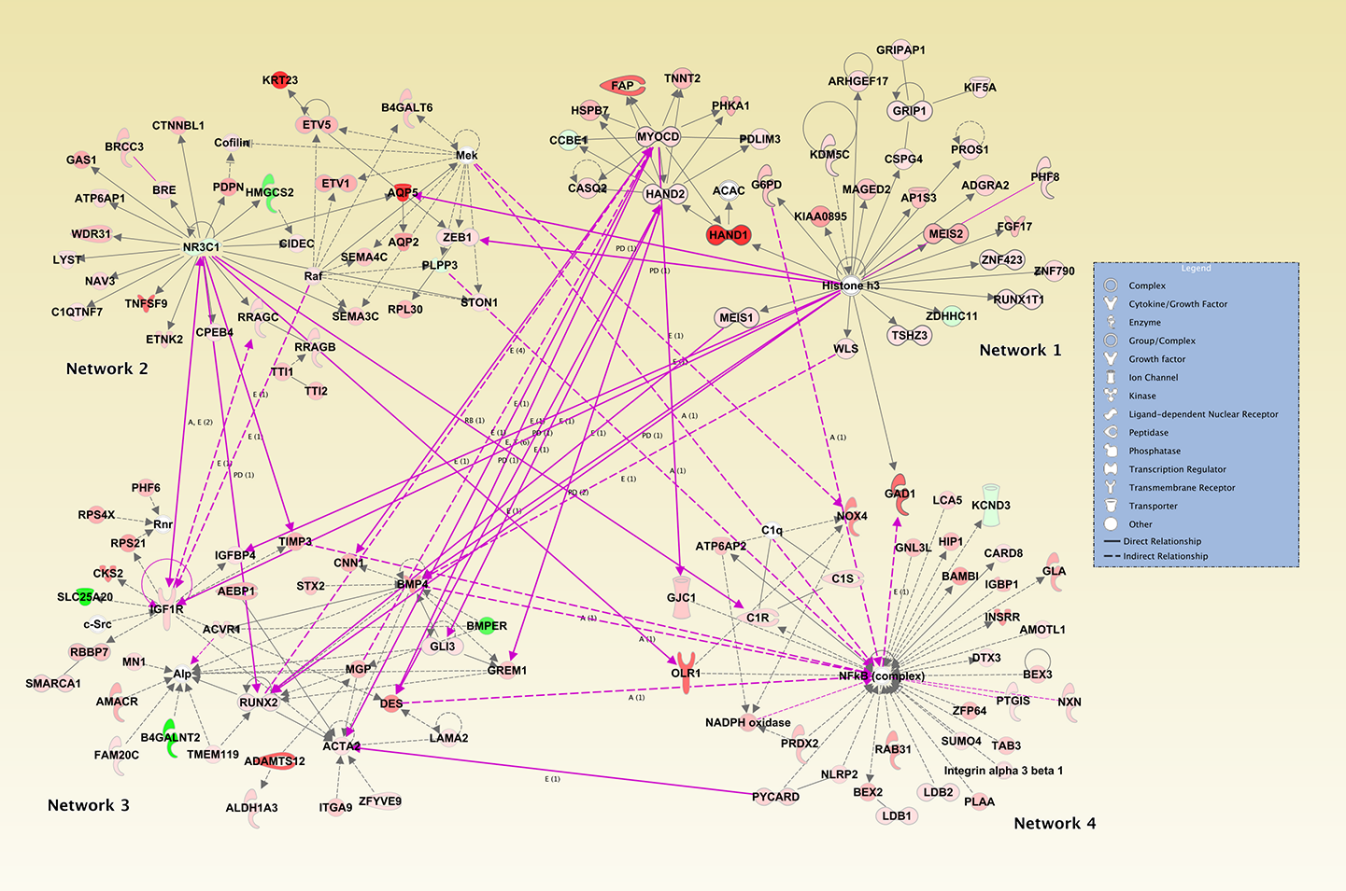
Top four IPA networks associated with genes linked to miRNAs that influence advanced disease stage or survival

When we assessed IPA networks derived from genes that were associated with all miRNAs that influenced survival, 17 networks had a score of 25 or greater Table 6). Figure 1 visually depicts the genes that are up- or down-regulated in the infrequently expressed miRNAs for the top four networks. Network 1 (Nervous system development and function, organ morphology, and organismal development) had a score of 35 with 33 focus molecules from our dataset in that network. Both Network 2 (Lipid metabolism, Molecular Transport, and Small Molecule Biochemistry) and Network 3 (Cellular development, Connective Tissue Development and Function, Skeletal and Muscular System Development and Function) had a score of 33 and 32 focus molecules of which several genes previously associated with colorectal cancer including *RUNX*, *BMP*, *IGF1R*, and *FAP*. The NFκB complex was central to the 4th Network (Developmental Disorder, Hereditary Disorder, and Immunological Disease).

**Table 6:** IPA networks derived from genes associated with miRNAs that influenced CRC survival

Network	Molecules in Network	Score	Focus Molecules	Top Diseases and Functions
1	ACAC,ADGRA2,AP1S3,ARHGEF17,CASQ2,CCBE1,CSPG4,FAP,FGF17,G6PD,GAD1,GRIP1,GRIPAP1,HAND1,HAND2,Histoneh3,HSPB7,KDM5C,KIAA0895,KIF5A,MAGED2,MEIS1,MEIS2,MYOCD,PDLIM3,PHF8,PHKA1,PROS1,RUNX1T1,TNNT2,TSHZ3,WLS,ZDHHC11,ZNF423,ZNF790	35	33	Nervous System Development and Function, Organ Morphology, Organismal Development
2	AQP2,AQP5,ATP6AP1,B4GALT6,BRCC3,BRE,C1QTNF7,CIDEC,Cofilin,CPEB4,CTNNBL1,ETNK2,ETV1,ETV5,GAS1,HMGCS2,KRT23,LYST,Mek,NAV3,NR3C1,PDPN,PLPP3,Raf,RPL30,RRAGB,RRAGC,SEMA3C,SEMA4C,STON1,TNFSF9,TTI1,TTI2,WDR31,ZEB1	33	32	Lipid Metabolism, Molecular Transport, Small Molecule Biochemistry
3	ACTA2,ACVR1,ADAMTS12,AEBP1,ALDH1A3,Alp,AMACR,B4GALNT2,BMP4,BMPER,c-Src,CKS2,CNN1,DES,FAM20C,GLI3,GREM1,IGF1R,IGFBP4,ITGA9,LAMA2,MGP,MN1,PHF6,RBBP7,Rnr,RPS21,RPS4X,RUNX2,SLC25A20,SMARCA1,STX2,TIMP3,TMEM119,ZFYVE9	33	32	Cellular Development, Connective Tissue Development and Function, Skeletal and Muscular System Development and Function
4	AMOTL1,ATP6AP2,BAMBI,BEX2,BEX3,C1q,C1R,C1S,CARD8,DTX3,GJC1,GLA,GNL3L,HIP1,IGBP1,INSRR,Integrin alpha 3 beta 1,KCND3,LCA5,LDB1,LDB2,NADPH oxidase, NFkB (complex), NLRP2,NOX4,NXN,OLR1,PLAA,PRDX2,PTGIS,PYCARD,RAB31,SUMO4,TAB3,ZFP64	31	31	Developmental Disorder, Hereditary Disorder, Immunological Disease
5	A2M,AKR1B1,amylase,ANPEP,APCDD1,AQP1,ARL4C,CCDC80,CEBPA,CES1,CYP2B6,FHL1,FZD7,GG, GJB1,HDL,HSPA4L,HSPH1,KCNMA1,KCNMB1,LCAT,MIOX,MMRN1,Nr1h,OTC,PLXND1,PPP1R14A, PTK7,SERPINF1,SPRR3,THBS1,TRPS1,VGLL3,VLDL-cholesterol,VLDLR	31	31	Cardiovascular System Development and Function, Organismal Development, Visual System Development and Function
6	ABCB6,ADAM12,AHNAK2,C11orf96,CCL2,CCL11,CDKN1C,CDX2,Ctbp,CYP39A1,DKK2,EVC,FOXQ1,GLI1,GLI2,GREM2,HIC1,KRT17,LZTS1,MTMR1,NRXN3,PDLIM7,PDZRN3,PI3K (family),REG4,SERPINE1,SLC11A1,Smad,Smad2/3,SPARCL1,SPP1,SYNE1,TCF4,WWTR1,XIAP	31	31	Cellular Movement, Cellular Development, Cellular Growth and Proliferation
7	ADAM33,ADAMTS2,Alpha catenin,C3orf80,CD99,Cg,COL5A1,COL5A2,COL6A1,COL6A2, COL6A3,DCHS1,DPT,EPHA3,estrogen receptor,FAM198B,GGCT,HOPX,KCNJ3,LRRN3, LUM, MEGF10,MMP11,NPL,NRP1,OSMR,PCDH7,PDE5A,PLAU,RGS2,TENM4,TMEFF1,TNC,TRO,Vegf	31	31	Connective Tissue Disorders, Organismal Injury and Abnormalities, Cell Morphology
8	ADAMTS1,ARHGEF25,CALCRL,COL10A1,CTGF,DGKI,DNM1,EFEMP2,ELN,EMCN,EMILIN1,ERK1/2,FBLN1,FBLN2,FBLN5,FBN1,FEZF1,GPSM1,GRK3,ITGA7,LTBP3,MFGE8,NEU1,NKX2-3,NOP16,NPHP1,PFN2,Rock,SH3GL2,SMAD1/5,Smad1/5/8,Sphk,SULF1,TNS1,VRK2	29	30	Tissue Development, Connective Tissue Disorders, Dermatological Diseases and Conditions
9	Actin,ACTL6A,BTBD11,CCNB1,DCTPP1,DLG4,EIF1AX,FHL3,FMR1,GRIK5,HCN2,Histone h4,HTATSF1,KIF1A,LRRC8D,mediator,MORF4L1,MORF4L2,NEGR1,NLGN2,Notch,PBX3,PCDHGC3,PDHA1,POLB,RNA polymerase II,SLC25A5,SMYD5,SNAI2,SRFBP1, SUCLA2,TRPV6,TUBB6,TXLNG, VPS39	29	30	Cell-To-Cell Signaling and Interaction, Cellular Assembly and Organization, Cellular Function and Maintenance
10	Akt,atypical protein kinase C,CAV2,CHRNA3,COL7A1,CSE1L, CTTNBP2,CYR61, DCBLD2, DCLK1, ECSCR, FZD8,GEM,IGFBP5,MID1,Muscarinic cholinergic receptor, PDCD10, PHLDB1, Ppp2c, PREX2,PRKCDBP,PTRF,RAB27B,SCD5,SDC2,SDPR,STRIP2,SYNM,TEAD,TFAP2A,UXS1,WDFY2,WIF1,Wnt,ZEB2	27	29	Cellular Assembly and Organization, Cellular Function and Maintenance, Cell Morphology
11	ATRX,CDH2,CLEC11A,Collagen type I,Collagen type IV,EMP3,FLNC,FLT4,Jnk,KALRN, KIF4A,Ku,LIG4,MAP1LC3A,MRC2,MYBL1,NCAPG2,OSBP2,PARP,PIWIL1,PMP22,POSTN,PP1 protein complex group,PROCR,PTPRM,RLIM,SENP7,SGCD,SGCE,SMC2,SORBS1,SUV39H1,THY1,VIM,WRN	27	29	Cell Morphology, Cell Cycle, Cellular Assembly and Organization
12	ACTG2,ADAMTS7,ASAP1,CA11,CBX6,CDK16,COMP,CRISPLD2,DYRK3,FNDC1,FSH,GPC6,GPRC5B,HDAC5,hemoglobin,HOXC6,Lh,MFAP2,MYRF,N-cor,OLFML2B,PGR,PRKX,PRRX1, RGL1,RHOB,SERPING1,SRC (family),STAT5a/b,STRA6,STS,TBL1X,TRERF1,VCAM1,VCAN	27	29	Cardiovascular Disease, Connective Tissue Development and Function, Tissue Development
13	ADCY,ADRB2,AMOT,ANTXR1,ANTXR2,ATP2B4,Beta Arrestin,Calcineurin A,Calcineurin protein(s),CCL21,CDH11,CHRM2,COL11A1,DAPK1,DDR2,EFNB1,EPHB6,ERK,GLRX3,HYAL2,JPH2,Mlc,Mmp,MYLK,NFATC4,NMUR2,NONO,NRP2,PDE10A,PDE2A,PLTP,PRDM8,RGS4,SLC8A1,WWOX	25	28	Cancer, Organismal Injury and Abnormalities, Cardiovascular System Development and Function
14	AChR,AIFM1,ASCL2,Calmodulin,CAMK2N2,CaMKII,CASK,COMT,CORO6,cytochrome-c oxidase,DTNB,EPHA4,GLRX2,GPC4,HOXA13,IGF1,ITGA11,KANK2,LARP6,MED14,Mitochondrial complex 1,NCAM2,NECAB1,NUAK1,PDGF-AA,PTPRS,RIMBP2,Sapk, SDC3,SNTA1,SOX8,TAF1, TFF1,TFPI, TSPAN2	25	28	Amino Acid Metabolism, Post-Translational Modification, Small Molecule Biochemistry
15	AKT3,Alpha Actinin,Alpha tubulin,ANLN,APOD,BAIAP2, BARX2,BCAT1,C1GALT1C1, CETN2,Ck2,DPYSL2,DYNC2H1, DZIP1,ENTPD1,F5, FERMT2,Fibrin,FLNA,FOXC1,GP6,GPIIBIIIA, GRIN2B,GUCY1A2,GUCY1A3,GUCY1B3,IFT52,IFT88,MAP1A,MAP1B,PKD2,Rap1,SSX2IP,TCR,VAV3	25	28	Cell Morphology, Cellular Assembly and Organization, Cellular Function and Maintenance
16	AP1S2,ATP6V1B2,CADM1,CERS4,CFL2,CLCA1,CLIP4,CSRNP3,CSTF1,CYP4F8,DMD,FLRT2,FOLR1,GBA,GRHL1,HSD17B10,MAP3K9,MPP6,NDE1,NNMT,NSDHL,PIP5K1B,PLK2,PPP1R3C,RGMA,RORA,S100A3,SELENBP1,SLC13A2,SLC39A5,SLC9A7,Sos,ST3GAL4,ST3GAL6,TTC7B	25	28	Developmental Disorder, Hereditary Disorder, Neurological Disease
17	14-3-3,ANXA6,ARAF,CDON,Collagen type III,CSF1R,CYP4F3,DRD5,EMD,GLIS2,GPNMB, HDAC6,Hsp27,HSPB6,IGFBP6,LOXL2,LTBP2,MYH10,MYH11,NELFCD,NOTCH3,P38 MAPK,PBX1,Pld,QKI,RANBP1,SAMD4A,Sfk,SIRPA,SMTN,STXBP1,TGFB1I1,TMSB4,tubulin (family),VRK1	24	27	Cancer, Organismal Injury and Abnormalities, Reproductive System Disease

## DISCUSSION

Our data suggest that some miRNAs, although infrequently expressed, when expressed at higher levels in tumors may impact tumor aggressiveness and prognosis. While we tested the effects of both up-regulation and down-regulation, the only significant results in Tables 2–4 were for up-regulated miRNAs such that there were higher expression levels in tumors. We observed that these infrequently expressed miRNAs were associated with CRC survival overall as well as with CRC site-specific survival. Several infrequently expressed miRNAs were more likely to have different levels of expression dependent on stage at diagnosis.

When interpreting these results, several aspects of the study need to be considered. One of the premises of the study is that low levels of expression in miRNAs infrequently expressed in the population is the result of noise in the data. This could represent miRNAs that were near the level of detection. Thus, this study specifically looks at higher levels of expression for infrequently expressed miRNAs to determine their impact on disease stage and survival. However, the determination of higher level of expression was somewhat arbitrary. We utilized the distribution of differential expression in the study population to help set these cutpoints. The upper and lower cutpoints were set at the 25th percentile and the 75th percentile of paired tumor-normal differential expression, beyond which they were considered down-regulated in carcinoma relative to normal or up-regulated in carcinoma tissue relative to normal mucosa. We further limited the analysis to only those miRNAs that had at least 30 individuals showing expression in tumors in order to have sufficient power to examine survival. Setting lower cutpoints could have increased our power, although specificity in associations could have been lost.

We previously have shown that five miRNAs were associated with survival after a diagnosis with colon cancer when any expression was evaluated. Three of these miRNAs, miR-1, miR-145-3p and miR-31-5p decreased survival when analyzed for any expression as well as for higher levels of expression in tumors in this study. HRs were similar, although slightly stronger when evaluating any expression. Analysis looking at any expression had more power than we have here looking at only higher levels of expression in tumors. However, our analyses suggest that higher levels of expression in tumors may be needed to influence prognosis for most miRNAs. This analysis is more robust when assessing infrequently expressed miRNAs because it eliminates the background noise that is present when assessing any expression. We believe that the survival associations with these miRNAs are independent of other miRNAs associated with colorectal cancer survival. Our previous analysis did not identify any miRNAs associated with colon cancer after adjustment for multiple comparisons [11], thus confounding is not likely an issue. For rectal cancer we identified several commonly expressed miRNAs that were associated with survival, while in this study we did not identify significant associations after adjustment for multiple comparisons. It is possible that lack of power is in part responsible for these lack of findings in this study given the smaller sample size of rectal cancer cases and the low frequency of exposure associated with the infrequently expressed miRNAs.

Given their infrequent expression, many of the miRNAs evaluated in our study have no previously recorded association with CRC stage or survival. However, our findings suggest that when highly expressed in a tumor, some infrequently expressed miRNAs have a potentially meaningful association with CRC progression and prognosis as indicated by disease stage and survival. Interestingly, our analysis of the mRNA expression showed that most associated genes were up-regulated with higher levels of miRNA expression in tumors than normal mucosa (Table 5). This may be counterintuitive, as miRNAs are traditionally thought to post-transcriptionally down-regulate gene expression. In addition to their role in feedback loops, many transcription factors and miRNAs are known to function in feed-forward loops (FFL) in which a given transcription factor (TF) simultaneously up-regulates or down-regulates both the miRNA and the mRNA that it targets [3]. This type of FFL is known as an ‘incoherent’ FFL, as the effect that the miRNA has on the target gene is opposite that of the TF on the target gene. As such, the miRNA and mRNA expression may appear directly associated, because the increase in transcription of the target gene by the TF can outstrip the repression of the mRNA by the miRNA. Posttranscriptional up-regulation is observed in certain cell types, such as developing germ cells, and with certain transcription factors [13]. One such FFL described in T cell acute lymphoblastic leukemia (T-ALL), involves the NFκB signaling pathway. In this FFL, NFκB activates both *CYLD*, a tumor suppressor that inhibits the nuclear translocation of NFκB, and miR-19 which suppresses *CYLD.* If NFκB upregulates miR-19, relative to *CYLD,* this pathway ultimately allows for the sustained activation of NFκB in T-ALL through the miR-19 mediated inhibition of *CYLD* [14]. The NFκB complex was one of our top networks associated with genes associated with these miRNAs.

We found that some miRNAs are associated with both stage and survival. Previously, high levels of miR-31 expression were shown to have an association with larger tumor size, poor differentiation, and advanced disease stage [15]. Moreover, high levels of miR-31 expression were associated with poorer survival [15]. This is consistent with our findings that high levels of miR-31-5p expression is associated with both CRC advanced AJCC stage and a greater likelihood of dying of CRC. Runt related transcription factor 2 (RUNX2) was associated with up-regulation by miR-99b-5p in our data. RUNX2 has been associated with Dukes staging, including liver and lymph node metastasis [16]. Additionally, we previously showed that genetic variation in *RUNX2*, a member of the TGF-b pathway, had prognostic implications [17].

To help interpret the functionality of the infrequently associated miRNAs, we linked them to gene expression data in our colorectal samples. Evaluation of genes that were either up- or down-regulated when these infrequently expressed miRNAs were expressed at higher levels, helped to identify disease functions and networks through which these miRNAs could be operating either directly or indirectly. Further examination of the top networks provides support for the functions of these miRNAs in cancer progression and prognosis. For instance, the main gene in Network 1 translates to Histone 3 (H3), a protein that comprises chromatin nucleosomes. Epigenetic methylation of various amino acids on H3 allows for increased or decreased expression of both oncogenic or tumor suppressing pathways. For example, H3K27-3me, or trimethylation of the 27th lysine residue, is found in aggressive CRC [18]. One mechanism proposed to explain the oncogenic behavior suggests that polycomb-targeted gene family members (PcG) methylate H3, thereby down-regulating tumor suppressor genes, such as Hand1, which is another member of Network 1. Demethylation of H3K4 dimethylation has been associated with CRC survival [19]. Additionally, miRNAs have been found to down-regulate PcG family members in a tumor suppressor fashion, significantly decreasing cell viability and migration of CRC cells [20]. Our study shows three miRNAs, miR-143-5p, miR-145-3p, miR-99b-5p, that are associated with Hand1 gene expression and are found to increase the likelihood of dying if highly expressed in CRC. Future studies may implicate the aforementioned miRNAs in the H3 trimethylation pathway for CRC survival and prognosis.

A central molecule in Network 2 is *NR3C1,* a glucocorticoid receptor that induces apoptotic cell death by suppressing expression of anti-apoptotic proteins, such as *BCL2* and *MCL1*, as well as up-regulating expression of pro-apoptotic proteins like BCL2-like apoptosis initiator 11 [21]. Activation of the glucocorticoid receptor has been demonstrated to inhibit invasion and cell migration through disruption of the epithelial-to-mesenchymal transition in hypoxic environments in colon cancer lines [22]. Furthermore, NR3C1 has been proposed as a marker to differentiate between CIMP-high and CIMP-low tumors [23]; CIMP status is of potential prognostic significance [24–26]. Our study shows that miR-99b-3p, whose high expression levels increases the likelihood of death in CRC, is associated with the down-regulation of *NR3C1*. This down-regulation corresponds to previous literature that shows that NR3C1 can be transcriptionally inactivated in colorectal tumorigenesis [27]. Our findings suggest that the negative survival profile associated with the dysregulation of miR-99b-3p could be in part due to the inhibition of NR3C1.

*IGF1R* is the central molecule in our third IPA pathway. IGF1R is a tyrosine kinase receptor for insulin like growth factors (IGF) 1 and 2, that is commonly overexpressed in CRC [28]. IGF1R is known to induce HIF-1a and VEGF, thus playing an important role in angiogenesis, and has been associated with CRC metastasis [29]. Moreover, crosstalk between the IGF1R and EGFR pathways has been shown to contribute to acquired resistance to EGFR inhibitors and other tyrosine kinase inhibitors [29–31]. IGF1R therefore has prognostic and survival implications for those with CRC and other IGF1R positive tumors. We found that miR-99b-5p up-regulates IGF1R and is associated with CRC survival. Thus, our findings are consistent with earlier reports and suggest that IGF1R's association with CRC survival, may, in part, be mediated by infrequently expressed miRNAs.

This study has several strengths and limitations. One of the major strengths is our ability to examine infrequently expressed miRNAs because of our large sample size. Although the sample size is one of the largest available to date, evaluation of these infrequently expressed miRNAs has an impact on the study power. We have used a *Q*-value threshold of 0.15, however given the number of associations it is likely that over 85% of them are true findings. We have been able to obtain more information on functionality of these miRNAs by comparing them to our colorectal RNA-Seq data to identify genes whose expression may be associated with these miRNAs. This has enabled us to crudely evaluate molecular pathways through which these miRNAs may be involved and to begin to understand how these miRNAs function. We encourage others to evaluate these miRNAs in both a clinical and laboratory setting to verify their association with survival as well as to gain additional insight into their functionality. Given the dates of diagnosis for these cases, it is possible that new treatment modalities may modify the results we present.

## MATERIALS AND METHODS

### Study participants

Study participants were recruited as part of two population-based case-control studies that included all incident colon and rectal cancer between 30 to 79 years of age who resided in Utah or were of the Kaiser Permanente Medical Care Program (KPMCP) in Northern California. Participants were non-Hispanic white, Hispanic, or black for the colon cancer study and also included participants of Asian race for the rectal portion of the study [32, 33] Case diagnosis was verified by tumor registry data as a first primary adenocarcinoma of the colon and were diagnosed between October 1991 and September 1994 and for rectal were diagnosed between May 1997 and May 2001. Detailed study methods have been described [9]. The Institutional Review Boards at the University of Utah and at KPMCP approved the study.

### RNA processing

Formalin-fixed paraffin embedded tissue from the initial biopsy or surgery was used to extract RNA. Carcinoma tissue and adjacent normal mucosa were used to make RNA. Cells were dissected from 1–4 sequential sections on aniline blue stained slides using an H&E slide for reference. Total RNA was extracted, isolated, and purified using the RecoverAll Total Nucleic Acid isolation kit (Ambion); RNA yields were determined using a NanoDrop spectrophotometer.

### miRNA

The Agilent Human miRNA Microarray V19.0 was used. The microarray contains probes for 2006 unique human miRNAs as described previously. Data were required to pass stringent QC parameters established by Agilent that included tests for excessive background fluorescence, excessive variation among probe sequence replicates on the array, and measures of the total gene signal on the array to assess low signal. If samples failed to meet quality standards for any of these parameters, the sample was re-labeled, hybridized to arrays, and re-scanned. If a sample failed QC assessment a second time, the sample was deemed to be of poor quality and the sample was excluded from analysis. We excluded 60 carcinoma and 80 normal samples that failed QC [9]. Our previous analysis has shown that the repeatability associated with this microarray was extremely high (*r* = 0.98), 21 and that comparison of miRNA expression levels obtained from the Agilent microarray to those obtained from qPCR had an agreement of 100% in terms of directionality of findings and that the fold change calculated for the miRNA expression difference between carcinoma and normal colonic mucosa was almost identical [10]. Of the 2006 unique human miRNAs assessed on the Agilent microarray, 1247 were expressed in colon carcinoma tissue, 1234 in normal colon mucosa, and 1147 miRNAs were expressed in both colon carcinoma and normal mucosa; 1124 miRNAS were expressed in rectal carcinoma, 1136 miRNAs were expressed in rectal normal mucosa and 1068 miRNAs were expressed in both rectal carcinoma and normal mucosa. Of the 1893 carcinoma/normal pairs available for analysis 31 were excluded from survival analysis because of lacking survival information.

To normalize differences in miRNA expression that could be attributed to the array, amount of RNA, location on array, or factors that could erroneously influence miRNA expression levels, total gene signal was normalized by multiplying each sample by a scaling factor which was the median of the 75th percentiles of all the samples divided by the individual 75th percentile of each sample [34].

### mRNA: RNA-Seq sequencing library preparation and data processing

Total RNA from 245 colorectal carcinoma and normal mucosa pairs was chosen for sequencing; 217 pairs passed quality control and are used in these analyses to identify mRNAs associated with infrequently expressed miRNAs. These samples were taken from the study subjects used for miRNA analysis and were extracted, isolated and purified as previously described [35]. RNA library construction was done with the Illumina TruSeq Stranded Total RNA Sample Preparation Kit with Ribo-Zero. The samples were then fragmented and primed for cDNA synthesis, adapters were then ligated onto the cDNA, and the resulting samples were then amplified using PCR; the amplified library was then purified using Agencount AMPure XP beads. A more detailed description of the methods can be found in our previous work [36]. Illumina TruSeq v3 single read flow cell and a 50 cycle single-read sequence run was performed on an Illumina HiSeq instrument. Reads were aligned to a sequence database containing the human genome (build GRCh37/hg19, February 2009 from genome.ucsc.edu) and alignment was performed using novoalign v2.08.01. Counts were calculated for each exon and UTR of the genes using a list of gene coordinates obtained from http://genome.ucsc.edu. Total gene counts were determined. We dropped genes that were not expressed in our RNA-Seq data or for which the expression was missing for the majority of samples [36].

### Survival and stage information

Cancer survival information was obtained from local cancer registries in Utah and California, both of which are SEER (Surveillance, Epidemiology, and End Results) cancer registries. Information on date of diagnosis and date of last contact or death were obtained in order to calculate survival months. Cause of death along with AJCC staging, based on SEER summary stage data in conjunction with pathology reports, were obtain obtained.

### Statistical methods

The study focuses on infrequently expressed miRNAs which we define as being expressed in less than 50% of the study population for either normal mucosa or tumor. To be included in the analysis, miRNAs had to have a mean level of expression of 1.0 Agilent Relative Florescent Unit (ARFU) in carcinoma or normal mucosa (to avoid low levels of background noise) and be expressed in at least 30 individuals in order to be able to evaluate survival. For reference, miRNAs expressed in over 50% of the population have a median level of expression of 30.32 ARFUs with a range of 0.18 to over 47,000 ARFU. A total of 304 miRNAs were analyzed that fit these criteria (Supplementary Table 1 provides detailed information on all of the infrequently expressed miRNAs in either colon or rectal tissue). We used paired carcinoma and normal mucosa miRNA expression, evaluating differential expression between the two tissue types to control for differences in expression by tumor site and other potential confounding factors. Each infrequently expressed miRNA could be considered expressed or not in each of tumor and normal, resulting in three primary dysregulation groups based on the tumor-normal expression differences: up-regulated (expressed more in tumor than in normal), down-regulated (expressed more in normal than in tumor), and referent (neither up- nor down-regulated beyond the 25th or 75th percentile). Rather than forcing the same number of subjects to fall into these three groups for all infrequently expressed miRNAs, cutpoints were selected based on the upper 25% and lower 25% of the tumor-normal differences for all infrequently expressed miRNAs. The resulting three-level dysregulation group factor (up, down, or referent) was used as a predictor in a per-miRNA Cox proportional hazard ratio (HR) model also adjusting for age, study center, AJCC Stage, and sex when evaluating survival. Further adjustment for MSI tumor status had minimal effect on survival associations, with hazard ratios changing less than 0.01 and *p* values being basically unaltered. Analyses were run separately for cases diagnosed with all CRC, colon cancer specifically, and rectal cancer specifically; additional adjustment for tumor site for overall CRC analysis did not appreciatively alter the results. We analyzed differences in infrequent miRNA expression between those diagnosed at AJCC stages 1 and 2 compared to those diagnosed at more advanced stages (AJCC stages 3 and 4) utilizing a logistic regression analysis adjusting for age, study center, and sex. Disease stages were combined to increase power and results from stage 1 and 2 were not significantly different. Adjustment for multiple comparisons was done using the positive false discovery rate *Q* value [37]; given the infrequent expression of these miRNAs, we report any associations for which the *Q* value was less than or equal to 0.15; a *Q* value of 0.15 would imply that up to 15% of findings could be expected to be false. We believe that these miRNAs could merit further study.

We evaluated those miRNA with a HR dysregulation group *Q* value of ≤ 0.15 (24 miRNAs) with RNA-Seq data to determine genes whose mRNA expression levels were associated with these less frequently expressed miRNAs. To determine statistical significance, we ran a Fisher-Pitman Monte Carlo test with 10,000 permutations comparing mean levels of tumor-normal gene expression differences across miRNA dysregulation groups (< 75th%tile vs > 75th%tile) in R using the ‘coin’ package [38] and applied a false discovery rate (FDR) of < 0.05 to adjust for multiple comparisons. The RPKM (Reads per Kilobase per Million) mRNA expression level data were used in these analyses. Ingenuity Pathway Analysis^®^ (IPA) was used to determine specific pathways that could be involved with genes regulated by these miRNAs with a highly stringent score criterions of 20 required; the score is calculated as –log10 P, where P is generated by a Fisher's exact test [39, 40]. Studies have found scores > 3 to be significant, with a score of 3 indicating a 1/1000 chance that the focus genes are in a network due to random chance [41–43]. Other studies have opted to utilize more stringent criteria and higher scores to ensure that their discovered networks are highly significant [40, 44]; we utilized highly stringent criteria, only including networks with scores over 20.

## CONCLUSIONS

Our large study of population-based cases of CRC enables us to evaluate associations between infrequently expressed miRNAs and stage and survival. Our data suggest that miRNAs that are infrequently expressed in the population, when expressed at high levels in CRC tumors may have an impact on metastatic potential, as indicated by disease stage as well as prognosis, as indicated by survival. Since these infrequently miRNAs are generally upregulated, higher levels of their expression in tumors could be a good indicator of prognosis. Confirmation of these findings could lead to a set of miRNAs for evaluation in tumors to help guide treatment given their impact on metastatic potential. Our data also suggest that extremely low levels of miRNA expression may have little biological significance and contribute to background noise. These infrequently expressed miRNAs when expressed at higher levels appear to be associated with expression of genes that have been linked to CRC progression and prognosis.

## SUPPLEMENTARY MATERIALS TABLES





## Abbreviations

AJCC: American Joint Committee on Cancer
ARFU: Agilent Relative Florescent Units
CRC: Colorectal Cancer
CI: Confidence Interval
FFL: Feed Forward Loop
HR: Hazard Ratio
IPA: Ingenuity Pathway Analysis
KPMCP: Kaiser Permanente Medical Care Program
miRNA: microRNA
RPKM: Reads Per Kilobase per Million
RUNX2: Runt related factor 2
SEER: Surveillance Epidemiology and End Results
QC: Quality Control
