# Supplementary material for: Infrequently expressed miRNAs influence survival after diagnosis with colorectal cancer
Source: Oncotarget. 2017 Aug 3;8(48):83845–59. doi: 10.18632/oncotarget.19863 (PMC5663559; doi:10.18632/oncotarget.19863)
Supplement: Supplementary file 1 [file oncotarget-08-83845-s001.pdf]

Infrequently expressed miRNAs influence survival after diagnosis with colorectal cancer

SUPPLEMENTARY MATERIALS

Supplementary Table 1: Description of infrequently expressed miRNAs in colon and rectal tissue.
See Supplementary_Table_1

Supplementary Table 2: Associations between miRNA and stage in rectal cancer cases

miRNA	Expression Level	Stage 1 and 2 *N*	Stage 1 and 2 %	Stage 3 and 4 *N*	Stage 3 and 4 %	OR	(95% CI)		*P*-value	*Q*-values
hsa-miR-133b	<-1.77	301	68.6	210	73.9	1.45	(1.00,	2.09)	0.050	0.977
	[–1.77, 2.08]	114	26.0	55	19.4	1.00				
	> 2.08	24	5.5	19	6.7	1.65	(0.83,	3.28)	0.152	0.988
hsa-miR-28-3p	<-1.77	43	9.8	26	9.2	0.87	(0.52,	1.46)	0.600	0.977
	[–1.77, 2.08]	359	81.8	246	86.6	1.00				
	> 2.08	37	8.4	12	4.2	0.49	(0.25,	0.96)	0.037	0.988
hsa-miR-28-5p	<-1.77	65	14.8	39	13.7	1.02	(0.66,	1.58)	0.935	0.977
	[–1.77, 2.08]	330	75.2	202	71.1	1.00				
	> 2.08	44	10.0	43	15.1	1.63	(1.03,	2.58)	0.037	0.988
hsa-miR-3150b-3p	<-1.77	39	8.9	37	13.0	1.69	(1.04,	2.76)	0.035	0.977
	[–1.77, 2.08]	320	72.9	186	65.5	1.00				
	> 2.08	80	18.2	61	21.5	1.30	(0.89,	1.91)	0.180	0.988
hsa-miR-361-3p	<-1.77	37	8.4	24	8.5	1.09	(0.63,	1.88)	0.748	0.977
	[–1.77, 2.08]	389	88.6	238	83.8	1.00				
	> 2.08	13	3.0	22	7.7	2.73	(1.34,	5.54)	0.006	0.988
hsa-miR-4520b-3p	<-1.77	103	23.5	57	20.1	0.77	(0.53,	1.12)	0.171	0.977
	[-1.77, 2.08]	294	67.0	211	74.3	1.00				
	> 2.08	42	9.6	16	5.6	0.51	(0.28,	0.95)	0.032	0.988
hsa-miR-4533	<-1.77	33	7.5	8	2.8	0.35	(0.16,	0.78)	0.010	0.977
	[–1.77, 2.08]	376	85.6	257	90.5	1.00				
	> 2.08	30	6.8	19	6.7	0.92	(0.50,	1.68)	0.783	1.000
hsa-miR-4731-3p	<-1.77	77	17.5	47	16.5	0.86	(0.57,	1.29)	0.462	0.977
	[–1.77, 2.08]	300	68.3	210	73.9	1.00				
	> 2.08	62	14.1	27	9.5	0.60	(0.37,	0.97)	0.039	0.988
hsa-miR-6515-5p	<-1.77	305	69.5	220	77.5	1.47	(1.02,	2.12)	0.040	0.977
	[–1.77, 2.08]	114	26.0	55	19.4	1.00				
	> 2.08	20	4.6	9	3.2	0.89	(0.38,	2.10)	0.795	1.000

Supplementary Table 3: Associations between infrequently expressed miRNAs and survival in rectal cancer cases. See Supplementary_Table_3

Supplementary Table 4: Infrequently expressed miRNAs and associations with survival adjusted for MSI along with age, center, AJCC stage, and sex. See Supplementary_Table_4
