# Supplementary material for: Infrequently expressed miRNAs influence survival after diagnosis with colorectal cancer
Source: Oncotarget. 2017 Aug 3;8(48):83845–59. doi: 10.18632/oncotarget.19863 (PMC5663559; doi:10.18632/oncotarget.19863)
Supplement: Supplementary file 2 [file oncotarget-08-83845-s002.docx]

| Supplemental Table 1. Description of infrequently expressed miRNAs in colon and rectal tissue | | | | | | | | |  |  |  |  |  |
| --- | --- | --- | --- | --- | --- | --- | --- | --- | --- | --- | --- | --- | --- |
|  | Colon | | | | | | Rectal | | | | | | |
|  | Tumor | | | Normal | | | Tumor | | | | Normal | | |
| miRNA | N | % | Mean | N | % | Mean | N | % | | Mean | N | % | Mean |
| hsa_let_7b_3p | 141 | 12.3 | 1.01 | 324 | 27.2 | 0.95 | 94 | 12.7 | | 0.77 | 243 | 32.0 | 1.19 |
| hsa_let_7d_3p | 126 | 10.9 | 0.99 | 260 | 21.8 | 0.76 | 70 | 9.4 | | 0.65 | 215 | 28.3 | 0.93 |
| hsa_let_7e_3p | 5 | 0.4 | 0.04 | 1 | 0.1 | 0.01 | 1 | 0.1 | | 0.01 | 1 | 0.1 | 0.03 |
| hsa_let_7f_1_3p | 27 | 2.3 | 0.07 | 57 | 4.8 | 0.04 | 6 | 0.8 | | 0.02 | 89 | 11.7 | 0.10 |
| hsa_let_7g_3p | 19 | 1.7 | 0.03 | 42 | 3.5 | 0.03 | 15 | 2.0 | | 0.02 | 143 | 18.8 | 0.06 |
| hsa_let_7i_3p | 1 | 0.1 | 0.00 | 1 | 0.1 | 0.00 | 0 | 0.0 | | 0.00 | 0 | 0.0 | 0.00 |
| hsa_miR_1 | 33 | 2.9 | 0.11 | 100 | 8.4 | 0.27 | 11 | 1.5 | | 0.04 | 146 | 19.2 | 0.24 |
| hsa_miR_101_3p | 15 | 1.3 | 0.03 | 11 | 0.9 | 0.03 | 3 | 0.4 | | 0.01 | 2 | 0.3 | 0.00 |
| hsa_miR_106b_3p | 22 | 1.9 | 0.03 | 1 | 0.1 | 0.00 | 26 | 3.5 | | 0.04 | 2 | 0.3 | 0.00 |
| hsa_miR_1203 | 461 | 40.1 | 1.16 | 767 | 64.3 | 1.96 | 354 | 47.6 | | 1.31 | 480 | 63.2 | 1.79 |
| hsa_miR_1207_3p | 203 | 17.6 | 0.41 | 422 | 35.4 | 0.67 | 64 | 8.6 | | 0.16 | 237 | 31.2 | 0.38 |
| hsa_miR_1224_3p | 28 | 2.4 | 0.05 | 30 | 2.5 | 0.05 | 31 | 4.2 | | 0.08 | 85 | 11.2 | 0.15 |
| hsa_miR_1226_3p | 13 | 1.1 | 0.04 | 18 | 1.5 | 0.04 | 6 | 0.8 | | 0.03 | 4 | 0.5 | 0.03 |
| hsa_miR_1227_3p | 65 | 5.6 | 0.26 | 96 | 8.1 | 0.19 | 37 | 5.0 | | 0.16 | 152 | 20.0 | 0.30 |
| hsa_miR_1236_3p | 8 | 0.7 | 0.03 | 7 | 0.6 | 0.01 | 3 | 0.4 | | 0.01 | 3 | 0.4 | 0.03 |
| hsa_miR_1243 | 237 | 20.6 | 0.87 | 475 | 39.8 | 1.27 | 87 | 11.7 | | 0.38 | 220 | 28.9 | 0.50 |
| hsa_miR_1244 | 319 | 27.7 | 0.92 | 60 | 5.0 | 0.07 | 187 | 25.2 | | 0.71 | 37 | 4.9 | 0.04 |
| hsa_miR_1248 | 14 | 1.2 | 0.02 | 4 | 0.3 | 0.01 | 3 | 0.4 | | 0.01 | 1 | 0.1 | 0.00 |
| hsa_miR_124_3p | 208 | 18.1 | 0.48 | 446 | 37.4 | 0.73 | 116 | 15.6 | | 0.35 | 251 | 33.0 | 0.56 |
| hsa_miR_1250 | 101 | 8.8 | 0.15 | 96 | 8.1 | 0.10 | 44 | 5.9 | | 0.10 | 100 | 13.2 | 0.06 |
| hsa_miR_1255b_5p | 33 | 2.9 | 0.05 | 49 | 4.1 | 0.06 | 16 | 2.2 | | 0.04 | 96 | 12.6 | 0.08 |
| hsa_miR_1258 | 311 | 27.0 | 1.20 | 583 | 48.9 | 1.99 | 202 | 27.2 | | 1.09 | 346 | 45.5 | 1.60 |
| hsa_miR_1266 | 443 | 38.5 | 1.54 | 521 | 43.7 | 1.30 | 273 | 36.7 | | 1.30 | 286 | 37.6 | 0.89 |
| hsa_miR_126_5p | 5 | 0.4 | 0.01 | 40 | 3.4 | 0.03 | 3 | 0.4 | | 0.00 | 3 | 0.4 | 0.01 |
| hsa_miR_1271_5p | 89 | 7.7 | 0.22 | 231 | 19.4 | 0.41 | 102 | 13.7 | | 0.37 | 228 | 30.0 | 0.47 |
| hsa_miR_1276 | 572 | 49.7 | 1.75 | 669 | 56.1 | 1.72 | 374 | 50.3 | | 1.55 | 410 | 53.9 | 1.43 |
| hsa_miR_127_3p | 158 | 13.7 | 0.55 | 124 | 10.4 | 0.20 | 169 | 22.7 | | 0.87 | 178 | 23.4 | 0.35 |
| hsa_miR_127_5p | 92 | 8.0 | 0.07 | 98 | 8.2 | 0.05 | 69 | 9.3 | | 0.08 | 129 | 17.0 | 0.04 |
| hsa_miR_128 | 71 | 6.2 | 0.23 | 13 | 1.1 | 0.03 | 72 | 9.7 | | 0.28 | 40 | 5.3 | 0.04 |
| hsa_miR_1286 | 1 | 0.1 | 0.00 | 3 | 0.3 | 0.00 | 3 | 0.4 | | 0.01 | 0 | 0.0 | 0.00 |
| hsa_miR_1292_3p | 3 | 0.3 | 0.02 | 5 | 0.4 | 0.01 | 0 | 0.0 | | 0.00 | 77 | 10.1 | 0.05 |
| hsa_miR_1292_5p | 70 | 6.1 | 0.11 | 100 | 8.4 | 0.11 | 23 | 3.1 | | 0.05 | 82 | 10.8 | 0.08 |
| hsa_miR_1295b_3p | 440 | 38.2 | 1.68 | 585 | 49.1 | 1.78 | 316 | 42.5 | | 1.74 | 363 | 47.8 | 1.60 |
| hsa_miR_1296 | 59 | 5.1 | 0.27 | 157 | 13.2 | 0.27 | 52 | 7.0 | | 0.30 | 154 | 20.3 | 0.44 |
| hsa_miR_129_1_3p | 3 | 0.3 | 0.02 | 2 | 0.2 | 0.01 | 1 | 0.1 | | 0.00 | 3 | 0.4 | 0.02 |
| hsa_miR_129_2_3p | 6 | 0.5 | 0.04 | 5 | 0.4 | 0.02 | 3 | 0.4 | | 0.03 | 8 | 1.1 | 0.06 |
| hsa_miR_1301 | 14 | 1.2 | 0.02 | 41 | 3.4 | 0.02 | 19 | 2.6 | | 0.04 | 79 | 10.4 | 0.03 |
| hsa_miR_1304_5p | 11 | 1.0 | 0.02 | 7 | 0.6 | 0.01 | 3 | 0.4 | | 0.01 | 31 | 4.1 | 0.01 |
| hsa_miR_1306_5p | 1 | 0.1 | 0.00 | 1 | 0.1 | 0.01 | 0 | 0.0 | | 0.00 | 0 | 0.0 | 0.00 |
| hsa_miR_130a_5p | 3 | 0.3 | 0.02 | 1 | 0.1 | 0.01 | 1 | 0.1 | | 0.00 | 0 | 0.0 | 0.00 |
| hsa_miR_130b_5p | 2 | 0.2 | 0.01 | 0 | 0.0 | 0.00 | 0 | 0.0 | | 0.00 | 2 | 0.3 | 0.02 |
| hsa_miR_1322 | 0 | 0.0 | 0.00 | 0 | 0.0 | 0.00 | 1 | 0.1 | | 0.01 | 1 | 0.1 | 0.00 |
| hsa_miR_132_3p | 91 | 7.9 | 0.29 | 141 | 11.8 | 0.25 | 78 | 10.5 | | 0.33 | 174 | 22.9 | 0.37 |
| hsa_miR_133a | 28 | 2.4 | 0.09 | 83 | 7.0 | 0.20 | 9 | 1.2 | | 0.03 | 154 | 20.3 | 0.27 |
| hsa_miR_133b | 177 | 15.4 | 1.18 | 683 | 57.3 | 3.42 | 144 | 19.4 | | 1.13 | 596 | 78.4 | 5.52 |
| hsa_miR_1343 | 16 | 1.4 | 0.04 | 29 | 2.4 | 0.03 | 0 | 0.0 | | 0.00 | 1 | 0.1 | 0.00 |
| hsa_miR_135b_5p | 269 | 23.4 | 1.86 | 11 | 0.9 | 0.04 | 170 | 22.9 | | 1.46 | 4 | 0.5 | 0.04 |
| hsa_miR_136_5p | 2 | 0.2 | 0.02 | 4 | 0.3 | 0.01 | 0 | 0.0 | | 0.00 | 0 | 0.0 | 0.00 |
| hsa_miR_138_5p | 1 | 0.1 | 0.00 | 15 | 1.3 | 0.01 | 1 | 0.1 | | 0.00 | 1 | 0.1 | 0.00 |
| hsa_miR_139_5p | 570 | 49.5 | 2.35 | 788 | 66.1 | 3.25 | 349 | 47.0 | | 1.99 | 516 | 67.9 | 2.97 |
| hsa_miR_140_5p | 5 | 0.4 | 0.01 | 7 | 0.6 | 0.01 | 4 | 0.5 | | 0.01 | 7 | 0.9 | 0.02 |
| hsa_miR_142_3p | 368 | 32.0 | 1.97 | 729 | 61.2 | 3.70 | 215 | 28.9 | | 1.25 | 389 | 51.2 | 2.18 |
| hsa_miR_142_5p | 2 | 0.2 | 0.00 | 25 | 2.1 | 0.06 | 0 | 0.0 | | 0.00 | 2 | 0.3 | 0.02 |
| hsa_miR_143_5p | 84 | 7.3 | 0.38 | 111 | 9.3 | 0.36 | 50 | 6.7 | | 0.24 | 137 | 18.0 | 0.36 |
| hsa_miR_145_3p | 22 | 1.9 | 0.08 | 69 | 5.8 | 0.13 | 11 | 1.5 | | 0.03 | 110 | 14.5 | 0.12 |
| hsa_miR_146b_5p | 480 | 41.7 | 2.18 | 502 | 42.1 | 1.28 | 371 | 49.9 | | 2.32 | 332 | 43.7 | 0.93 |
| hsa_miR_148b_3p | 68 | 5.9 | 0.19 | 10 | 0.8 | 0.03 | 55 | 7.4 | | 0.19 | 5 | 0.7 | 0.02 |
| hsa_miR_149_5p | 8 | 0.7 | 0.05 | 2 | 0.2 | 0.00 | 12 | 1.6 | | 0.04 | 85 | 11.2 | 0.14 |
| hsa_miR_151a_3p | 539 | 46.8 | 2.71 | 344 | 28.9 | 0.71 | 437 | 58.8 | | 3.09 | 307 | 40.4 | 0.74 |
| hsa_miR_152 | 49 | 4.3 | 0.11 | 52 | 4.4 | 0.05 | 42 | 5.7 | | 0.19 | 51 | 6.7 | 0.04 |
| hsa_miR_17_3p | 202 | 17.5 | 0.61 | 11 | 0.9 | 0.03 | 168 | 22.6 | | 0.68 | 6 | 0.8 | 0.03 |
| hsa_miR_181c_3p | 10 | 0.9 | 0.02 | 17 | 1.4 | 0.01 | 5 | 0.7 | | 0.01 | 50 | 6.6 | 0.02 |
| hsa_miR_181c_5p | 20 | 1.7 | 0.09 | 2 | 0.2 | 0.00 | 11 | 1.5 | | 0.04 | 2 | 0.3 | 0.01 |
| hsa_miR_182_5p | 98 | 8.5 | 0.18 | 2 | 0.2 | 0.00 | 64 | 8.6 | | 0.12 | 1 | 0.1 | 0.00 |
| hsa_miR_183_3p | 413 | 35.9 | 0.81 | 493 | 41.4 | 0.64 | 219 | 29.5 | | 0.54 | 280 | 36.8 | 0.45 |
| hsa_miR_183_5p | 252 | 21.9 | 1.06 | 13 | 1.1 | 0.01 | 172 | 23.1 | | 0.74 | 5 | 0.7 | 0.02 |
| hsa_miR_184 | 414 | 36.0 | 1.68 | 574 | 48.2 | 1.86 | 237 | 31.9 | | 1.25 | 305 | 40.1 | 1.23 |
| hsa_miR_185_5p | 130 | 11.3 | 0.44 | 44 | 3.7 | 0.10 | 110 | 14.8 | | 0.47 | 103 | 13.6 | 0.11 |
| hsa_miR_186_3p | 3 | 0.3 | 0.01 | 5 | 0.4 | 0.01 | 0 | 0.0 | | 0.00 | 0 | 0.0 | 0.00 |
| hsa_miR_186_5p | 43 | 3.7 | 0.09 | 69 | 5.8 | 0.09 | 36 | 4.8 | | 0.11 | 49 | 6.4 | 0.06 |
| hsa_miR_18a_5p | 91 | 7.9 | 0.42 | 6 | 0.5 | 0.02 | 73 | 9.8 | | 0.45 | 2 | 0.3 | 0.02 |
| hsa_miR_18b_3p | 3 | 0.3 | 0.02 | 38 | 3.2 | 0.03 | 1 | 0.1 | | 0.00 | 27 | 3.6 | 0.01 |
| hsa_miR_18b_5p | 37 | 3.2 | 0.07 | 3 | 0.3 | 0.01 | 19 | 2.6 | | 0.06 | 2 | 0.3 | 0.00 |
| hsa_miR_190b | 2 | 0.2 | 0.01 | 0 | 0.0 | 0.00 | 1 | 0.1 | | 0.00 | 1 | 0.1 | 0.00 |
| hsa_miR_1910 | 114 | 9.9 | 0.48 | 144 | 12.1 | 0.22 | 80 | 10.8 | | 0.49 | 158 | 20.8 | 0.36 |
| hsa_miR_1913 | 148 | 12.9 | 0.38 | 148 | 12.4 | 0.20 | 61 | 8.2 | | 0.19 | 113 | 14.9 | 0.13 |
| hsa_miR_1914_5p | 51 | 4.4 | 0.13 | 44 | 3.7 | 0.08 | 37 | 5.0 | | 0.10 | 181 | 23.8 | 0.25 |
| hsa_miR_1915_5p | 229 | 19.9 | 0.62 | 408 | 34.2 | 0.77 | 107 | 14.4 | | 0.31 | 222 | 29.2 | 0.45 |
| hsa_miR_191_5p | 1 | 0.1 | 0.00 | 2 | 0.2 | 0.01 | 0 | 0.0 | | 0.00 | 0 | 0.0 | 0.00 |
| hsa_miR_192_3p | 218 | 18.9 | 0.75 | 516 | 43.3 | 1.48 | 219 | 29.5 | | 1.19 | 415 | 54.6 | 1.67 |
| hsa_miR_193a_3p | 389 | 33.8 | 1.55 | 133 | 11.2 | 0.21 | 235 | 31.6 | | 1.32 | 129 | 17.0 | 0.17 |
| hsa_miR_195_3p | 427 | 37.1 | 0.72 | 495 | 41.5 | 0.60 | 306 | 41.2 | | 0.87 | 364 | 47.9 | 0.75 |
| hsa_miR_195_5p | 467 | 40.6 | 2.54 | 972 | 81.5 | 8.90 | 407 | 54.8 | | 2.89 | 663 | 87.2 | 9.69 |
| hsa_miR_196a_3p | 1 | 0.1 | 0.00 | 0 | 0.0 | 0.00 | 0 | 0.0 | | 0.00 | 1 | 0.1 | 0.01 |
| hsa_miR_196b_3p | 6 | 0.5 | 0.03 | 1 | 0.1 | 0.01 | 5 | 0.7 | | 0.02 | 0 | 0.0 | 0.00 |
| hsa_miR_1976 | 14 | 1.2 | 0.14 | 9 | 0.8 | 0.06 | 2 | 0.3 | | 0.03 | 74 | 9.7 | 0.16 |
| hsa_miR_199b_5p | 392 | 34.1 | 1.82 | 342 | 28.7 | 0.67 | 354 | 47.6 | | 2.38 | 284 | 37.4 | 0.70 |
| hsa_miR_19a_3p | 306 | 26.6 | 1.83 | 59 | 4.9 | 0.14 | 215 | 28.9 | | 1.63 | 14 | 1.8 | 0.09 |
| hsa_miR_19a_5p | 1 | 0.1 | 0.00 | 8 | 0.7 | 0.01 | 1 | 0.1 | | 0.01 | 1 | 0.1 | 0.00 |
| hsa_miR_203a | 745 | 64.7 | 8.59 | 448 | 37.6 | 1.71 | 574 | 77.3 | | 10.70 | 463 | 60.9 | 2.98 |
| hsa_miR_204_3p | 166 | 14.4 | 0.43 | 422 | 35.4 | 0.77 | 49 | 6.6 | | 0.15 | 190 | 25.0 | 0.28 |
| hsa_miR_204_5p | 51 | 4.4 | 0.42 | 60 | 5.0 | 0.16 | 34 | 4.6 | | 0.46 | 96 | 12.6 | 0.20 |
| hsa_miR_2052 | 1 | 0.1 | 0.00 | 0 | 0.0 | 0.00 | 0 | 0.0 | | 0.00 | 1 | 0.1 | 0.01 |
| hsa_miR_205_5p | 19 | 1.7 | 0.32 | 3 | 0.3 | 0.01 | 14 | 1.9 | | 0.19 | 6 | 0.8 | 0.35 |
| hsa_miR_20a_3p | 1 | 0.1 | 0.01 | 2 | 0.2 | 0.00 | 0 | 0.0 | | 0.00 | 1 | 0.1 | 0.01 |
| hsa_miR_20b_3p | 11 | 1.0 | 0.01 | 19 | 1.6 | 0.01 | 4 | 0.5 | | 0.00 | 51 | 6.7 | 0.02 |
| hsa_miR_2110 | 252 | 21.9 | 0.49 | 430 | 36.1 | 0.58 | 53 | 7.1 | | 0.13 | 183 | 24.1 | 0.23 |
| hsa_miR_2114_3p | 165 | 14.3 | 0.44 | 307 | 25.8 | 0.56 | 88 | 11.8 | | 0.37 | 208 | 27.4 | 0.43 |
| hsa_miR_2116_3p | 5 | 0.4 | 0.01 | 63 | 5.3 | 0.06 | 5 | 0.7 | | 0.02 | 127 | 16.7 | 0.10 |
| hsa_miR_2117 | 285 | 24.8 | 0.90 | 641 | 53.8 | 2.22 | 203 | 27.3 | | 0.96 | 400 | 52.6 | 1.90 |
| hsa_miR_211_5p | 5 | 0.4 | 0.07 | 0 | 0.0 | 0.00 | 0 | 0.0 | | 0.00 | 2 | 0.3 | 0.07 |
| hsa_miR_224_5p | 698 | 60.6 | 8.54 | 98 | 8.2 | 0.41 | 576 | 77.5 | | 9.84 | 95 | 12.5 | 0.31 |
| hsa_miR_2278 | 410 | 35.6 | 1.15 | 506 | 42.4 | 1.02 | 266 | 35.8 | | 1.12 | 375 | 49.3 | 1.22 |
| hsa_miR_22_5p | 1 | 0.1 | 0.00 | 5 | 0.4 | 0.02 | 2 | 0.3 | | 0.04 | 14 | 1.8 | 0.05 |
| hsa_miR_23b_5p | 2 | 0.2 | 0.00 | 2 | 0.2 | 0.00 | 1 | 0.1 | | 0.00 | 0 | 0.0 | 0.00 |
| hsa_miR_23c | 2 | 0.2 | 0.01 | 1 | 0.1 | 0.01 | 0 | 0.0 | | 0.00 | 2 | 0.3 | 0.02 |
| hsa_miR_25_5p | 1 | 0.1 | 0.01 | 1 | 0.1 | 0.00 | 1 | 0.1 | | 0.03 | 0 | 0.0 | 0.00 |
| hsa_miR_2681_5p | 0 | 0.0 | 0.00 | 1 | 0.1 | 0.00 | 1 | 0.1 | | 0.00 | 0 | 0.0 | 0.00 |
| hsa_miR_27a_5p | 1 | 0.1 | 0.00 | 1 | 0.1 | 0.00 | 0 | 0.0 | | 0.00 | 0 | 0.0 | 0.00 |
| hsa_miR_28_3p | 515 | 44.7 | 0.98 | 738 | 61.9 | 1.13 | 336 | 45.2 | | 0.87 | 449 | 59.1 | 0.94 |
| hsa_miR_28_5p | 211 | 18.3 | 0.66 | 400 | 33.6 | 0.84 | 186 | 25.0 | | 0.77 | 345 | 45.4 | 0.99 |
| hsa_miR_296_3p | 3 | 0.3 | 0.00 | 5 | 0.4 | 0.00 | 0 | 0.0 | | 0.00 | 0 | 0.0 | 0.00 |
| hsa_miR_297 | 7 | 0.6 | 0.01 | 21 | 1.8 | 0.02 | 11 | 1.5 | | 0.02 | 3 | 0.4 | 0.00 |
| hsa_miR_299_3p | 258 | 22.4 | 0.82 | 382 | 32.0 | 1.09 | 124 | 16.7 | | 0.52 | 257 | 33.8 | 0.81 |
| hsa_miR_299_5p | 22 | 1.9 | 0.22 | 58 | 4.9 | 0.24 | 9 | 1.2 | | 0.18 | 76 | 10.0 | 0.20 |
| hsa_miR_29b_1_5p | 27 | 2.3 | 0.04 | 6 | 0.5 | 0.01 | 19 | 2.6 | | 0.03 | 2 | 0.3 | 0.00 |
| hsa_miR_29c_5p | 1 | 0.1 | 0.00 | 24 | 2.0 | 0.03 | 1 | 0.1 | | 0.00 | 39 | 5.1 | 0.03 |
| hsa_miR_300 | 9 | 0.8 | 0.02 | 6 | 0.5 | 0.01 | 1 | 0.1 | | 0.00 | 3 | 0.4 | 0.01 |
| hsa_miR_301b | 1 | 0.1 | 0.00 | 0 | 0.0 | 0.00 | 0 | 0.0 | | 0.00 | 29 | 3.8 | 0.01 |
| hsa_miR_3064_5p | 34 | 3.0 | 0.06 | 124 | 10.4 | 0.15 | 16 | 2.2 | | 0.05 | 102 | 13.4 | 0.08 |
| hsa_miR_3065_3p | 7 | 0.6 | 0.01 | 22 | 1.8 | 0.05 | 3 | 0.4 | | 0.01 | 4 | 0.5 | 0.01 |
| hsa_miR_3065_5p | 6 | 0.5 | 0.01 | 8 | 0.7 | 0.01 | 1 | 0.1 | | 0.00 | 2 | 0.3 | 0.01 |
| hsa_miR_3074_3p | 5 | 0.4 | 0.01 | 11 | 0.9 | 0.01 | 3 | 0.4 | | 0.01 | 1 | 0.1 | 0.00 |
| hsa_miR_3074_5p | 0 | 0.0 | 0.00 | 0 | 0.0 | 0.00 | 2 | 0.3 | | 0.01 | 2 | 0.3 | 0.05 |
| hsa_miR_30a_5p | 339 | 29.5 | 1.33 | 690 | 57.9 | 2.68 | 248 | 33.4 | | 1.12 | 460 | 60.5 | 2.51 |
| hsa_miR_30e_3p | 1 | 0.1 | 0.01 | 6 | 0.5 | 0.01 | 2 | 0.3 | | 0.00 | 4 | 0.5 | 0.01 |
| hsa_miR_30e_5p | 506 | 44.0 | 2.12 | 775 | 65.0 | 3.04 | 379 | 51.0 | | 2.07 | 502 | 66.1 | 2.44 |
| hsa_miR_3116 | 2 | 0.2 | 0.00 | 12 | 1.0 | 0.03 | 1 | 0.1 | | 0.00 | 5 | 0.7 | 0.02 |
| hsa_miR_3120_3p | 57 | 5.0 | 0.10 | 75 | 6.3 | 0.09 | 33 | 4.4 | | 0.09 | 89 | 11.7 | 0.07 |
| hsa_miR_3122 | 403 | 35.0 | 1.34 | 522 | 43.8 | 1.45 | 272 | 36.6 | | 1.23 | 324 | 42.6 | 1.13 |
| hsa_miR_3124_5p | 399 | 34.7 | 1.03 | 624 | 52.3 | 1.45 | 254 | 34.2 | | 0.93 | 350 | 46.1 | 1.01 |
| hsa_miR_3126_3p | 0 | 0.0 | 0.00 | 4 | 0.3 | 0.01 | 1 | 0.1 | | 0.01 | 1 | 0.1 | 0.00 |
| hsa_miR_3130_3p | 236 | 20.5 | 0.65 | 355 | 29.8 | 0.67 | 105 | 14.1 | | 0.39 | 167 | 22.0 | 0.38 |
| hsa_miR_3135a | 33 | 2.9 | 0.04 | 33 | 2.8 | 0.03 | 23 | 3.1 | | 0.04 | 81 | 10.7 | 0.03 |
| hsa_miR_3148 | 34 | 3.0 | 0.14 | 116 | 9.7 | 0.19 | 47 | 6.3 | | 0.30 | 16 | 2.1 | 0.14 |
| hsa_miR_3149 | 513 | 44.6 | 4.08 | 561 | 47.1 | 3.22 | 352 | 47.4 | | 4.43 | 459 | 60.4 | 4.20 |
| hsa_miR_3150b_3p | 456 | 39.6 | 1.62 | 564 | 47.3 | 1.54 | 274 | 36.9 | | 1.32 | 329 | 43.3 | 0.99 |
| hsa_miR_3151 | 174 | 15.1 | 0.29 | 309 | 25.9 | 0.37 | 82 | 11.0 | | 0.20 | 203 | 26.7 | 0.25 |
| hsa_miR_3152_3p | 71 | 6.2 | 0.26 | 186 | 15.6 | 0.31 | 42 | 5.7 | | 0.24 | 169 | 22.2 | 0.37 |
| hsa_miR_3155a | 8 | 0.7 | 0.01 | 11 | 0.9 | 0.01 | 8 | 1.1 | | 0.01 | 4 | 0.5 | 0.01 |
| hsa_miR_3159 | 14 | 1.2 | 0.02 | 39 | 3.3 | 0.06 | 6 | 0.8 | | 0.01 | 20 | 2.6 | 0.03 |
| hsa_miR_3164 | 355 | 30.8 | 1.25 | 569 | 47.7 | 1.76 | 142 | 19.1 | | 0.60 | 297 | 39.1 | 0.95 |
| hsa_miR_3170 | 266 | 23.1 | 0.95 | 454 | 38.1 | 1.16 | 179 | 24.1 | | 0.94 | 288 | 37.9 | 1.11 |
| hsa_miR_3178 | 310 | 26.9 | 0.67 | 544 | 45.6 | 1.00 | 170 | 22.9 | | 0.50 | 310 | 40.8 | 0.82 |
| hsa_miR_3180 | 74 | 6.4 | 0.15 | 30 | 2.5 | 0.03 | 63 | 8.5 | | 0.16 | 33 | 4.3 | 0.03 |
| hsa_miR_3180_5p | 473 | 41.1 | 1.55 | 598 | 50.2 | 1.53 | 333 | 44.8 | | 1.55 | 400 | 52.6 | 1.66 |
| hsa_miR_3181 | 350 | 30.4 | 1.46 | 600 | 50.3 | 2.19 | 163 | 21.9 | | 0.82 | 340 | 44.7 | 1.54 |
| hsa_miR_3184_3p | 22 | 1.9 | 0.05 | 75 | 6.3 | 0.05 | 29 | 3.9 | | 0.11 | 57 | 7.5 | 0.07 |
| hsa_miR_3186_3p | 371 | 32.2 | 1.27 | 497 | 41.7 | 1.29 | 227 | 30.6 | | 1.04 | 265 | 34.9 | 0.85 |
| hsa_miR_3190_5p | 15 | 1.3 | 0.11 | 23 | 1.9 | 0.12 | 7 | 0.9 | | 0.09 | 110 | 14.5 | 0.15 |
| hsa_miR_3191_3p | 127 | 11.0 | 0.32 | 290 | 24.3 | 0.42 | 69 | 9.3 | | 0.23 | 200 | 26.3 | 0.28 |
| hsa_miR_3192 | 4 | 0.3 | 0.02 | 21 | 1.8 | 0.18 | 4 | 0.5 | | 0.03 | 7 | 0.9 | 0.11 |
| hsa_miR_3193 | 69 | 6.0 | 0.15 | 75 | 6.3 | 0.12 | 22 | 3.0 | | 0.06 | 85 | 11.2 | 0.10 |
| hsa_miR_3194_3p | 17 | 1.5 | 0.02 | 120 | 10.1 | 0.07 | 3 | 0.4 | | 0.01 | 3 | 0.4 | 0.01 |
| hsa_miR_31_5p | 198 | 17.2 | 2.24 | 33 | 2.8 | 0.16 | 82 | 11.0 | | 0.69 | 3 | 0.4 | 0.02 |
| hsa_miR_323a_5p | 9 | 0.8 | 0.01 | 5 | 0.4 | 0.00 | 0 | 0.0 | | 0.00 | 0 | 0.0 | 0.00 |
| hsa_miR_324_5p | 427 | 37.1 | 2.33 | 348 | 29.2 | 0.99 | 325 | 43.7 | | 2.57 | 309 | 40.7 | 1.10 |
| hsa_miR_328 | 168 | 14.6 | 1.25 | 347 | 29.1 | 1.12 | 151 | 20.3 | | 1.19 | 271 | 35.7 | 1.61 |
| hsa_miR_32_3p | 450 | 39.1 | 2.62 | 479 | 40.2 | 1.98 | 299 | 40.2 | | 2.71 | 383 | 50.4 | 2.50 |
| hsa_miR_331_5p | 4 | 0.3 | 0.01 | 3 | 0.3 | 0.00 | 0 | 0.0 | | 0.00 | 2 | 0.3 | 0.01 |
| hsa_miR_335_3p | 6 | 0.5 | 0.03 | 5 | 0.4 | 0.01 | 5 | 0.7 | | 0.01 | 2 | 0.3 | 0.01 |
| hsa_miR_335_5p | 72 | 6.3 | 0.13 | 85 | 7.1 | 0.11 | 45 | 6.1 | | 0.12 | 110 | 14.5 | 0.12 |
| hsa_miR_337_3p | 10 | 0.9 | 0.10 | 7 | 0.6 | 0.05 | 5 | 0.7 | | 0.06 | 25 | 3.3 | 0.07 |
| hsa_miR_338_3p | 35 | 3.0 | 0.12 | 81 | 6.8 | 0.15 | 23 | 3.1 | | 0.08 | 86 | 11.3 | 0.17 |
| hsa_miR_339_5p | 91 | 7.9 | 0.10 | 21 | 1.8 | 0.02 | 76 | 10.2 | | 0.14 | 102 | 13.4 | 0.03 |
| hsa_miR_342_5p | 0 | 0.0 | 0.00 | 8 | 0.7 | 0.02 | 1 | 0.1 | | 0.00 | 2 | 0.3 | 0.01 |
| hsa_miR_346 | 3 | 0.3 | 0.01 | 3 | 0.3 | 0.00 | 0 | 0.0 | | 0.00 | 0 | 0.0 | 0.00 |
| hsa_miR_34b_3p | 3 | 0.3 | 0.03 | 2 | 0.2 | 0.02 | 5 | 0.7 | | 0.06 | 49 | 6.4 | 0.12 |
| hsa_miR_34b_5p | 22 | 1.9 | 0.07 | 4 | 0.3 | 0.01 | 11 | 1.5 | | 0.05 | 3 | 0.4 | 0.01 |
| hsa_miR_3591_3p | 124 | 10.8 | 0.53 | 286 | 24.0 | 0.49 | 149 | 20.1 | | 1.06 | 258 | 33.9 | 0.85 |
| hsa_miR_3606_3p | 19 | 1.7 | 0.07 | 125 | 10.5 | 0.08 | 0 | 0.0 | | 0.00 | 0 | 0.0 | 0.00 |
| hsa_miR_3607_3p | 172 | 14.9 | 0.55 | 343 | 28.8 | 0.48 | 117 | 15.7 | | 0.47 | 244 | 32.1 | 0.42 |
| hsa_miR_3607_5p | 90 | 7.8 | 0.30 | 291 | 24.4 | 0.48 | 45 | 6.1 | | 0.17 | 153 | 20.1 | 0.22 |
| hsa_miR_3609 | 488 | 42.4 | 1.59 | 604 | 50.7 | 1.30 | 369 | 49.7 | | 1.79 | 387 | 50.9 | 1.28 |
| hsa_miR_3613_3p | 14 | 1.2 | 0.16 | 65 | 5.5 | 0.17 | 16 | 2.2 | | 0.13 | 96 | 12.6 | 0.35 |
| hsa_miR_3615 | 416 | 36.1 | 1.56 | 580 | 48.7 | 2.06 | 93 | 12.5 | | 0.37 | 247 | 32.5 | 0.69 |
| hsa_miR_3616_3p | 555 | 48.2 | 2.56 | 625 | 52.4 | 2.23 | 384 | 51.7 | | 2.39 | 383 | 50.4 | 1.87 |
| hsa_miR_3617_3p | 46 | 4.0 | 0.53 | 84 | 7.0 | 0.44 | 18 | 2.4 | | 0.30 | 134 | 17.6 | 0.49 |
| hsa_miR_3619_5p | 88 | 7.6 | 0.25 | 205 | 17.2 | 0.35 | 29 | 3.9 | | 0.09 | 113 | 14.9 | 0.16 |
| hsa_miR_361_3p | 121 | 10.5 | 0.32 | 219 | 18.4 | 0.39 | 80 | 10.8 | | 0.29 | 260 | 34.2 | 0.46 |
| hsa_miR_3622a_3p | 5 | 0.4 | 0.04 | 2 | 0.2 | 0.01 | 0 | 0.0 | | 0.00 | 2 | 0.3 | 0.05 |
| hsa_miR_3622b_3p | 424 | 36.8 | 1.30 | 221 | 18.5 | 0.36 | 231 | 31.1 | | 0.97 | 173 | 22.8 | 0.45 |
| hsa_miR_362_5p | 348 | 30.2 | 1.26 | 133 | 11.2 | 0.20 | 285 | 38.4 | | 1.45 | 167 | 22.0 | 0.24 |
| hsa_miR_363_3p | 4 | 0.3 | 0.04 | 4 | 0.3 | 0.02 | 2 | 0.3 | | 0.02 | 0 | 0.0 | 0.00 |
| hsa_miR_3655 | 6 | 0.5 | 0.01 | 8 | 0.7 | 0.01 | 1 | 0.1 | | 0.00 | 0 | 0.0 | 0.00 |
| hsa_miR_3661 | 0 | 0.0 | 0.00 | 4 | 0.3 | 0.00 | 1 | 0.1 | | 0.00 | 0 | 0.0 | 0.00 |
| hsa_miR_3675_3p | 34 | 3.0 | 0.07 | 63 | 5.3 | 0.09 | 16 | 2.2 | | 0.05 | 72 | 9.5 | 0.06 |
| hsa_miR_3678_3p | 536 | 46.6 | 2.71 | 672 | 56.4 | 2.94 | 364 | 49.0 | | 2.53 | 403 | 53.0 | 2.23 |
| hsa_miR_3679_3p | 243 | 21.1 | 0.59 | 383 | 32.1 | 0.56 | 114 | 15.3 | | 0.37 | 235 | 30.9 | 0.44 |
| hsa_miR_367_3p | 1 | 0.1 | 0.00 | 1 | 0.1 | 0.00 | 0 | 0.0 | | 0.00 | 0 | 0.0 | 0.00 |
| hsa_miR_3685 | 5 | 0.4 | 0.02 | 3 | 0.3 | 0.02 | 2 | 0.3 | | 0.02 | 3 | 0.4 | 0.03 |
| hsa_miR_3687 | 331 | 28.8 | 1.10 | 77 | 6.5 | 0.14 | 309 | 41.6 | | 1.49 | 130 | 17.1 | 0.19 |
| hsa_miR_3714 | 8 | 0.7 | 0.05 | 4 | 0.3 | 0.02 | 1 | 0.1 | | 0.01 | 2 | 0.3 | 0.03 |
| hsa_miR_371a_3p | 1 | 0.1 | 0.01 | 2 | 0.2 | 0.02 | 2 | 0.3 | | 0.12 | 30 | 3.9 | 0.04 |
| hsa_miR_372 | 0 | 0.0 | 0.00 | 0 | 0.0 | 0.00 | 2 | 0.3 | | 0.20 | 29 | 3.8 | 0.04 |
| hsa_miR_373_3p | 19 | 1.7 | 0.06 | 66 | 5.5 | 0.04 | 3 | 0.4 | | 0.35 | 31 | 4.1 | 0.12 |
| hsa_miR_374a_3p | 1 | 0.1 | 0.00 | 9 | 0.8 | 0.00 | 2 | 0.3 | | 0.01 | 2 | 0.3 | 0.01 |
| hsa_miR_374a_5p | 225 | 19.5 | 1.33 | 65 | 5.5 | 0.16 | 171 | 23.0 | | 1.45 | 18 | 2.4 | 0.11 |
| hsa_miR_374b_5p | 300 | 26.1 | 2.00 | 99 | 8.3 | 0.35 | 265 | 35.7 | | 2.52 | 126 | 16.6 | 0.27 |
| hsa_miR_376a_3p | 6 | 0.5 | 0.02 | 3 | 0.3 | 0.01 | 3 | 0.4 | | 0.01 | 4 | 0.5 | 0.01 |
| hsa_miR_376c_3p | 6 | 0.5 | 0.02 | 2 | 0.2 | 0.01 | 2 | 0.3 | | 0.01 | 3 | 0.4 | 0.01 |
| hsa_miR_378a_5p | 1 | 0.1 | 0.00 | 24 | 2.0 | 0.09 | 1 | 0.1 | | 0.00 | 45 | 5.9 | 0.07 |
| hsa_miR_378d | 51 | 4.4 | 0.09 | 377 | 31.6 | 0.59 | 30 | 4.0 | | 0.08 | 295 | 38.8 | 0.62 |
| hsa_miR_378e | 508 | 44.1 | 1.35 | 632 | 53.0 | 1.38 | 366 | 49.3 | | 1.39 | 393 | 51.7 | 1.19 |
| hsa_miR_378g | 263 | 22.8 | 0.67 | 474 | 39.8 | 1.02 | 183 | 24.6 | | 0.66 | 325 | 42.8 | 0.98 |
| hsa_miR_378h | 76 | 6.6 | 0.17 | 181 | 15.2 | 0.20 | 40 | 5.4 | | 0.15 | 30 | 3.9 | 0.06 |
| hsa_miR_378j | 199 | 17.3 | 0.50 | 312 | 26.2 | 0.51 | 119 | 16.0 | | 0.45 | 206 | 27.1 | 0.40 |
| hsa_miR_379_3p | 2 | 0.2 | 0.01 | 1 | 0.1 | 0.00 | 0 | 0.0 | | 0.00 | 3 | 0.4 | 0.01 |
| hsa_miR_379_5p | 1 | 0.1 | 0.00 | 2 | 0.2 | 0.01 | 0 | 0.0 | | 0.00 | 0 | 0.0 | 0.00 |
| hsa_miR_380_5p | 1 | 0.1 | 0.00 | 8 | 0.7 | 0.00 | 0 | 0.0 | | 0.00 | 0 | 0.0 | 0.00 |
| hsa_miR_381_3p | 81 | 7.0 | 0.12 | 184 | 15.4 | 0.17 | 39 | 5.2 | | 0.09 | 152 | 20.0 | 0.14 |
| hsa_miR_382_5p | 10 | 0.9 | 0.02 | 2 | 0.2 | 0.00 | 14 | 1.9 | | 0.04 | 2 | 0.3 | 0.00 |
| hsa_miR_3908 | 14 | 1.2 | 0.02 | 157 | 13.2 | 0.13 | 5 | 0.7 | | 0.01 | 72 | 9.5 | 0.07 |
| hsa_miR_3915 | 4 | 0.3 | 0.03 | 2 | 0.2 | 0.00 | 2 | 0.3 | | 0.01 | 1 | 0.1 | 0.00 |
| hsa_miR_3918 | 130 | 11.3 | 0.24 | 210 | 17.6 | 0.28 | 82 | 11.0 | | 0.23 | 192 | 25.3 | 0.26 |
| hsa_miR_3922_5p | 343 | 29.8 | 0.78 | 426 | 35.7 | 0.67 | 232 | 31.2 | | 0.77 | 268 | 35.3 | 0.66 |
| hsa_miR_3923 | 95 | 8.3 | 0.19 | 298 | 25.0 | 0.41 | 21 | 2.8 | | 0.04 | 105 | 13.8 | 0.15 |
| hsa_miR_3927_5p | 7 | 0.6 | 0.06 | 2 | 0.2 | 0.01 | 3 | 0.4 | | 0.02 | 2 | 0.3 | 0.02 |
| hsa_miR_3936 | 0 | 0.0 | 0.00 | 2 | 0.2 | 0.00 | 3 | 0.4 | | 0.01 | 0 | 0.0 | 0.00 |
| hsa_miR_3938 | 72 | 6.3 | 0.23 | 255 | 21.4 | 0.32 | 22 | 3.0 | | 0.08 | 57 | 7.5 | 0.11 |
| hsa_miR_3939 | 16 | 1.4 | 0.02 | 19 | 1.6 | 0.02 | 13 | 1.7 | | 0.03 | 45 | 5.9 | 0.04 |
| hsa_miR_3940_3p | 84 | 7.3 | 0.75 | 169 | 14.2 | 0.72 | 52 | 7.0 | | 0.61 | 202 | 26.6 | 0.93 |
| hsa_miR_3944_5p | 483 | 42.0 | 2.09 | 643 | 53.9 | 2.29 | 315 | 42.4 | | 2.13 | 399 | 52.5 | 2.14 |
| hsa_miR_3972 | 586 | 50.9 | 2.73 | 609 | 51.1 | 1.76 | 314 | 42.3 | | 2.34 | 315 | 41.4 | 1.16 |
| hsa_miR_3976 | 303 | 26.3 | 0.91 | 308 | 25.8 | 0.46 | 267 | 35.9 | | 1.24 | 224 | 29.5 | 0.50 |
| hsa_miR_3978 | 7 | 0.6 | 0.01 | 12 | 1.0 | 0.01 | 1 | 0.1 | | 0.00 | 1 | 0.1 | 0.00 |
| hsa_miR_409_3p | 6 | 0.5 | 0.02 | 4 | 0.3 | 0.01 | 14 | 1.9 | | 0.06 | 1 | 0.1 | 0.00 |
| hsa_miR_424_5p | 354 | 30.8 | 1.48 | 126 | 10.6 | 0.21 | 316 | 42.5 | | 1.81 | 150 | 19.7 | 0.23 |
| hsa_miR_4251 | 413 | 35.9 | 1.68 | 389 | 32.6 | 0.85 | 329 | 44.3 | | 1.94 | 242 | 31.8 | 0.71 |
| hsa_miR_4252 | 12 | 1.0 | 0.01 | 45 | 3.8 | 0.03 | 5 | 0.7 | | 0.01 | 92 | 12.1 | 0.05 |
| hsa_miR_4254 | 5 | 0.4 | 0.02 | 3 | 0.3 | 0.01 | 15 | 2.0 | | 0.11 | 52 | 6.8 | 0.13 |
| hsa_miR_4255 | 96 | 8.3 | 0.22 | 197 | 16.5 | 0.31 | 34 | 4.6 | | 0.11 | 165 | 21.7 | 0.15 |
| hsa_miR_4256 | 18 | 1.6 | 0.03 | 150 | 12.6 | 0.10 | 2 | 0.3 | | 0.00 | 3 | 0.4 | 0.01 |
| hsa_miR_4258 | 3 | 0.3 | 0.00 | 12 | 1.0 | 0.01 | 2 | 0.3 | | 0.00 | 29 | 3.8 | 0.01 |
| hsa_miR_4267 | 50 | 4.3 | 0.09 | 99 | 8.3 | 0.09 | 11 | 1.5 | | 0.03 | 57 | 7.5 | 0.03 |
| hsa_miR_4274 | 134 | 11.6 | 0.47 | 240 | 20.1 | 0.42 | 73 | 9.8 | | 0.29 | 190 | 25.0 | 0.44 |
| hsa_miR_4279 | 14 | 1.2 | 0.03 | 130 | 10.9 | 0.10 | 7 | 0.9 | | 0.02 | 91 | 12.0 | 0.10 |
| hsa_miR_4282 | 546 | 47.4 | 2.22 | 710 | 59.6 | 2.68 | 330 | 44.4 | | 1.83 | 418 | 55.0 | 2.32 |
| hsa_miR_4289 | 18 | 1.6 | 0.03 | 44 | 3.7 | 0.04 | 5 | 0.7 | | 0.01 | 81 | 10.7 | 0.04 |
| hsa_miR_4290 | 180 | 15.6 | 1.27 | 296 | 24.8 | 1.09 | 189 | 25.4 | | 1.85 | 308 | 40.5 | 2.51 |
| hsa_miR_4292 | 3 | 0.3 | 0.03 | 1 | 0.1 | 0.00 | 0 | 0.0 | | 0.00 | 20 | 2.6 | 0.02 |
| hsa_miR_4293 | 0 | 0.0 | 0.00 | 1 | 0.1 | 0.00 | 2 | 0.3 | | 0.01 | 2 | 0.3 | 0.01 |
| hsa_miR_4296 | 407 | 35.4 | 1.04 | 455 | 38.2 | 0.72 | 292 | 39.3 | | 1.01 | 261 | 34.3 | 0.61 |
| hsa_miR_4297 | 2 | 0.2 | 0.02 | 1 | 0.1 | 0.03 | 0 | 0.0 | | 0.00 | 0 | 0.0 | 0.00 |
| hsa_miR_4300 | 466 | 40.5 | 1.07 | 544 | 45.6 | 0.98 | 333 | 44.8 | | 1.13 | 363 | 47.8 | 0.94 |
| hsa_miR_4302 | 1 | 0.1 | 0.00 | 4 | 0.3 | 0.01 | 1 | 0.1 | | 0.00 | 0 | 0.0 | 0.00 |
| hsa_miR_4303 | 381 | 33.1 | 1.37 | 560 | 47.0 | 1.69 | 248 | 33.4 | | 1.24 | 345 | 45.4 | 1.40 |
| hsa_miR_4310 | 1 | 0.1 | 0.00 | 0 | 0.0 | 0.00 | 0 | 0.0 | | 0.00 | 1 | 0.1 | 0.01 |
| hsa_miR_4312 | 192 | 16.7 | 1.07 | 430 | 36.1 | 1.41 | 143 | 19.2 | | 1.08 | 304 | 40.0 | 1.74 |
| hsa_miR_4315 | 18 | 1.6 | 0.04 | 278 | 23.3 | 0.54 | 4 | 0.5 | | 0.01 | 176 | 23.2 | 0.29 |
| hsa_miR_4317 | 352 | 30.6 | 0.96 | 297 | 24.9 | 0.51 | 259 | 34.9 | | 0.95 | 176 | 23.2 | 0.41 |
| hsa_miR_4319 | 18 | 1.6 | 0.41 | 54 | 4.5 | 0.62 | 10 | 1.3 | | 0.02 | 93 | 12.2 | 0.07 |
| hsa_miR_4321 | 17 | 1.5 | 0.02 | 61 | 5.1 | 0.04 | 3 | 0.4 | | 0.01 | 30 | 3.9 | 0.01 |
| hsa_miR_4324 | 428 | 37.2 | 1.25 | 573 | 48.1 | 0.99 | 411 | 55.3 | | 1.84 | 508 | 66.8 | 1.82 |
| hsa_miR_4326 | 24 | 2.1 | 0.04 | 93 | 7.8 | 0.06 | 11 | 1.5 | | 0.02 | 89 | 11.7 | 0.10 |
| hsa_miR_432_5p | 579 | 50.3 | 2.25 | 666 | 55.9 | 2.18 | 365 | 49.1 | | 1.75 | 384 | 50.5 | 1.46 |
| hsa_miR_433 | 8 | 0.7 | 0.01 | 1 | 0.1 | 0.01 | 3 | 0.4 | | 0.01 | 1 | 0.1 | 0.00 |
| hsa_miR_4330 | 11 | 1.0 | 0.01 | 37 | 3.1 | 0.02 | 0 | 0.0 | | 0.00 | 80 | 10.5 | 0.04 |
| hsa_miR_4421 | 261 | 22.7 | 0.76 | 495 | 41.5 | 1.39 | 194 | 26.1 | | 0.81 | 314 | 41.3 | 1.03 |
| hsa_miR_4422 | 16 | 1.4 | 0.14 | 2 | 0.2 | 0.02 | 6 | 0.8 | | 0.02 | 2 | 0.3 | 0.01 |
| hsa_miR_4426 | 22 | 1.9 | 0.02 | 4 | 0.3 | 0.00 | 12 | 1.6 | | 0.02 | 31 | 4.1 | 0.01 |
| hsa_miR_4427 | 26 | 2.3 | 0.11 | 8 | 0.7 | 0.02 | 14 | 1.9 | | 0.09 | 3 | 0.4 | 0.01 |
| hsa_miR_4435 | 0 | 0.0 | 0.00 | 3 | 0.3 | 0.00 | 1 | 0.1 | | 0.00 | 0 | 0.0 | 0.00 |
| hsa_miR_4440 | 230 | 20.0 | 0.84 | 378 | 31.7 | 0.99 | 196 | 26.4 | | 1.29 | 329 | 43.3 | 1.32 |
| hsa_miR_4446_5p | 1 | 0.1 | 0.01 | 1 | 0.1 | 0.02 | 0 | 0.0 | | 0.00 | 48 | 6.3 | 0.25 |
| hsa_miR_4456 | 2 | 0.2 | 0.02 | 1 | 0.1 | 0.01 | 1 | 0.1 | | 0.01 | 0 | 0.0 | 0.00 |
| hsa_miR_4469 | 279 | 24.2 | 0.77 | 543 | 45.6 | 1.20 | 189 | 25.4 | | 0.72 | 339 | 44.6 | 1.13 |
| hsa_miR_4474_3p | 29 | 2.5 | 0.04 | 32 | 2.7 | 0.01 | 25 | 3.4 | | 0.05 | 31 | 4.1 | 0.01 |
| hsa_miR_4479 | 348 | 30.2 | 1.14 | 524 | 44.0 | 1.32 | 234 | 31.5 | | 1.11 | 337 | 44.3 | 1.20 |
| hsa_miR_4480 | 4 | 0.3 | 0.01 | 4 | 0.3 | 0.01 | 1 | 0.1 | | 0.00 | 1 | 0.1 | 0.00 |
| hsa_miR_4482_5p | 0 | 0.0 | 0.00 | 1 | 0.1 | 0.00 | 1 | 0.1 | | 0.00 | 0 | 0.0 | 0.00 |
| hsa_miR_4483 | 8 | 0.7 | 0.03 | 1 | 0.1 | 0.00 | 0 | 0.0 | | 0.00 | 0 | 0.0 | 0.00 |
| hsa_miR_4492 | 154 | 13.4 | 0.44 | 327 | 27.4 | 0.55 | 78 | 10.5 | | 0.26 | 197 | 25.9 | 0.39 |
| hsa_miR_449b_3p | 72 | 6.3 | 0.74 | 172 | 14.4 | 0.69 | 38 | 5.1 | | 0.52 | 180 | 23.7 | 0.87 |
| hsa_miR_449c_3p | 3 | 0.3 | 0.02 | 2 | 0.2 | 0.00 | 0 | 0.0 | | 0.00 | 0 | 0.0 | 0.00 |
| hsa_miR_4512 | 3 | 0.3 | 0.03 | 4 | 0.3 | 0.03 | 2 | 0.3 | | 0.03 | 3 | 0.4 | 0.04 |
| hsa_miR_4518 | 346 | 30.1 | 1.17 | 549 | 46.1 | 1.42 | 241 | 32.4 | | 1.32 | 360 | 47.4 | 1.51 |
| hsa_miR_451b | 23 | 2.0 | 0.21 | 42 | 3.5 | 0.16 | 41 | 5.5 | | 0.33 | 70 | 9.2 | 0.73 |
| hsa_miR_4520a_3p | 4 | 0.3 | 0.02 | 1 | 0.1 | 0.00 | 0 | 0.0 | | 0.00 | 0 | 0.0 | 0.00 |
| hsa_miR_4520b_3p | 292 | 25.4 | 0.98 | 522 | 43.8 | 1.46 | 176 | 23.7 | | 0.74 | 299 | 39.3 | 1.09 |
| hsa_miR_452_3p | 0 | 0.0 | 0.00 | 0 | 0.0 | 0.00 | 1 | 0.1 | | 0.00 | 1 | 0.1 | 0.00 |
| hsa_miR_4533 | 279 | 24.2 | 1.12 | 437 | 36.7 | 1.34 | 119 | 16.0 | | 0.55 | 232 | 30.5 | 0.79 |
| hsa_miR_455_3p | 159 | 13.8 | 0.69 | 10 | 0.8 | 0.03 | 193 | 26.0 | | 1.16 | 3 | 0.4 | 0.02 |
| hsa_miR_4638_3p | 140 | 12.2 | 0.36 | 275 | 23.1 | 0.30 | 58 | 7.8 | | 0.15 | 153 | 20.1 | 0.16 |
| hsa_miR_4638_5p | 569 | 49.4 | 3.54 | 710 | 59.6 | 4.37 | 341 | 45.9 | | 2.56 | 411 | 54.1 | 2.79 |
| hsa_miR_4642 | 109 | 9.5 | 0.28 | 165 | 13.8 | 0.21 | 41 | 5.5 | | 0.15 | 130 | 17.1 | 0.13 |
| hsa_miR_4646_3p | 156 | 13.6 | 0.59 | 284 | 23.8 | 0.52 | 158 | 21.3 | | 0.90 | 268 | 35.3 | 1.16 |
| hsa_miR_4649_5p | 0 | 0.0 | 0.00 | 3 | 0.3 | 0.01 | 1 | 0.1 | | 0.00 | 0 | 0.0 | 0.00 |
| hsa_miR_4652_3p | 86 | 7.5 | 0.26 | 110 | 9.2 | 0.24 | 133 | 17.9 | | 0.75 | 200 | 26.3 | 0.49 |
| hsa_miR_4654 | 562 | 48.8 | 2.17 | 618 | 51.8 | 1.85 | 380 | 51.1 | | 1.99 | 369 | 48.6 | 1.46 |
| hsa_miR_4658 | 3 | 0.3 | 0.00 | 51 | 4.3 | 0.05 | 1 | 0.1 | | 0.00 | 8 | 1.1 | 0.02 |
| hsa_miR_466 | 182 | 15.8 | 0.73 | 294 | 24.7 | 0.57 | 215 | 28.9 | | 1.83 | 242 | 31.8 | 1.11 |
| hsa_miR_4676_5p | 463 | 40.2 | 1.52 | 588 | 49.3 | 1.51 | 340 | 45.8 | | 1.56 | 357 | 47.0 | 1.23 |
| hsa_miR_4681 | 47 | 4.1 | 0.07 | 55 | 4.6 | 0.04 | 32 | 4.3 | | 0.08 | 34 | 4.5 | 0.02 |
| hsa_miR_4682 | 429 | 37.3 | 1.19 | 521 | 43.7 | 1.04 | 261 | 35.1 | | 0.97 | 308 | 40.5 | 0.81 |
| hsa_miR_4684_3p | 573 | 49.8 | 1.98 | 712 | 59.7 | 2.26 | 308 | 41.5 | | 1.23 | 400 | 52.6 | 1.39 |
| hsa_miR_4685_3p | 7 | 0.6 | 0.02 | 35 | 2.9 | 0.02 | 3 | 0.4 | | 0.01 | 3 | 0.4 | 0.01 |
| hsa_miR_4687_5p | 2 | 0.2 | 0.01 | 2 | 0.2 | 0.01 | 1 | 0.1 | | 0.00 | 0 | 0.0 | 0.00 |
| hsa_miR_4692 | 4 | 0.3 | 0.01 | 5 | 0.4 | 0.02 | 3 | 0.4 | | 0.01 | 1 | 0.1 | 0.00 |
| hsa_miR_4694_3p | 198 | 17.2 | 0.66 | 368 | 30.9 | 0.97 | 110 | 14.8 | | 0.53 | 233 | 30.7 | 0.76 |
| hsa_miR_4695_3p | 44 | 3.8 | 0.23 | 74 | 6.2 | 0.17 | 32 | 4.3 | | 0.18 | 132 | 17.4 | 0.30 |
| hsa_miR_4697_3p | 11 | 1.0 | 0.11 | 10 | 0.8 | 0.07 | 4 | 0.5 | | 0.02 | 85 | 11.2 | 0.13 |
| hsa_miR_4700_3p | 133 | 11.6 | 0.47 | 262 | 22.0 | 0.56 | 57 | 7.7 | | 0.53 | 194 | 25.5 | 0.57 |
| hsa_miR_4700_5p | 269 | 23.4 | 0.52 | 291 | 24.4 | 0.36 | 183 | 24.6 | | 0.52 | 215 | 28.3 | 0.32 |
| hsa_miR_4701_5p | 348 | 30.2 | 2.68 | 529 | 44.4 | 2.74 | 291 | 39.2 | | 3.59 | 422 | 55.5 | 4.12 |
| hsa_miR_4709_3p | 448 | 38.9 | 1.01 | 551 | 46.2 | 0.90 | 251 | 33.8 | | 0.77 | 311 | 40.9 | 0.65 |
| hsa_miR_4711_5p | 44 | 3.8 | 0.09 | 96 | 8.1 | 0.13 | 33 | 4.4 | | 0.10 | 99 | 13.0 | 0.14 |
| hsa_miR_4713_5p | 15 | 1.3 | 0.16 | 117 | 9.8 | 0.25 | 7 | 0.9 | | 0.10 | 92 | 12.1 | 0.30 |
| hsa_miR_4714_3p | 2 | 0.2 | 0.00 | 3 | 0.3 | 0.00 | 0 | 0.0 | | 0.00 | 2 | 0.3 | 0.01 |
| hsa_miR_4716_5p | 41 | 3.6 | 0.22 | 121 | 10.2 | 0.18 | 49 | 6.6 | | 0.35 | 144 | 18.9 | 0.56 |
| hsa_miR_4719 | 17 | 1.5 | 0.03 | 94 | 7.9 | 0.06 | 4 | 0.5 | | 0.01 | 8 | 1.1 | 0.02 |
| hsa_miR_4722_5p | 566 | 49.2 | 2.85 | 742 | 62.2 | 3.50 | 340 | 45.8 | | 2.14 | 447 | 58.8 | 2.70 |
| hsa_miR_4723_3p | 0 | 0.0 | 0.00 | 2 | 0.2 | 0.01 | 1 | 0.1 | | 0.01 | 1 | 0.1 | 0.01 |
| hsa_miR_4726_3p | 11 | 1.0 | 0.07 | 10 | 0.8 | 0.03 | 1 | 0.1 | | 0.01 | 113 | 14.9 | 0.09 |
| hsa_miR_4727_3p | 198 | 17.2 | 0.34 | 251 | 21.1 | 0.31 | 125 | 16.8 | | 0.34 | 172 | 22.6 | 0.27 |
| hsa_miR_4729 | 1 | 0.1 | 0.00 | 18 | 1.5 | 0.06 | 0 | 0.0 | | 0.00 | 3 | 0.4 | 0.01 |
| hsa_miR_4730 | 488 | 42.4 | 4.88 | 582 | 48.8 | 4.31 | 402 | 54.1 | | 6.90 | 516 | 67.9 | 7.36 |
| hsa_miR_4731_3p | 370 | 32.1 | 1.06 | 456 | 38.3 | 1.03 | 243 | 32.7 | | 0.94 | 308 | 40.5 | 0.93 |
| hsa_miR_4732_3p | 3 | 0.3 | 0.04 | 1 | 0.1 | 0.01 | 0 | 0.0 | | 0.00 | 1 | 0.1 | 0.05 |
| hsa_miR_4737 | 250 | 21.7 | 0.45 | 320 | 26.8 | 0.37 | 183 | 24.6 | | 0.51 | 242 | 31.8 | 0.37 |
| hsa_miR_4740_3p | 61 | 5.3 | 0.21 | 165 | 13.8 | 0.21 | 14 | 1.9 | | 0.06 | 88 | 11.6 | 0.16 |
| hsa_miR_4754 | 15 | 1.3 | 0.03 | 64 | 5.4 | 0.09 | 5 | 0.7 | | 0.02 | 8 | 1.1 | 0.02 |
| hsa_miR_4760_3p | 1 | 0.1 | 0.01 | 0 | 0.0 | 0.00 | 0 | 0.0 | | 0.00 | 1 | 0.1 | 0.01 |
| hsa_miR_4763_5p | 176 | 15.3 | 0.81 | 350 | 29.4 | 0.77 | 160 | 21.5 | | 0.94 | 288 | 37.9 | 1.24 |
| hsa_miR_4764_5p | 1 | 0.1 | 0.00 | 40 | 3.4 | 0.01 | 1 | 0.1 | | 0.00 | 36 | 4.7 | 0.02 |
| hsa_miR_4779 | 0 | 0.0 | 0.00 | 2 | 0.2 | 0.00 | 1 | 0.1 | | 0.00 | 0 | 0.0 | 0.00 |
| hsa_miR_4780 | 8 | 0.7 | 0.08 | 5 | 0.4 | 0.04 | 1 | 0.1 | | 0.02 | 51 | 6.7 | 0.15 |
| hsa_miR_4783_5p | 7 | 0.6 | 0.01 | 46 | 3.9 | 0.03 | 5 | 0.7 | | 0.02 | 42 | 5.5 | 0.02 |
| hsa_miR_4786_3p | 7 | 0.6 | 0.01 | 4 | 0.3 | 0.00 | 1 | 0.1 | | 0.00 | 2 | 0.3 | 0.01 |
| hsa_miR_4796_3p | 6 | 0.5 | 0.01 | 50 | 4.2 | 0.05 | 9 | 1.2 | | 0.02 | 82 | 10.8 | 0.05 |
| hsa_miR_4801 | 1 | 0.1 | 0.00 | 6 | 0.5 | 0.01 | 3 | 0.4 | | 0.02 | 51 | 6.7 | 0.02 |
| hsa_miR_4804_3p | 2 | 0.2 | 0.01 | 1 | 0.1 | 0.01 | 0 | 0.0 | | 0.00 | 0 | 0.0 | 0.00 |
| hsa_miR_483_3p | 460 | 40.0 | 5.47 | 513 | 43.0 | 2.14 | 358 | 48.2 | | 6.49 | 436 | 57.4 | 3.81 |
| hsa_miR_484 | 216 | 18.8 | 0.63 | 332 | 27.9 | 0.64 | 239 | 32.2 | | 1.03 | 344 | 45.3 | 0.92 |
| hsa_miR_485_3p | 34 | 3.0 | 0.23 | 60 | 5.0 | 0.19 | 34 | 4.6 | | 0.30 | 84 | 11.1 | 0.46 |
| hsa_miR_487b | 275 | 23.9 | 0.83 | 398 | 33.4 | 0.91 | 263 | 35.4 | | 1.19 | 381 | 50.1 | 1.35 |
| hsa_miR_490_3p | 22 | 1.9 | 0.03 | 16 | 1.3 | 0.02 | 11 | 1.5 | | 0.02 | 44 | 5.8 | 0.02 |
| hsa_miR_491_3p | 7 | 0.6 | 0.02 | 14 | 1.2 | 0.01 | 2 | 0.3 | | 0.00 | 49 | 6.4 | 0.18 |
| hsa_miR_491_5p | 4 | 0.3 | 0.00 | 8 | 0.7 | 0.01 | 1 | 0.1 | | 0.00 | 49 | 6.4 | 0.03 |
| hsa_miR_497_5p | 239 | 20.8 | 0.99 | 820 | 68.8 | 4.37 | 187 | 25.2 | | 0.93 | 570 | 75.0 | 4.52 |
| hsa_miR_5006_3p | 1 | 0.1 | 0.00 | 14 | 1.2 | 0.01 | 1 | 0.1 | | 0.00 | 1 | 0.1 | 0.00 |
| hsa_miR_5008_3p | 261 | 22.7 | 0.82 | 247 | 20.7 | 0.30 | 157 | 21.1 | | 0.68 | 168 | 22.1 | 0.27 |
| hsa_miR_5010_3p | 35 | 3.0 | 0.26 | 25 | 2.1 | 0.19 | 33 | 4.4 | | 0.20 | 124 | 16.3 | 0.72 |
| hsa_miR_502_3p | 140 | 12.2 | 0.36 | 113 | 9.5 | 0.17 | 98 | 13.2 | | 0.37 | 120 | 15.8 | 0.20 |
| hsa_miR_503_5p | 20 | 1.7 | 0.05 | 3 | 0.3 | 0.01 | 16 | 2.2 | | 0.09 | 2 | 0.3 | 0.01 |
| hsa_miR_504 | 1 | 0.1 | 0.01 | 1 | 0.1 | 0.01 | 0 | 0.0 | | 0.00 | 3 | 0.4 | 0.00 |
| hsa_miR_5047 | 10 | 0.9 | 0.01 | 9 | 0.8 | 0.01 | 4 | 0.5 | | 0.01 | 30 | 3.9 | 0.01 |
| hsa_miR_505_3p | 57 | 5.0 | 0.15 | 40 | 3.4 | 0.03 | 42 | 5.7 | | 0.14 | 6 | 0.8 | 0.01 |
| hsa_miR_505_5p | 90 | 7.8 | 0.07 | 99 | 8.3 | 0.04 | 69 | 9.3 | | 0.08 | 106 | 13.9 | 0.05 |
| hsa_miR_506_3p | 1 | 0.1 | 0.01 | 3 | 0.3 | 0.00 | 0 | 0.0 | | 0.00 | 1 | 0.1 | 0.00 |
| hsa_miR_506_5p | 93 | 8.1 | 0.15 | 217 | 18.2 | 0.21 | 28 | 3.8 | | 0.06 | 103 | 13.6 | 0.09 |
| hsa_miR_508_3p | 3 | 0.3 | 0.00 | 3 | 0.3 | 0.00 | 0 | 0.0 | | 0.00 | 3 | 0.4 | 0.01 |
| hsa_miR_5095 | 438 | 38.1 | 1.00 | 560 | 47.0 | 0.98 | 305 | 41.0 | | 1.00 | 365 | 48.0 | 1.06 |
| hsa_miR_510 | 236 | 20.5 | 0.42 | 316 | 26.5 | 0.36 | 114 | 15.3 | | 0.29 | 194 | 25.5 | 0.23 |
| hsa_miR_513a_3p | 130 | 11.3 | 0.22 | 315 | 26.4 | 0.40 | 46 | 6.2 | | 0.10 | 153 | 20.1 | 0.22 |
| hsa_miR_513c_3p | 309 | 26.8 | 1.13 | 519 | 43.5 | 1.70 | 195 | 26.2 | | 1.11 | 345 | 45.4 | 1.67 |
| hsa_miR_516a_3p | 3 | 0.3 | 0.01 | 6 | 0.5 | 0.01 | 2 | 0.3 | | 0.01 | 2 | 0.3 | 0.01 |
| hsa_miR_5188 | 12 | 1.0 | 0.04 | 37 | 3.1 | 0.10 | 11 | 1.5 | | 0.09 | 22 | 2.9 | 0.05 |
| hsa_miR_518c_5p | 373 | 32.4 | 1.04 | 553 | 46.4 | 1.43 | 248 | 33.4 | | 0.90 | 298 | 39.2 | 1.02 |
| hsa_miR_518e_5p | 30 | 2.6 | 0.06 | 51 | 4.3 | 0.04 | 8 | 1.1 | | 0.02 | 2 | 0.3 | 0.00 |
| hsa_miR_5192 | 45 | 3.9 | 0.08 | 101 | 8.5 | 0.08 | 7 | 0.9 | | 0.01 | 31 | 4.1 | 0.02 |
| hsa_miR_5193 | 5 | 0.4 | 0.02 | 1 | 0.1 | 0.00 | 8 | 1.1 | | 0.06 | 34 | 4.5 | 0.03 |
| hsa_miR_5196_3p | 15 | 1.3 | 0.05 | 20 | 1.7 | 0.05 | 18 | 2.4 | | 0.10 | 64 | 8.4 | 0.15 |
| hsa_miR_5197_3p | 1 | 0.1 | 0.01 | 1 | 0.1 | 0.00 | 0 | 0.0 | | 0.00 | 0 | 0.0 | 0.00 |
| hsa_miR_519d | 4 | 0.3 | 0.06 | 1 | 0.1 | 0.01 | 1 | 0.1 | | 0.01 | 1 | 0.1 | 0.03 |
| hsa_miR_519e_3p | 3 | 0.3 | 0.02 | 1 | 0.1 | 0.01 | 0 | 0.0 | | 0.00 | 1 | 0.1 | 0.03 |
| hsa_miR_519e_5p | 493 | 42.8 | 1.71 | 648 | 54.4 | 1.97 | 342 | 46.0 | | 1.55 | 400 | 52.6 | 1.62 |
| hsa_miR_520c_3p | 2 | 0.2 | 0.02 | 1 | 0.1 | 0.00 | 2 | 0.3 | | 0.01 | 0 | 0.0 | 0.00 |
| hsa_miR_520d_3p | 160 | 13.9 | 0.62 | 331 | 27.8 | 0.69 | 45 | 6.1 | | 0.25 | 190 | 25.0 | 0.42 |
| hsa_miR_521 | 2 | 0.2 | 0.02 | 0 | 0.0 | 0.00 | 0 | 0.0 | | 0.00 | 1 | 0.1 | 0.03 |
| hsa_miR_525_5p | 505 | 43.9 | 1.25 | 629 | 52.8 | 1.58 | 346 | 46.6 | | 1.10 | 329 | 43.3 | 1.02 |
| hsa_miR_532_3p | 287 | 24.9 | 0.97 | 173 | 14.5 | 0.34 | 302 | 40.6 | | 1.53 | 324 | 42.6 | 0.81 |
| hsa_miR_532_5p | 417 | 36.2 | 2.34 | 184 | 15.4 | 0.43 | 341 | 45.9 | | 2.70 | 198 | 26.1 | 0.51 |
| hsa_miR_541_3p | 45 | 3.9 | 0.03 | 113 | 9.5 | 0.04 | 20 | 2.7 | | 0.02 | 92 | 12.1 | 0.03 |
| hsa_miR_542_3p | 254 | 22.1 | 1.06 | 391 | 32.8 | 1.31 | 134 | 18.0 | | 0.78 | 263 | 34.6 | 1.01 |
| hsa_miR_548a_5p | 29 | 2.5 | 0.06 | 169 | 14.2 | 0.08 | 1 | 0.1 | | 0.01 | 1 | 0.1 | 0.01 |
| hsa_miR_548aa | 198 | 17.2 | 0.71 | 376 | 31.5 | 0.85 | 218 | 29.3 | | 1.15 | 354 | 46.6 | 1.31 |
| hsa_miR_548ae | 184 | 16.0 | 0.70 | 322 | 27.0 | 0.68 | 11 | 1.5 | | 0.03 | 42 | 5.5 | 0.04 |
| hsa_miR_548aj_3p | 175 | 15.2 | 0.48 | 320 | 26.8 | 0.48 | 10 | 1.3 | | 0.02 | 40 | 5.3 | 0.02 |
| hsa_miR_548aj_5p | 30 | 2.6 | 0.12 | 167 | 14.0 | 0.16 | 0 | 0.0 | | 0.00 | 0 | 0.0 | 0.00 |
| hsa_miR_548am_5p | 167 | 14.5 | 0.36 | 331 | 27.8 | 0.44 | 100 | 13.5 | | 0.36 | 231 | 30.4 | 0.51 |
| hsa_miR_548ap_3p | 216 | 18.8 | 0.63 | 344 | 28.9 | 0.56 | 25 | 3.4 | | 0.04 | 43 | 5.7 | 0.04 |
| hsa_miR_548ap_5p | 25 | 2.2 | 0.12 | 156 | 13.1 | 0.13 | 0 | 0.0 | | 0.00 | 0 | 0.0 | 0.00 |
| hsa_miR_548av_5p | 10 | 0.9 | 0.04 | 84 | 7.0 | 0.04 | 0 | 0.0 | | 0.00 | 0 | 0.0 | 0.00 |
| hsa_miR_548aw | 43 | 3.7 | 0.11 | 171 | 14.3 | 0.21 | 16 | 2.2 | | 0.06 | 112 | 14.7 | 0.16 |
| hsa_miR_548c_3p | 203 | 17.6 | 0.54 | 346 | 29.0 | 0.55 | 20 | 2.7 | | 0.04 | 45 | 5.9 | 0.04 |
| hsa_miR_548d_5p | 105 | 9.1 | 0.20 | 262 | 22.0 | 0.23 | 38 | 5.1 | | 0.08 | 135 | 17.8 | 0.11 |
| hsa_miR_548f | 109 | 9.5 | 1.18 | 253 | 21.2 | 1.21 | 2 | 0.3 | | 0.01 | 1 | 0.1 | 0.01 |
| hsa_miR_548g_3p | 24 | 2.1 | 0.10 | 116 | 9.7 | 0.09 | 0 | 0.0 | | 0.00 | 0 | 0.0 | 0.00 |
| hsa_miR_548h_3p | 23 | 2.0 | 0.07 | 92 | 7.7 | 0.08 | 49 | 6.6 | | 0.20 | 63 | 8.3 | 0.09 |
| hsa_miR_548n | 35 | 3.0 | 0.15 | 160 | 13.4 | 0.16 | 0 | 0.0 | | 0.00 | 0 | 0.0 | 0.00 |
| hsa_miR_548x_3p | 181 | 15.7 | 0.67 | 325 | 27.3 | 0.69 | 14 | 1.9 | | 0.03 | 43 | 5.7 | 0.04 |
| hsa_miR_551a | 1 | 0.1 | 0.01 | 1 | 0.1 | 0.00 | 0 | 0.0 | | 0.00 | 0 | 0.0 | 0.00 |
| hsa_miR_551b_5p | 530 | 46.0 | 1.09 | 612 | 51.3 | 1.00 | 343 | 46.2 | | 0.91 | 344 | 45.3 | 0.62 |
| hsa_miR_552 | 98 | 8.5 | 0.37 | 4 | 0.3 | 0.01 | 86 | 11.6 | | 0.48 | 6 | 0.8 | 0.04 |
| hsa_miR_556_5p | 2 | 0.2 | 0.00 | 0 | 0.0 | 0.00 | 0 | 0.0 | | 0.00 | 1 | 0.1 | 0.01 |
| hsa_miR_5571_5p | 24 | 2.1 | 0.05 | 141 | 11.8 | 0.32 | 10 | 1.3 | | 0.03 | 131 | 17.2 | 0.21 |
| hsa_miR_5584_3p | 12 | 1.0 | 0.08 | 16 | 1.3 | 0.05 | 5 | 0.7 | | 0.04 | 6 | 0.8 | 0.06 |
| hsa_miR_5584_5p | 261 | 22.7 | 0.52 | 415 | 34.8 | 0.60 | 183 | 24.6 | | 0.57 | 277 | 36.4 | 0.58 |
| hsa_miR_5588_3p | 3 | 0.3 | 0.01 | 3 | 0.3 | 0.01 | 4 | 0.5 | | 0.03 | 2 | 0.3 | 0.01 |
| hsa_miR_5591_3p | 35 | 3.0 | 0.05 | 94 | 7.9 | 0.06 | 40 | 5.4 | | 0.11 | 108 | 14.2 | 0.09 |
| hsa_miR_5591_5p | 1 | 0.1 | 0.00 | 5 | 0.4 | 0.00 | 1 | 0.1 | | 0.00 | 30 | 3.9 | 0.01 |
| hsa_miR_563 | 1 | 0.1 | 0.00 | 1 | 0.1 | 0.00 | 0 | 0.0 | | 0.00 | 0 | 0.0 | 0.00 |
| hsa_miR_567 | 2 | 0.2 | 0.01 | 7 | 0.6 | 0.00 | 3 | 0.4 | | 0.00 | 2 | 0.3 | 0.00 |
| hsa_miR_5681b | 25 | 2.2 | 0.25 | 63 | 5.3 | 0.20 | 10 | 1.3 | | 0.12 | 111 | 14.6 | 0.26 |
| hsa_miR_5685 | 272 | 23.6 | 0.96 | 533 | 44.7 | 1.49 | 95 | 12.8 | | 0.41 | 294 | 38.7 | 0.94 |
| hsa_miR_5686 | 30 | 2.6 | 0.07 | 189 | 15.9 | 0.13 | 41 | 5.5 | | 0.12 | 112 | 14.7 | 0.21 |
| hsa_miR_5689 | 2 | 0.2 | 0.00 | 1 | 0.1 | 0.00 | 0 | 0.0 | | 0.00 | 0 | 0.0 | 0.00 |
| hsa_miR_569 | 0 | 0.0 | 0.00 | 0 | 0.0 | 0.00 | 1 | 0.1 | | 0.01 | 1 | 0.1 | 0.00 |
| hsa_miR_5692a | 43 | 3.7 | 0.07 | 73 | 6.1 | 0.07 | 11 | 1.5 | | 0.02 | 34 | 4.5 | 0.02 |
| hsa_miR_5692b | 1 | 0.1 | 0.00 | 0 | 0.0 | 0.00 | 0 | 0.0 | | 0.00 | 1 | 0.1 | 0.02 |
| hsa_miR_5700 | 341 | 29.6 | 1.15 | 477 | 40.0 | 1.23 | 167 | 22.5 | | 0.67 | 268 | 35.3 | 0.63 |
| hsa_miR_5701 | 10 | 0.9 | 0.02 | 2 | 0.2 | 0.00 | 4 | 0.5 | | 0.01 | 0 | 0.0 | 0.00 |
| hsa_miR_5704 | 3 | 0.3 | 0.02 | 1 | 0.1 | 0.01 | 1 | 0.1 | | 0.01 | 1 | 0.1 | 0.03 |
| hsa_miR_570_3p | 29 | 2.5 | 0.06 | 167 | 14.0 | 0.08 | 1 | 0.1 | | 0.00 | 0 | 0.0 | 0.00 |
| hsa_miR_571 | 17 | 1.5 | 0.06 | 1 | 0.1 | 0.00 | 14 | 1.9 | | 0.07 | 1 | 0.1 | 0.01 |
| hsa_miR_573 | 5 | 0.4 | 0.02 | 7 | 0.6 | 0.00 | 1 | 0.1 | | 0.01 | 0 | 0.0 | 0.00 |
| hsa_miR_584_3p | 1 | 0.1 | 0.00 | 2 | 0.2 | 0.00 | 0 | 0.0 | | 0.00 | 29 | 3.8 | 0.01 |
| hsa_miR_585 | 7 | 0.6 | 0.01 | 1 | 0.1 | 0.00 | 6 | 0.8 | | 0.01 | 1 | 0.1 | 0.00 |
| hsa_miR_589_3p | 5 | 0.4 | 0.00 | 1 | 0.1 | 0.00 | 0 | 0.0 | | 0.00 | 0 | 0.0 | 0.00 |
| hsa_miR_589_5p | 1 | 0.1 | 0.00 | 7 | 0.6 | 0.02 | 0 | 0.0 | | 0.00 | 0 | 0.0 | 0.00 |
| hsa_miR_590_5p | 237 | 20.6 | 0.88 | 420 | 35.2 | 1.11 | 119 | 16.0 | | 0.56 | 213 | 28.0 | 0.59 |
| hsa_miR_593_3p | 1 | 0.1 | 0.00 | 7 | 0.6 | 0.00 | 0 | 0.0 | | 0.00 | 0 | 0.0 | 0.00 |
| hsa_miR_604 | 13 | 1.1 | 0.02 | 62 | 5.2 | 0.02 | 11 | 1.5 | | 0.02 | 78 | 10.3 | 0.02 |
| hsa_miR_605 | 239 | 20.8 | 0.75 | 485 | 40.7 | 0.98 | 121 | 16.3 | | 0.46 | 270 | 35.5 | 0.64 |
| hsa_miR_6071 | 189 | 16.4 | 0.48 | 442 | 37.1 | 0.74 | 95 | 12.8 | | 0.26 | 274 | 36.1 | 0.70 |
| hsa_miR_6081 | 302 | 26.2 | 0.78 | 485 | 40.7 | 0.83 | 243 | 32.7 | | 0.83 | 338 | 44.5 | 0.90 |
| hsa_miR_609 | 4 | 0.3 | 0.01 | 5 | 0.4 | 0.02 | 2 | 0.3 | | 0.01 | 51 | 6.7 | 0.03 |
| hsa_miR_613 | 4 | 0.3 | 0.02 | 1 | 0.1 | 0.01 | 0 | 0.0 | | 0.00 | 0 | 0.0 | 0.00 |
| hsa_miR_6130 | 16 | 1.4 | 0.03 | 39 | 3.3 | 0.02 | 3 | 0.4 | | 0.00 | 32 | 4.2 | 0.02 |
| hsa_miR_616_5p | 2 | 0.2 | 0.00 | 2 | 0.2 | 0.00 | 0 | 0.0 | | 0.00 | 0 | 0.0 | 0.00 |
| hsa_miR_625_3p | 2 | 0.2 | 0.01 | 2 | 0.2 | 0.01 | 0 | 0.0 | | 0.00 | 1 | 0.1 | 0.00 |
| hsa_miR_625_5p | 99 | 8.6 | 0.26 | 127 | 10.7 | 0.18 | 39 | 5.2 | | 0.12 | 99 | 13.0 | 0.09 |
| hsa_miR_626 | 2 | 0.2 | 0.01 | 3 | 0.3 | 0.01 | 0 | 0.0 | | 0.00 | 0 | 0.0 | 0.00 |
| hsa_miR_627 | 3 | 0.3 | 0.01 | 5 | 0.4 | 0.00 | 3 | 0.4 | | 0.00 | 0 | 0.0 | 0.00 |
| hsa_miR_629_5p | 3 | 0.3 | 0.00 | 6 | 0.5 | 0.02 | 3 | 0.4 | | 0.00 | 1 | 0.1 | 0.00 |
| hsa_miR_632 | 403 | 35.0 | 1.45 | 522 | 43.8 | 1.51 | 232 | 31.2 | | 1.15 | 295 | 38.8 | 1.24 |
| hsa_miR_634 | 27 | 2.3 | 0.09 | 52 | 4.4 | 0.11 | 20 | 2.7 | | 0.07 | 127 | 16.7 | 0.22 |
| hsa_miR_637 | 6 | 0.5 | 0.01 | 2 | 0.2 | 0.01 | 0 | 0.0 | | 0.00 | 0 | 0.0 | 0.00 |
| hsa_miR_640 | 111 | 9.6 | 0.18 | 173 | 14.5 | 0.18 | 73 | 9.8 | | 0.15 | 176 | 23.2 | 0.24 |
| hsa_miR_645 | 509 | 44.2 | 1.82 | 137 | 11.5 | 0.12 | 354 | 47.6 | | 2.20 | 151 | 19.9 | 0.11 |
| hsa_miR_647 | 2 | 0.2 | 0.01 | 2 | 0.2 | 0.01 | 0 | 0.0 | | 0.00 | 0 | 0.0 | 0.00 |
| hsa_miR_6503_3p | 4 | 0.3 | 0.01 | 9 | 0.8 | 0.01 | 4 | 0.5 | | 0.01 | 33 | 4.3 | 0.02 |
| hsa_miR_6507_3p | 3 | 0.3 | 0.02 | 3 | 0.3 | 0.01 | 0 | 0.0 | | 0.00 | 1 | 0.1 | 0.03 |
| hsa_miR_6509_3p | 12 | 1.0 | 0.09 | 18 | 1.5 | 0.10 | 5 | 0.7 | | 0.08 | 26 | 3.4 | 0.08 |
| hsa_miR_6511a_3p | 60 | 5.2 | 0.50 | 116 | 9.7 | 0.35 | 40 | 5.4 | | 0.39 | 130 | 17.1 | 0.57 |
| hsa_miR_6511b_3p | 8 | 0.7 | 0.08 | 4 | 0.3 | 0.03 | 2 | 0.3 | | 0.02 | 2 | 0.3 | 0.06 |
| hsa_miR_6514_3p | 21 | 1.8 | 0.09 | 21 | 1.8 | 0.10 | 31 | 4.2 | | 0.21 | 62 | 8.2 | 0.40 |
| hsa_miR_6515_5p | 333 | 28.9 | 1.01 | 919 | 77.1 | 3.99 | 188 | 25.3 | | 0.91 | 624 | 82.1 | 4.51 |
| hsa_miR_652_3p | 201 | 17.5 | 0.63 | 155 | 13.0 | 0.24 | 170 | 22.9 | | 0.70 | 192 | 25.3 | 0.29 |
| hsa_miR_654_3p | 1 | 0.1 | 0.00 | 3 | 0.3 | 0.01 | 5 | 0.7 | | 0.02 | 4 | 0.5 | 0.04 |
| hsa_miR_658 | 480 | 41.7 | 0.55 | 699 | 58.6 | 0.76 | 265 | 35.7 | | 0.41 | 393 | 51.7 | 0.54 |
| hsa_miR_659_5p | 303 | 26.3 | 1.18 | 515 | 43.2 | 1.67 | 146 | 19.7 | | 0.77 | 296 | 38.9 | 1.20 |
| hsa_miR_660_5p | 119 | 10.3 | 0.40 | 26 | 2.2 | 0.06 | 93 | 12.5 | | 0.39 | 13 | 1.7 | 0.05 |
| hsa_miR_661 | 7 | 0.6 | 0.01 | 5 | 0.4 | 0.01 | 4 | 0.5 | | 0.01 | 0 | 0.0 | 0.00 |
| hsa_miR_664a_3p | 419 | 36.4 | 2.36 | 518 | 43.5 | 1.85 | 378 | 50.9 | | 2.85 | 425 | 55.9 | 2.86 |
| hsa_miR_668 | 566 | 49.2 | 1.66 | 713 | 59.8 | 1.85 | 329 | 44.3 | | 1.22 | 419 | 55.1 | 1.58 |
| hsa_miR_670 | 158 | 13.7 | 0.49 | 296 | 24.8 | 0.55 | 82 | 11.0 | | 0.41 | 159 | 20.9 | 0.32 |
| hsa_miR_6715b_3p | 95 | 8.3 | 0.25 | 42 | 3.5 | 0.04 | 66 | 8.9 | | 0.26 | 34 | 4.5 | 0.02 |
| hsa_miR_6716_3p | 221 | 19.2 | 1.50 | 288 | 24.2 | 1.09 | 236 | 31.8 | | 2.53 | 296 | 38.9 | 2.41 |
| hsa_miR_6719_3p | 19 | 1.7 | 0.08 | 90 | 7.6 | 0.11 | 6 | 0.8 | | 0.03 | 111 | 14.6 | 0.26 |
| hsa_miR_671_3p | 397 | 34.5 | 0.57 | 573 | 48.1 | 0.74 | 225 | 30.3 | | 0.43 | 340 | 44.7 | 0.56 |
| hsa_miR_6721_5p | 7 | 0.6 | 0.02 | 9 | 0.8 | 0.03 | 1 | 0.1 | | 0.00 | 1 | 0.1 | 0.00 |
| hsa_miR_6722_5p | 46 | 4.0 | 0.25 | 31 | 2.6 | 0.21 | 25 | 3.4 | | 0.21 | 97 | 12.8 | 0.27 |
| hsa_miR_675_3p | 70 | 6.1 | 0.28 | 3 | 0.3 | 0.01 | 57 | 7.7 | | 0.27 | 2 | 0.3 | 0.01 |
| hsa_miR_675_5p | 101 | 8.8 | 0.14 | 171 | 14.3 | 0.07 | 58 | 7.8 | | 0.13 | 76 | 10.0 | 0.03 |
| hsa_miR_744_3p | 7 | 0.6 | 0.02 | 1 | 0.1 | 0.01 | 3 | 0.4 | | 0.02 | 2 | 0.3 | 0.02 |
| hsa_miR_744_5p | 7 | 0.6 | 0.02 | 3 | 0.3 | 0.01 | 6 | 0.8 | | 0.03 | 30 | 3.9 | 0.01 |
| hsa_miR_764 | 2 | 0.2 | 0.01 | 2 | 0.2 | 0.01 | 0 | 0.0 | | 0.00 | 1 | 0.1 | 0.00 |
| hsa_miR_767_5p | 1 | 0.1 | 0.01 | 1 | 0.1 | 0.00 | 0 | 0.0 | | 0.00 | 0 | 0.0 | 0.00 |
| hsa_miR_7_2_3p | 2 | 0.2 | 0.01 | 2 | 0.2 | 0.00 | 0 | 0.0 | | 0.00 | 0 | 0.0 | 0.00 |
| hsa_miR_7_5p | 510 | 44.3 | 3.45 | 99 | 8.3 | 0.19 | 383 | 51.5 | | 3.61 | 110 | 14.5 | 0.19 |
| hsa_miR_873_3p | 82 | 7.1 | 0.26 | 120 | 10.1 | 0.25 | 36 | 4.8 | | 0.18 | 27 | 3.6 | 0.10 |
| hsa_miR_873_5p | 1 | 0.1 | 0.01 | 0 | 0.0 | 0.00 | 0 | 0.0 | | 0.00 | 4 | 0.5 | 0.02 |
| hsa_miR_885_3p | 68 | 5.9 | 0.06 | 70 | 5.9 | 0.04 | 17 | 2.3 | | 0.02 | 33 | 4.3 | 0.01 |
| hsa_miR_885_5p | 110 | 9.6 | 1.42 | 223 | 18.7 | 1.30 | 77 | 10.4 | | 1.23 | 219 | 28.8 | 1.63 |
| hsa_miR_890 | 137 | 11.9 | 0.11 | 259 | 21.7 | 0.14 | 81 | 10.9 | | 0.10 | 216 | 28.4 | 0.13 |
| hsa_miR_892c_5p | 1 | 0.1 | 0.00 | 1 | 0.1 | 0.00 | 1 | 0.1 | | 0.00 | 0 | 0.0 | 0.00 |
| hsa_miR_920 | 8 | 0.7 | 0.01 | 20 | 1.7 | 0.03 | 7 | 0.9 | | 0.05 | 35 | 4.6 | 0.05 |
| hsa_miR_92a_1_5p | 332 | 28.8 | 0.33 | 478 | 40.1 | 0.37 | 224 | 30.1 | | 0.33 | 319 | 42.0 | 0.39 |
| hsa_miR_92a_2_5p | 41 | 3.6 | 0.05 | 195 | 16.4 | 0.09 | 2 | 0.3 | | 0.00 | 36 | 4.7 | 0.01 |
| hsa_miR_92b_3p | 125 | 10.9 | 0.19 | 129 | 10.8 | 0.11 | 67 | 9.0 | | 0.11 | 132 | 17.4 | 0.12 |
| hsa_miR_92b_5p | 16 | 1.4 | 0.01 | 4 | 0.3 | 0.00 | 4 | 0.5 | | 0.00 | 2 | 0.3 | 0.00 |
| hsa_miR_934 | 930 | 80.8 | 3.91 | 555 | 46.6 | 0.71 | 641 | 86.3 | | 4.49 | 327 | 43.0 | 0.63 |
| hsa_miR_937_3p | 9 | 0.8 | 0.04 | 17 | 1.4 | 0.03 | 6 | 0.8 | | 0.01 | 54 | 7.1 | 0.09 |
| hsa_miR_939_3p | 48 | 4.2 | 0.15 | 78 | 6.5 | 0.09 | 40 | 5.4 | | 0.13 | 155 | 20.4 | 0.23 |
| hsa_miR_93_3p | 2 | 0.2 | 0.00 | 1 | 0.1 | 0.00 | 0 | 0.0 | | 0.00 | 2 | 0.3 | 0.00 |
| hsa_miR_942 | 2 | 0.2 | 0.02 | 7 | 0.6 | 0.00 | 0 | 0.0 | | 0.00 | 0 | 0.0 | 0.00 |
| hsa_miR_944 | 3 | 0.3 | 0.00 | 1 | 0.1 | 0.00 | 1 | 0.1 | | 0.00 | 0 | 0.0 | 0.00 |
| hsa_miR_95 | 115 | 10.0 | 0.48 | 4 | 0.3 | 0.02 | 114 | 15.3 | | 0.78 | 2 | 0.3 | 0.02 |
| hsa_miR_96_5p | 64 | 5.6 | 0.31 | 3 | 0.3 | 0.01 | 31 | 4.2 | | 0.15 | 1 | 0.1 | 0.00 |
| hsa_miR_98_5p | 354 | 30.8 | 1.40 | 200 | 16.8 | 0.31 | 274 | 36.9 | | 1.30 | 132 | 17.4 | 0.24 |
| hsa_miR_99b_5p | 482 | 41.9 | 3.08 | 528 | 44.3 | 1.90 | 413 | 55.6 | | 3.90 | 500 | 65.8 | 3.14 |

| Supplemental Table 3. Associations between infrequently expressed miRNAs and survival in rectal cancer cases | | | | | | | | | | |
| --- | --- | --- | --- | --- | --- | --- | --- | --- | --- | --- |
|  |  | Censored | | Died of CRC | |  |  |  | P-value | Q-value |
| miRNA | Expression Level | N | % | N | % | HR | (95% CI) | | Raw | Adjusted |
| hsa-miR-124-3p | <-1.77 | 15 | 3 | 15 | 6.7 | 1.75 | (1.03, | 2.96) | 0.038 | 0.42 |
|  | [-1.77, 2.08] | 471 | 94.8 | 201 | 89.7 | 1 |  |  |  |  |
|  | >2.08 | 11 | 2.2 | 8 | 3.6 | 1.72 | (0.84, | 3.51) | 0.137 | 0.45 |
| hsa-miR-151a-3p | <-1.77 | 13 | 2.6 | 8 | 3.6 | 1.28 | (0.63, | 2.63) | 0.494 | 0.75 |
|  | [-1.77, 2.08] | 244 | 49.1 | 127 | 56.7 | 1 |  |  |  |  |
|  | >2.08 | 240 | 48.3 | 89 | 39.7 | 0.76 | (0.58, | 1.00) | 0.046 | 0.37 |
| hsa-miR-1915-5p | <-1.77 | 29 | 5.8 | 7 | 3.1 | 0.41 | (0.19, | 0.87) | 0.021 | 0.38 |
|  | [-1.77, 2.08] | 452 | 90.9 | 208 | 92.9 | 1 |  |  |  |  |
|  | >2.08 | 16 | 3.2 | 9 | 4 | 1.48 | (0.75, | 2.94) | 0.263 | 0.49 |
| hsa-miR-199b-5p | <-1.77 | 28 | 5.6 | 14 | 6.3 | 0.92 | (0.52, | 1.60) | 0.756 | 0.81 |
|  | [-1.77, 2.08] | 276 | 55.5 | 140 | 62.5 | 1 |  |  |  |  |
|  | >2.08 | 193 | 38.8 | 70 | 31.3 | 0.7 | (0.52, | 0.93) | 0.016 | 0.25 |
| hsa-miR-203a | <-1.77 | 53 | 10.7 | 27 | 12.1 | 0.92 | (0.58, | 1.44) | 0.709 | 0.8 |
|  | [-1.77, 2.08] | 110 | 22.1 | 65 | 29 | 1 |  |  |  |  |
|  | >2.08 | 334 | 67.2 | 132 | 58.9 | 0.7 | (0.52, | 0.95) | 0.023 | 0.25 |
| hsa-miR-2278 | <-1.77 | 83 | 16.7 | 48 | 21.4 | 1.08 | (0.77, | 1.51) | 0.649 | 0.8 |
|  | [-1.77, 2.08] | 340 | 68.4 | 156 | 69.6 | 1 |  |  |  |  |
|  | >2.08 | 74 | 14.9 | 20 | 8.9 | 0.58 | (0.36, | 0.93) | 0.024 | 0.25 |
| hsa-miR-299-3p | <-1.77 | 82 | 16.5 | 44 | 19.6 | 1.44 | (1.03, | 2.02) | 0.034 | 0.42 |
|  | [-1.77, 2.08] | 382 | 76.9 | 169 | 75.4 | 1 |  |  |  |  |
|  | >2.08 | 33 | 6.6 | 11 | 4.9 | 1.01 | (0.54, | 1.87) | 0.982 | 0.98 |
| hsa-miR-3149 | <-1.77 | 152 | 30.6 | 84 | 37.5 | 1.37 | (1.01, | 1.84) | 0.04 | 0.42 |
|  | [-1.77, 2.08] | 207 | 41.6 | 92 | 41.1 | 1 |  |  |  |  |
|  | >2.08 | 138 | 27.8 | 48 | 21.4 | 0.87 | (0.61, | 1.25) | 0.453 | 0.57 |
| hsa-miR-3609 | <-1.77 | 81 | 16.3 | 50 | 22.3 | 1.5 | (1.07, | 2.09) | 0.017 | 0.38 |
|  | [-1.77, 2.08] | 296 | 59.6 | 123 | 54.9 | 1 |  |  |  |  |
|  | >2.08 | 120 | 24.1 | 51 | 22.8 | 0.82 | (0.59, | 1.15) | 0.25 | 0.48 |
| hsa-miR-3615 | <-1.77 | 68 | 13.7 | 41 | 18.3 | 1.07 | (0.76, | 1.53) | 0.69 | 0.8 |
|  | [-1.77, 2.08] | 415 | 83.5 | 169 | 75.4 | 1 |  |  |  |  |
|  | >2.08 | 14 | 2.8 | 14 | 6.3 | 2.48 | (1.41, | 4.34) | 0.002 | 0.23 |
| hsa-miR-374b-5p | <-1.77 | 12 | 2.4 | 6 | 2.7 | 1.27 | (0.56, | 2.89) | 0.57 | 0.77 |
|  | [-1.77, 2.08] | 309 | 62.2 | 156 | 69.6 | 1 |  |  |  |  |
|  | >2.08 | 176 | 35.4 | 62 | 27.7 | 0.67 | (0.50, | 0.90) | 0.008 | 0.23 |
| hsa-miR-378e | <-1.77 | 65 | 13.1 | 31 | 13.8 | 0.85 | (0.57, | 1.25) | 0.398 | 0.67 |
|  | [-1.77, 2.08] | 329 | 66.2 | 163 | 72.8 | 1 |  |  |  |  |
|  | >2.08 | 103 | 20.7 | 30 | 13.4 | 0.56 | (0.38, | 0.83) | 0.004 | 0.23 |
| hsa-miR-3922-5p | <-1.77 | 60 | 12.1 | 38 | 17 | 1.53 | (1.07, | 2.18) | 0.02 | 0.38 |
|  | [-1.77, 2.08] | 371 | 74.6 | 170 | 75.9 | 1 |  |  |  |  |
|  | >2.08 | 66 | 13.3 | 16 | 7.1 | 0.86 | (0.51, | 1.44) | 0.564 | 0.59 |
| hsa-miR-3944-5p | <-1.77 | 113 | 22.7 | 37 | 16.5 | 0.69 | (0.48, | 0.99) | 0.044 | 0.42 |
|  | [-1.77, 2.08] | 289 | 58.1 | 147 | 65.6 | 1 |  |  |  |  |
|  | >2.08 | 95 | 19.1 | 40 | 17.9 | 0.95 | (0.67, | 1.35) | 0.76 | 0.76 |
| hsa-miR-4282 | <-1.77 | 144 | 29 | 49 | 21.9 | 0.68 | (0.49, | 0.94) | 0.018 | 0.38 |
|  | [-1.77, 2.08] | 277 | 55.7 | 149 | 66.5 | 1 |  |  |  |  |
|  | >2.08 | 76 | 15.3 | 26 | 11.6 | 0.66 | (0.44, | 1.01) | 0.058 | 0.37 |
| hsa-miR-4300 | <-1.77 | 43 | 8.7 | 33 | 14.7 | 1.52 | (1.04, | 2.22) | 0.03 | 0.42 |
|  | [-1.77, 2.08] | 375 | 75.5 | 162 | 72.3 | 1 |  |  |  |  |
|  | >2.08 | 79 | 15.9 | 29 | 12.9 | 0.8 | (0.54, | 1.19) | 0.279 | 0.5 |
| hsa-miR-4324 | <-1.77 | 99 | 19.9 | 53 | 23.7 | 1.54 | (1.11, | 2.13) | 0.009 | 0.38 |
|  | [-1.77, 2.08] | 332 | 66.8 | 129 | 57.6 | 1 |  |  |  |  |
|  | >2.08 | 66 | 13.3 | 42 | 18.8 | 1.3 | (0.91, | 1.86) | 0.143 | 0.45 |
| hsa-miR-451b | <-1.77 | 35 | 7 | 25 | 11.2 | 1.63 | (1.07, | 2.49) | 0.024 | 0.38 |
|  | [-1.77, 2.08] | 441 | 88.7 | 191 | 85.3 | 1 |  |  |  |  |
|  | >2.08 | 21 | 4.2 | 8 | 3.6 | 0.85 | (0.41, | 1.75) | 0.661 | 0.66 |
| hsa-miR-4654 | <-1.77 | 93 | 18.7 | 40 | 17.9 | 0.94 | (0.66, | 1.34) | 0.733 | 0.8 |
|  | [-1.77, 2.08] | 254 | 51.1 | 134 | 59.8 | 1 |  |  |  |  |
|  | >2.08 | 150 | 30.2 | 50 | 22.3 | 0.69 | (0.50, | 0.96) | 0.026 | 0.25 |
| hsa-miR-4682 | <-1.77 | 62 | 12.5 | 46 | 20.5 | 1.65 | (1.17, | 2.32) | 0.004 | 0.38 |
|  | [-1.77, 2.08] | 373 | 75.1 | 146 | 65.2 | 1 |  |  |  |  |
|  | >2.08 | 62 | 12.5 | 32 | 14.3 | 1.47 | (0.99, | 2.17) | 0.056 | 0.37 |
| hsa-miR-518c-5p | <-1.77 | 75 | 15.1 | 45 | 20.1 | 1.53 | (1.10, | 2.14) | 0.012 | 0.38 |
|  | [-1.77, 2.08] | 360 | 72.4 | 163 | 72.8 | 1 |  |  |  |  |
|  | >2.08 | 62 | 12.5 | 16 | 7.1 | 0.74 | (0.44, | 1.25) | 0.265 | 0.49 |
| hsa-miR-532-5p | <-1.77 | 16 | 3.2 | 12 | 5.4 | 1.09 | (0.61, | 1.98) | 0.765 | 0.82 |
|  | [-1.77, 2.08] | 274 | 55.1 | 142 | 63.4 | 1 |  |  |  |  |
|  | >2.08 | 207 | 41.6 | 70 | 31.3 | 0.71 | (0.53, | 0.95) | 0.023 | 0.25 |
| hsa-miR-548aa | <-1.77 | 52 | 10.5 | 29 | 12.9 | 0.9 | (0.61, | 1.35) | 0.621 | 0.79 |
|  | [-1.77, 2.08] | 378 | 76.1 | 178 | 79.5 | 1 |  |  |  |  |
|  | >2.08 | 67 | 13.5 | 17 | 7.6 | 0.51 | (0.31, | 0.85) | 0.01 | 0.23 |
| hsa-miR-590-5p | <-1.77 | 46 | 9.3 | 19 | 8.5 | 0.78 | (0.49, | 1.26) | 0.317 | 0.65 |
|  | [-1.77, 2.08] | 397 | 79.9 | 194 | 86.6 | 1 |  |  |  |  |
|  | >2.08 | 54 | 10.9 | 11 | 4.9 | 0.41 | (0.22, | 0.75) | 0.004 | 0.23 |
| hsa-miR-6081 | <-1.77 | 66 | 13.3 | 32 | 14.3 | 1.01 | (0.69, | 1.47) | 0.974 | 0.97 |
|  | [-1.77, 2.08] | 381 | 76.7 | 180 | 80.4 | 1 |  |  |  |  |
|  | >2.08 | 50 | 10.1 | 12 | 5.4 | 0.52 | (0.29, | 0.95) | 0.032 | 0.28 |
| hsa-miR-675-5p | <-1.77 | 0 | 0 | 0 | 0 |  |  |  |  |  |
|  | [-1.77, 2.08] | 492 | 99 | 216 | 96.4 | 1 |  |  |  |  |
|  | >2.08 | 5 | 1 | 8 | 3.6 | 2.94 | (1.43, | 6.07) | 0.004 | 0.23 |

| Supplemental Table 4. Infrequently expressed miRNAs and associations with survival adjusted for MSI along with age, center, AJCC stage, and sex | | | | | | | | | | | |
| --- | --- | --- | --- | --- | --- | --- | --- | --- | --- | --- | --- |
|  |  |  | Censored | | Died of CRC | |  |  |  | P-values | |
| Study | miRNA |  | N | % | N | % | HR | (95% CI) | | Raw | Q |
| Overall | hsa-miR-124-3p | <-1.77 | 70 | 5.4 | 54 | 9.5 | 1.23 | (0.93, | 1.64) | 0.152 | 0.81 |
|  |  | [-1.77, 2.08] | 1184 | 92.0 | 488 | 85.9 | 1.00 |  |  |  |  |
|  |  | >2.08 | 33 | 2.6 | 26 | 4.6 | 1.94 | (1.30, | 2.91) | 0.001 | 0.16 |
|  | hsa-miR-132-3p | <-1.77 | 61 | 4.7 | 28 | 4.9 | 0.87 | (0.59, | 1.29) | 0.493 | 0.81 |
|  |  | [-1.77, 2.08] | 1170 | 90.9 | 503 | 88.6 | 1.00 |  |  |  |  |
|  |  | >2.08 | 56 | 4.4 | 37 | 6.5 | 1.43 | (1.01, | 2.03) | 0.045 | 0.42 |
|  | hsa-miR-143-5p | <-1.77 | 73 | 5.7 | 31 | 5.5 | 0.77 | (0.53, | 1.11) | 0.161 | 0.81 |
|  |  | [-1.77, 2.08] | 1177 | 91.5 | 489 | 86.1 | 1.00 |  |  |  |  |
|  |  | >2.08 | 37 | 2.9 | 48 | 8.5 | 1.59 | (1.17, | 2.15) | 0.003 | 0.16 |
|  | hsa-miR-145-3p | <-1.77 | 29 | 2.3 | 18 | 3.2 | 1.23 | (0.76, | 1.98) | 0.404 | 0.81 |
|  |  | [-1.77, 2.08] | 1252 | 97.3 | 535 | 94.2 | 1.00 |  |  |  |  |
|  |  | >2.08 | 6 | 0.5 | 15 | 2.6 | 3.32 | (1.97, | 5.59) | <.0001 | 0.16 |
|  | hsa-miR-152 | <-1.77 | 7 | 0.5 | 5 | 0.9 | 1.58 | (0.65, | 3.83) | 0.309 | 0.81 |
|  |  | [-1.77, 2.08] | 1263 | 98.1 | 548 | 96.5 | 1.00 |  |  |  |  |
|  |  | >2.08 | 17 | 1.3 | 15 | 2.6 | 1.80 | (1.07, | 3.03) | 0.026 | 0.32 |
|  | hsa-miR-192-3p | <-1.77 | 265 | 20.6 | 149 | 26.2 | 1.12 | (0.92, | 1.37) | 0.262 | 0.81 |
|  |  | [-1.77, 2.08] | 864 | 67.1 | 373 | 65.7 | 1.00 |  |  |  |  |
|  |  | >2.08 | 158 | 12.3 | 46 | 8.1 | 0.72 | (0.53, | 0.99) | 0.042 | 0.42 |
|  | hsa-miR-19a-3p | <-1.77 | 14 | 1.1 | 5 | 0.9 | 0.89 | (0.37, | 2.14) | 0.789 | 0.86 |
|  |  | [-1.77, 2.08] | 921 | 71.6 | 443 | 78.0 | 1.00 |  |  |  |  |
|  |  | >2.08 | 352 | 27.4 | 120 | 21.1 | 0.76 | (0.62, | 0.93) | 0.009 | 0.18 |
|  | hsa-miR-2110 | <-1.77 | 70 | 5.4 | 23 | 4.0 | 0.63 | (0.41, | 0.96) | 0.031 | 0.69 |
|  |  | [-1.77, 2.08] | 1173 | 91.1 | 528 | 93.0 | 1.00 |  |  |  |  |
|  |  | >2.08 | 44 | 3.4 | 17 | 3.0 | 1.20 | (0.73, | 1.99) | 0.469 | 0.87 |
|  | hsa-miR-30e-5p | <-1.77 | 437 | 34.0 | 208 | 36.6 | 0.87 | (0.72, | 1.04) | 0.128 | 0.81 |
|  |  | [-1.77, 2.08] | 638 | 49.6 | 287 | 50.5 | 1.00 |  |  |  |  |
|  |  | >2.08 | 212 | 16.5 | 73 | 12.9 | 0.69 | (0.53, | 0.89) | 0.005 | 0.16 |
|  | hsa-miR-3148 | <-1.77 | 13 | 1.0 | 12 | 2.1 | 2.09 | (1.14, | 3.81) | 0.017 | 0.56 |
|  |  | [-1.77, 2.08] | 1233 | 95.8 | 541 | 95.2 | 1.00 |  |  |  |  |
|  |  | >2.08 | 41 | 3.2 | 15 | 2.6 | 1.10 | (0.66, | 1.84) | 0.718 | 0.90 |
|  | hsa-miR-31-5p | <-1.77 | 19 | 1.5 | 7 | 1.2 | 0.65 | (0.31, | 1.39) | 0.268 | 0.81 |
|  |  | [-1.77, 2.08] | 1114 | 86.6 | 455 | 80.1 | 1.00 |  |  |  |  |
|  |  | >2.08 | 154 | 12.0 | 106 | 18.7 | 1.50 | (1.21, | 1.86) | 0.000 | 0.16 |
|  | hsa-miR-338-3p | <-1.77 | 34 | 2.6 | 27 | 4.8 | 1.75 | (1.18, | 2.58) | 0.005 | 0.56 |
|  |  | [-1.77, 2.08] | 1233 | 95.8 | 527 | 92.8 | 1.00 |  |  |  |  |
|  |  | >2.08 | 20 | 1.6 | 14 | 2.5 | 1.65 | (0.97, | 2.81) | 0.066 | 0.54 |
|  | hsa-miR-362-5p | <-1.77 | 27 | 2.1 | 11 | 1.9 | 0.65 | (0.35, | 1.18) | 0.157 | 0.81 |
|  |  | [-1.77, 2.08] | 912 | 70.9 | 439 | 77.3 | 1.00 |  |  |  |  |
|  |  | >2.08 | 348 | 27.0 | 118 | 20.8 | 0.68 | (0.55, | 0.84) | 0.000 | 0.16 |
|  | hsa-miR-374a-5p | <-1.77 | 16 | 1.2 | 7 | 1.2 | 0.68 | (0.32, | 1.45) | 0.322 | 0.81 |
|  |  | [-1.77, 2.08] | 996 | 77.4 | 478 | 84.2 | 1.00 |  |  |  |  |
|  |  | >2.08 | 275 | 21.4 | 83 | 14.6 | 0.63 | (0.50, | 0.80) | 0.000 | 0.16 |
|  | hsa-miR-374b-5p | <-1.77 | 28 | 2.2 | 21 | 3.7 | 1.24 | (0.79, | 1.93) | 0.349 | 0.81 |
|  |  | [-1.77, 2.08] | 891 | 69.2 | 408 | 71.8 | 1.00 |  |  |  |  |
|  |  | >2.08 | 368 | 28.6 | 139 | 24.5 | 0.80 | (0.66, | 0.97) | 0.024 | 0.32 |
|  | hsa-miR-4274 | <-1.77 | 68 | 5.3 | 37 | 6.5 | 1.09 | (0.78, | 1.53) | 0.610 | 0.82 |
|  |  | [-1.77, 2.08] | 1137 | 88.3 | 514 | 90.5 | 1.00 |  |  |  |  |
|  |  | >2.08 | 82 | 6.4 | 17 | 3.0 | 0.56 | (0.33, | 0.94) | 0.028 | 0.32 |
|  | hsa-miR-4324 | <-1.77 | 184 | 14.3 | 81 | 14.3 | 1.31 | (1.02, | 1.67) | 0.034 | 0.69 |
|  |  | [-1.77, 2.08] | 948 | 73.7 | 374 | 65.8 | 1.00 |  |  |  |  |
|  |  | >2.08 | 155 | 12.0 | 113 | 19.9 | 1.25 | (1.00, | 1.55) | 0.047 | 0.42 |
|  | hsa-miR-4642 | <-1.77 | 22 | 1.7 | 16 | 2.8 | 1.81 | (1.10, | 2.98) | 0.020 | 0.57 |
|  |  | [-1.77, 2.08] | 1209 | 93.9 | 527 | 92.8 | 1.00 |  |  |  |  |
|  |  | >2.08 | 56 | 4.4 | 25 | 4.4 | 1.09 | (0.72, | 1.65) | 0.676 | 0.89 |
|  | hsa-miR-4652-3p | <-1.77 | 32 | 2.5 | 21 | 3.7 | 1.71 | (1.10, | 2.65) | 0.017 | 0.56 |
|  |  | [-1.77, 2.08] | 1187 | 92.2 | 517 | 91.0 | 1.00 |  |  |  |  |
|  |  | >2.08 | 68 | 5.3 | 30 | 5.3 | 1.31 | (0.90, | 1.90) | 0.157 | 0.67 |
|  | hsa-miR-466 | <-1.77 | 47 | 3.7 | 32 | 5.6 | 1.61 | (1.12, | 2.32) | 0.010 | 0.56 |
|  |  | [-1.77, 2.08] | 1111 | 86.3 | 465 | 81.9 | 1.00 |  |  |  |  |
|  |  | >2.08 | 129 | 10.0 | 71 | 12.5 | 1.35 | (1.05, | 1.74) | 0.020 | 0.32 |
|  | hsa-miR-4730 | <-1.77 | 376 | 29.2 | 183 | 32.2 | 1.23 | (1.01, | 1.50) | 0.035 | 0.69 |
|  |  | [-1.77, 2.08] | 569 | 44.2 | 238 | 41.9 | 1.00 |  |  |  |  |
|  |  | >2.08 | 342 | 26.6 | 147 | 25.9 | 1.05 | (0.86, | 1.30) | 0.625 | 0.89 |
|  | hsa-miR-532-5p | <-1.77 | 43 | 3.3 | 27 | 4.8 | 1.02 | (0.68, | 1.52) | 0.938 | 0.94 |
|  |  | [-1.77, 2.08] | 793 | 61.6 | 371 | 65.3 | 1.00 |  |  |  |  |
|  |  | >2.08 | 451 | 35.0 | 170 | 29.9 | 0.78 | (0.65, | 0.93) | 0.007 | 0.18 |
|  | hsa-miR-590-5p | <-1.77 | 171 | 13.3 | 79 | 13.9 | 0.94 | (0.74, | 1.19) | 0.590 | 0.81 |
|  |  | [-1.77, 2.08] | 963 | 74.8 | 448 | 78.9 | 1.00 |  |  |  |  |
|  |  | >2.08 | 153 | 11.9 | 41 | 7.2 | 0.59 | (0.43, | 0.82) | 0.002 | 0.16 |
|  | hsa-miR-605 | <-1.77 | 199 | 15.5 | 71 | 12.5 | 0.86 | (0.67, | 1.11) | 0.248 | 0.81 |
|  |  | [-1.77, 2.08] | 1020 | 79.3 | 448 | 78.9 | 1.00 |  |  |  |  |
|  |  | >2.08 | 68 | 5.3 | 49 | 8.6 | 1.37 | (1.01, | 1.85) | 0.043 | 0.42 |
|  | hsa-miR-632 | <-1.77 | 225 | 17.5 | 88 | 15.5 | 0.79 | (0.63, | 1.00) | 0.049 | 0.81 |
|  |  | [-1.77, 2.08] | 882 | 68.5 | 415 | 73.1 | 1.00 |  |  |  |  |
|  |  | >2.08 | 180 | 14.0 | 65 | 11.4 | 0.74 | (0.57, | 0.97) | 0.027 | 0.32 |
|  | hsa-miR-645 | <-1.77 | 6 | 0.5 | 8 | 1.4 | 2.71 | (1.33, | 5.51) | 0.006 | 0.56 |
|  |  | [-1.77, 2.08] | 909 | 70.6 | 348 | 61.3 | 1.00 |  |  |  |  |
|  |  | >2.08 | 372 | 28.9 | 212 | 37.3 | 1.22 | (1.03, | 1.46) | 0.023 | 0.32 |
|  | hsa-miR-6514-3p | <-1.77 | 36 | 2.8 | 27 | 4.8 | 1.68 | (1.13, | 2.51) | 0.010 | 0.56 |
|  |  | [-1.77, 2.08] | 1224 | 95.1 | 532 | 93.7 | 1.00 |  |  |  |  |
|  |  | >2.08 | 27 | 2.1 | 9 | 1.6 | 1.06 | (0.55, | 2.06) | 0.859 | 0.90 |
|  | hsa-miR-934 | <-1.77 | 30 | 2.3 | 16 | 2.8 | 1.22 | (0.73, | 2.04) | 0.447 | 0.81 |
|  |  | [-1.77, 2.08] | 534 | 41.5 | 184 | 32.4 | 1.00 |  |  |  |  |
|  |  | >2.08 | 723 | 56.2 | 368 | 64.8 | 1.27 | (1.06, | 1.52) | 0.009 | 0.18 |
|  | hsa-miR-96-5p | <-1.77 | 0 | 0.0 | 0 | 0.0 |  |  |  |  |  |
|  |  | [-1.77, 2.08] | 1243 | 96.6 | 536 | 94.4 | 1.00 |  |  |  |  |
|  |  | >2.08 | 44 | 3.4 | 32 | 5.6 | 1.46 | (1.02, | 2.10) | 0.037 | 0.41 |
|  | hsa-miR-99b-5p | <-1.77 | 289 | 22.5 | 121 | 21.3 | 1.01 | (0.81, | 1.27) | 0.903 | 0.90 |
|  |  | [-1.77, 2.08] | 634 | 49.3 | 254 | 44.7 | 1.00 |  |  |  |  |
|  |  | >2.08 | 364 | 28.3 | 193 | 34.0 | 1.34 | (1.10, | 1.63) | 0.003 | 0.16 |
| Colon | hsa-miR-1 | <-1.77 | 33 | 4.2 | 18 | 5.2 | 0.76 | (0.46, | 1.25) | 0.284 | 0.99 |
|  |  | [-1.77, 2.08] | 748 | 94.7 | 310 | 90.1 | 1.00 |  |  |  |  |
|  |  | >2.08 | 9 | 1.1 | 16 | 4.7 | 1.91 | (1.14, | 3.18) | 0.013 | 0.16 |
|  | hsa-miR-124-3p | <-1.77 | 55 | 7.0 | 39 | 11.3 | 1.11 | (0.79, | 1.56) | 0.541 | 0.99 |
|  |  | [-1.77, 2.08] | 713 | 90.3 | 287 | 83.4 | 1.00 |  |  |  |  |
|  |  | >2.08 | 22 | 2.8 | 18 | 5.2 | 2.24 | (1.37, | 3.68) | 0.001 | 0.14 |
|  | hsa-miR-133a | <-1.77 | 23 | 2.9 | 15 | 4.4 | 0.88 | (0.52, | 1.51) | 0.646 | 0.99 |
|  |  | [-1.77, 2.08] | 760 | 96.2 | 315 | 91.6 | 1.00 |  |  |  |  |
|  |  | >2.08 | 7 | 0.9 | 14 | 4.1 | 1.88 | (1.07, | 3.28) | 0.028 | 0.20 |
|  | hsa-miR-143-5p | <-1.77 | 48 | 6.1 | 20 | 5.8 | 0.66 | (0.41, | 1.06) | 0.087 | 0.99 |
|  |  | [-1.77, 2.08] | 720 | 91.1 | 291 | 84.6 | 1.00 |  |  |  |  |
|  |  | >2.08 | 22 | 2.8 | 33 | 9.6 | 1.57 | (1.08, | 2.29) | 0.019 | 0.16 |
|  | hsa-miR-145-3p | <-1.77 | 18 | 2.3 | 12 | 3.5 | 1.11 | (0.61, | 2.02) | 0.727 | 0.99 |
|  |  | [-1.77, 2.08] | 767 | 97.1 | 319 | 92.7 | 1.00 |  |  |  |  |
|  |  | >2.08 | 5 | 0.6 | 13 | 3.8 | 3.02 | (1.71, | 5.31) | 0.000 | 0.14 |
|  | hsa-miR-2278 | <-1.77 | 83 | 10.5 | 39 | 11.3 | 1.55 | (1.09, | 2.20) | 0.015 | 0.80 |
|  |  | [-1.77, 2.08] | 590 | 74.7 | 249 | 72.4 | 1.00 |  |  |  |  |
|  |  | >2.08 | 117 | 14.8 | 56 | 16.3 | 1.18 | (0.88, | 1.59) | 0.276 | 0.47 |
|  | hsa-miR-30e-5p | <-1.77 | 293 | 37.1 | 128 | 37.2 | 0.70 | (0.55, | 0.88) | 0.003 | 0.80 |
|  |  | [-1.77, 2.08] | 384 | 48.6 | 172 | 50.0 | 1.00 |  |  |  |  |
|  |  | >2.08 | 113 | 14.3 | 44 | 12.8 | 0.63 | (0.45, | 0.89) | 0.008 | 0.16 |
|  | hsa-miR-31-5p | <-1.77 | 18 | 2.3 | 5 | 1.5 | 0.56 | (0.23, | 1.37) | 0.204 | 0.99 |
|  |  | [-1.77, 2.08] | 659 | 83.4 | 264 | 76.7 | 1.00 |  |  |  |  |
|  |  | >2.08 | 113 | 14.3 | 75 | 21.8 | 1.62 | (1.24, | 2.10) | 0.000 | 0.14 |
|  | hsa-miR-3622b-3p | <-1.77 | 23 | 2.9 | 9 | 2.6 | 0.92 | (0.45, | 1.86) | 0.810 | 0.99 |
|  |  | [-1.77, 2.08] | 590 | 74.7 | 240 | 69.8 | 1.00 |  |  |  |  |
|  |  | >2.08 | 177 | 22.4 | 95 | 27.6 | 1.34 | (1.05, | 1.71) | 0.020 | 0.16 |
|  | hsa-miR-362-5p | <-1.77 | 16 | 2.0 | 7 | 2.0 | 0.59 | (0.27, | 1.26) | 0.173 | 0.99 |
|  |  | [-1.77, 2.08] | 585 | 74.1 | 275 | 79.9 | 1.00 |  |  |  |  |
|  |  | >2.08 | 189 | 23.9 | 62 | 18.0 | 0.66 | (0.50, | 0.87) | 0.004 | 0.14 |
|  | hsa-miR-374a-5p | <-1.77 | 10 | 1.3 | 6 | 1.7 | 0.73 | (0.32, | 1.66) | 0.454 | 0.99 |
|  |  | [-1.77, 2.08] | 631 | 79.9 | 287 | 83.4 | 1.00 |  |  |  |  |
|  |  | >2.08 | 149 | 18.9 | 51 | 14.8 | 0.73 | (0.54, | 0.99) | 0.043 | 0.24 |
|  | hsa-miR-378g | <-1.77 | 156 | 19.7 | 77 | 22.4 | 1.15 | (0.89, | 1.49) | 0.297 | 0.99 |
|  |  | [-1.77, 2.08] | 586 | 74.2 | 238 | 69.2 | 1.00 |  |  |  |  |
|  |  | >2.08 | 48 | 6.1 | 29 | 8.4 | 1.82 | (1.22, | 2.70) | 0.003 | 0.14 |
|  | hsa-miR-424-5p | <-1.77 | 20 | 2.5 | 5 | 1.5 | 0.71 | (0.29, | 1.72) | 0.445 | 0.99 |
|  |  | [-1.77, 2.08] | 570 | 72.2 | 267 | 77.6 | 1.00 |  |  |  |  |
|  |  | >2.08 | 200 | 25.3 | 72 | 20.9 | 0.72 | (0.55, | 0.95) | 0.021 | 0.16 |
|  | hsa-miR-4300 | <-1.77 | 112 | 14.2 | 35 | 10.2 | 0.64 | (0.45, | 0.92) | 0.015 | 0.80 |
|  |  | [-1.77, 2.08] | 564 | 71.4 | 259 | 75.3 | 1.00 |  |  |  |  |
|  |  | >2.08 | 114 | 14.4 | 50 | 14.5 | 1.01 | (0.74, | 1.37) | 0.966 | 0.97 |
|  | hsa-miR-4642 | <-1.77 | 18 | 2.3 | 13 | 3.8 | 1.79 | (1.02, | 3.13) | 0.041 | 0.99 |
|  |  | [-1.77, 2.08] | 731 | 92.5 | 315 | 91.6 | 1.00 |  |  |  |  |
|  |  | >2.08 | 41 | 5.2 | 16 | 4.7 | 1.09 | (0.66, | 1.82) | 0.730 | 0.73 |
|  | hsa-miR-466 | <-1.77 | 22 | 2.8 | 14 | 4.1 | 1.52 | (0.87, | 2.67) | 0.143 | 0.99 |
|  |  | [-1.77, 2.08] | 723 | 91.5 | 295 | 85.8 | 1.00 |  |  |  |  |
|  |  | >2.08 | 45 | 5.7 | 35 | 10.2 | 1.55 | (1.09, | 2.21) | 0.015 | 0.16 |
|  | hsa-miR-548aa | <-1.77 | 62 | 7.8 | 33 | 9.6 | 1.53 | (1.07, | 2.21) | 0.021 | 0.86 |
|  |  | [-1.77, 2.08] | 680 | 86.1 | 282 | 82.0 | 1.00 |  |  |  |  |
|  |  | >2.08 | 48 | 6.1 | 29 | 8.4 | 1.45 | (0.98, | 2.12) | 0.060 | 0.26 |
|  | hsa-miR-548am-5p | <-1.77 | 19 | 2.4 | 10 | 2.9 | 1.96 | (1.04, | 3.71) | 0.038 | 0.99 |
|  |  | [-1.77, 2.08] | 750 | 94.9 | 322 | 93.6 | 1.00 |  |  |  |  |
|  |  | >2.08 | 21 | 2.7 | 12 | 3.5 | 1.09 | (0.61, | 1.95) | 0.768 | 0.77 |
|  | hsa-miR-548aw | <-1.77 | 21 | 2.7 | 11 | 3.2 | 0.96 | (0.52, | 1.75) | 0.887 | 0.99 |
|  |  | [-1.77, 2.08] | 763 | 96.6 | 326 | 94.8 | 1.00 |  |  |  |  |
|  |  | >2.08 | 6 | 0.8 | 7 | 2.0 | 3.04 | (1.42, | 6.48) | 0.004 | 0.14 |
|  | hsa-miR-632 | <-1.77 | 142 | 18.0 | 52 | 15.1 | 0.72 | (0.53, | 0.97) | 0.031 | 0.99 |
|  |  | [-1.77, 2.08] | 528 | 66.8 | 248 | 72.1 | 1.00 |  |  |  |  |
|  |  | >2.08 | 120 | 15.2 | 44 | 12.8 | 0.71 | (0.51, | 0.98) | 0.038 | 0.24 |
|  | hsa-miR-645 | <-1.77 | 5 | 0.6 | 6 | 1.7 | 2.98 | (1.30, | 6.79) | 0.010 | 0.80 |
|  |  | [-1.77, 2.08] | 576 | 72.9 | 213 | 61.9 | 1.00 |  |  |  |  |
|  |  | >2.08 | 209 | 26.5 | 125 | 36.3 | 1.33 | (1.06, | 1.66) | 0.014 | 0.16 |
|  | hsa-miR-671-3p | <-1.77 | 42 | 5.3 | 22 | 6.4 | 1.25 | (0.80, | 1.95) | 0.318 | 0.99 |
|  |  | [-1.77, 2.08] | 727 | 92.0 | 310 | 90.1 | 1.00 |  |  |  |  |
|  |  | >2.08 | 21 | 2.7 | 12 | 3.5 | 2.17 | (1.15, | 4.11) | 0.017 | 0.16 |
|  | hsa-miR-6722-5p | <-1.77 | 15 | 1.9 | 6 | 1.7 | 0.83 | (0.37, | 1.87) | 0.647 | 0.99 |
|  |  | [-1.77, 2.08] | 763 | 96.6 | 322 | 93.6 | 1.00 |  |  |  |  |
|  |  | >2.08 | 12 | 1.5 | 16 | 4.7 | 1.70 | (1.02, | 2.83) | 0.042 | 0.24 |
|  | hsa-miR-934 | <-1.77 | 24 | 3.0 | 11 | 3.2 | 1.07 | (0.57, | 1.99) | 0.836 | 0.99 |
|  |  | [-1.77, 2.08] | 347 | 43.9 | 116 | 33.7 | 1.00 |  |  |  |  |
|  |  | >2.08 | 419 | 53.0 | 217 | 63.1 | 1.34 | (1.07, | 1.70) | 0.012 | 0.16 |
|  | hsa-miR-99b-5p | <-1.77 | 136 | 17.2 | 57 | 16.6 | 0.79 | (0.57, | 1.09) | 0.143 | 0.99 |
|  |  | [-1.77, 2.08] | 439 | 55.6 | 170 | 49.4 | 1.00 |  |  |  |  |
|  |  | >2.08 | 215 | 27.2 | 117 | 34.0 | 1.29 | (1.01, | 1.66) | 0.041 | 0.24 |
| Rectal | hsa-miR-124-3p | <-1.77 | 15 | 3.0 | 15 | 6.7 | 1.70 | (1.00, | 2.88) | 0.048 | 0.41 |
|  |  | [-1.77, 2.08] | 471 | 94.8 | 201 | 89.7 | 1.00 |  |  |  |  |
|  |  | >2.08 | 11 | 2.2 | 8 | 3.6 | 1.79 | (0.88, | 3.66) | 0.111 | 0.49 |
|  | hsa-miR-1915-5p | <-1.77 | 29 | 5.8 | 7 | 3.1 | 0.43 | (0.20, | 0.91) | 0.028 | 0.37 |
|  |  | [-1.77, 2.08] | 452 | 90.9 | 208 | 92.9 | 1.00 |  |  |  |  |
|  |  | >2.08 | 16 | 3.2 | 9 | 4.0 | 1.50 | (0.76, | 2.99) | 0.242 | 0.52 |
|  | hsa-miR-199b-5p | <-1.77 | 28 | 5.6 | 14 | 6.3 | 0.95 | (0.54, | 1.66) | 0.850 | 0.85 |
|  |  | [-1.77, 2.08] | 276 | 55.5 | 140 | 62.5 | 1.00 |  |  |  |  |
|  |  | >2.08 | 193 | 38.8 | 70 | 31.3 | 0.73 | (0.54, | 0.98) | 0.037 | 0.40 |
|  | hsa-miR-203a | <-1.77 | 53 | 10.7 | 27 | 12.1 | 0.87 | (0.55, | 1.37) | 0.544 | 0.71 |
|  |  | [-1.77, 2.08] | 110 | 22.1 | 65 | 29.0 | 1.00 |  |  |  |  |
|  |  | >2.08 | 334 | 67.2 | 132 | 58.9 | 0.71 | (0.53, | 0.97) | 0.030 | 0.40 |
|  | hsa-miR-2278 | <-1.77 | 83 | 16.7 | 48 | 21.4 | 1.10 | (0.79, | 1.54) | 0.568 | 0.71 |
|  |  | [-1.77, 2.08] | 340 | 68.4 | 156 | 69.6 | 1.00 |  |  |  |  |
|  |  | >2.08 | 74 | 14.9 | 20 | 8.9 | 0.59 | (0.37, | 0.95) | 0.030 | 0.40 |
|  | hsa-miR-299-3p | <-1.77 | 82 | 16.5 | 44 | 19.6 | 1.51 | (1.08, | 2.12) | 0.017 | 0.37 |
|  |  | [-1.77, 2.08] | 382 | 76.9 | 169 | 75.4 | 1.00 |  |  |  |  |
|  |  | >2.08 | 33 | 6.6 | 11 | 4.9 | 1.05 | (0.57, | 1.95) | 0.870 | 0.87 |
|  | hsa-miR-3149 | <-1.77 | 152 | 30.6 | 84 | 37.5 | 1.40 | (1.03, | 1.88) | 0.029 | 0.37 |
|  |  | [-1.77, 2.08] | 207 | 41.6 | 92 | 41.1 | 1.00 |  |  |  |  |
|  |  | >2.08 | 138 | 27.8 | 48 | 21.4 | 0.90 | (0.63, | 1.29) | 0.559 | 0.65 |
|  | hsa-miR-3609 | <-1.77 | 81 | 16.3 | 50 | 22.3 | 1.47 | (1.05, | 2.05) | 0.025 | 0.37 |
|  |  | [-1.77, 2.08] | 296 | 59.6 | 123 | 54.9 | 1.00 |  |  |  |  |
|  |  | >2.08 | 120 | 24.1 | 51 | 22.8 | 0.85 | (0.61, | 1.19) | 0.340 | 0.57 |
|  | hsa-miR-3615 | <-1.77 | 68 | 13.7 | 41 | 18.3 | 1.08 | (0.76, | 1.53) | 0.679 | 0.77 |
|  |  | [-1.77, 2.08] | 415 | 83.5 | 169 | 75.4 | 1.00 |  |  |  |  |
|  |  | >2.08 | 14 | 2.8 | 14 | 6.3 | 2.28 | (1.28, | 4.09) | 0.005 | 0.31 |
|  | hsa-miR-374b-5p | <-1.77 | 12 | 2.4 | 6 | 2.7 | 1.34 | (0.59, | 3.05) | 0.487 | 0.71 |
|  |  | [-1.77, 2.08] | 309 | 62.2 | 156 | 69.6 | 1.00 |  |  |  |  |
|  |  | >2.08 | 176 | 35.4 | 62 | 27.7 | 0.71 | (0.53, | 0.96) | 0.026 | 0.40 |
|  | hsa-miR-378e | <-1.77 | 65 | 13.1 | 31 | 13.8 | 0.84 | (0.57, | 1.25) | 0.396 | 0.64 |
|  |  | [-1.77, 2.08] | 329 | 66.2 | 163 | 72.8 | 1.00 |  |  |  |  |
|  |  | >2.08 | 103 | 20.7 | 30 | 13.4 | 0.57 | (0.38, | 0.85) | 0.005 | 0.31 |
|  | hsa-miR-378j | <-1.77 | 15 | 3.0 | 7 | 3.1 | 1.16 | (0.54, | 2.52) | 0.704 | 0.78 |
|  |  | [-1.77, 2.08] | 441 | 88.7 | 205 | 91.5 | 1.00 |  |  |  |  |
|  |  | >2.08 | 41 | 8.2 | 12 | 5.4 | 0.53 | (0.28, | 0.98) | 0.043 | 0.42 |
|  | hsa-miR-3922-5p | <-1.77 | 60 | 12.1 | 38 | 17.0 | 1.55 | (1.08, | 2.21) | 0.016 | 0.37 |
|  |  | [-1.77, 2.08] | 371 | 74.6 | 170 | 75.9 | 1.00 |  |  |  |  |
|  |  | >2.08 | 66 | 13.3 | 16 | 7.1 | 0.75 | (0.44, | 1.28) | 0.286 | 0.54 |
|  | hsa-miR-4282 | <-1.77 | 144 | 29.0 | 49 | 21.9 | 0.68 | (0.49, | 0.94) | 0.019 | 0.37 |
|  |  | [-1.77, 2.08] | 277 | 55.7 | 149 | 66.5 | 1.00 |  |  |  |  |
|  |  | >2.08 | 76 | 15.3 | 26 | 11.6 | 0.69 | (0.45, | 1.05) | 0.081 | 0.49 |
|  | hsa-miR-4300 | <-1.77 | 43 | 8.7 | 33 | 14.7 | 1.51 | (1.03, | 2.21) | 0.034 | 0.38 |
|  |  | [-1.77, 2.08] | 375 | 75.5 | 162 | 72.3 | 1.00 |  |  |  |  |
|  |  | >2.08 | 79 | 15.9 | 29 | 12.9 | 0.83 | (0.56, | 1.23) | 0.346 | 0.57 |
|  | hsa-miR-4324 | <-1.77 | 99 | 19.9 | 53 | 23.7 | 1.51 | (1.09, | 2.09) | 0.014 | 0.37 |
|  |  | [-1.77, 2.08] | 332 | 66.8 | 129 | 57.6 | 1.00 |  |  |  |  |
|  |  | >2.08 | 66 | 13.3 | 42 | 18.8 | 1.32 | (0.93, | 1.88) | 0.125 | 0.49 |
|  | hsa-miR-451b | <-1.77 | 35 | 7.0 | 25 | 11.2 | 1.65 | (1.08, | 2.52) | 0.021 | 0.37 |
|  |  | [-1.77, 2.08] | 441 | 88.7 | 191 | 85.3 | 1.00 |  |  |  |  |
|  |  | >2.08 | 21 | 4.2 | 8 | 3.6 | 0.73 | (0.35, | 1.52) | 0.405 | 0.62 |
|  | hsa-miR-4654 | <-1.77 | 93 | 18.7 | 40 | 17.9 | 0.95 | (0.66, | 1.35) | 0.762 | 0.81 |
|  |  | [-1.77, 2.08] | 254 | 51.1 | 134 | 59.8 | 1.00 |  |  |  |  |
|  |  | >2.08 | 150 | 30.2 | 50 | 22.3 | 0.68 | (0.49, | 0.95) | 0.023 | 0.40 |
|  | hsa-miR-4682 | <-1.77 | 62 | 12.5 | 46 | 20.5 | 1.69 | (1.20, | 2.38) | 0.002 | 0.37 |
|  |  | [-1.77, 2.08] | 373 | 75.1 | 146 | 65.2 | 1.00 |  |  |  |  |
|  |  | >2.08 | 62 | 12.5 | 32 | 14.3 | 1.46 | (0.98, | 2.17) | 0.063 | 0.49 |
|  | hsa-miR-518c-5p | <-1.77 | 75 | 15.1 | 45 | 20.1 | 1.51 | (1.08, | 2.11) | 0.017 | 0.37 |
|  |  | [-1.77, 2.08] | 360 | 72.4 | 163 | 72.8 | 1.00 |  |  |  |  |
|  |  | >2.08 | 62 | 12.5 | 16 | 7.1 | 0.74 | (0.44, | 1.24) | 0.248 | 0.52 |
|  | hsa-miR-548aa | <-1.77 | 52 | 10.5 | 29 | 12.9 | 0.90 | (0.61, | 1.35) | 0.622 | 0.75 |
|  |  | [-1.77, 2.08] | 378 | 76.1 | 178 | 79.5 | 1.00 |  |  |  |  |
|  |  | >2.08 | 67 | 13.5 | 17 | 7.6 | 0.52 | (0.31, | 0.86) | 0.012 | 0.34 |
|  | hsa-miR-590-5p | <-1.77 | 46 | 9.3 | 19 | 8.5 | 0.76 | (0.47, | 1.24) | 0.272 | 0.59 |
|  |  | [-1.77, 2.08] | 397 | 79.9 | 194 | 86.6 | 1.00 |  |  |  |  |
|  |  | >2.08 | 54 | 10.9 | 11 | 4.9 | 0.43 | (0.23, | 0.79) | 0.006 | 0.31 |
|  | hsa-miR-6081 | <-1.77 | 66 | 13.3 | 32 | 14.3 | 1.02 | (0.70, | 1.48) | 0.936 | 0.94 |
|  |  | [-1.77, 2.08] | 381 | 76.7 | 180 | 80.4 | 1.00 |  |  |  |  |
|  |  | >2.08 | 50 | 10.1 | 12 | 5.4 | 0.53 | (0.29, | 0.95) | 0.033 | 0.40 |
|  | hsa-miR-6515-5p | <-1.77 | 356 | 71.6 | 167 | 74.6 | 1.02 | (0.73, | 1.44) | 0.892 | 0.89 |
|  |  | [-1.77, 2.08] | 124 | 24.9 | 45 | 20.1 | 1.00 |  |  |  |  |
|  |  | >2.08 | 17 | 3.4 | 12 | 5.4 | 1.92 | (1.01, | 3.67) | 0.047 | 0.43 |
|  | hsa-miR-675-5p | <-1.77 | 0 | 0.0 | 0 | 0.0 |  |  |  |  |  |
|  |  | [-1.77, 2.08] | 492 | 99.0 | 216 | 96.4 | 1.00 |  |  |  |  |
|  |  | >2.08 | 5 | 1.0 | 8 | 3.6 | 3.13 | (1.51, | 6.47) | 0.002 | 0.31 |
